# Adult stem cell deficits drive *Slc29a3* disorders in mice

**DOI:** 10.1038/s41467-019-10925-3

**Published:** 2019-07-03

**Authors:** Sreenath Nair, Anne M. Strohecker, Avinash K. Persaud, Bhawana Bissa, Shanmugam Muruganandan, Craig McElroy, Rakesh Pathak, Michelle Williams, Radhika Raj, Amal Kaddoumi, Alex Sparreboom, Aaron M. Beedle, Rajgopal Govindarajan

**Affiliations:** 1 Division of Pharmaceutics and Pharmaceutical Chemistry, College of Pharmacy, Ohio State University, Columbus, OH 43210 USA; 2 Department of Cancer Biology and Genetics, College of Medicine, Ohio State University, Columbus, OH 43210 USA; 3 Molecular Biology and Cancer Genetics, Ohio State University Comprehensive Cancer Center, Ohio State University, Columbus, OH 43210 USA; 4 Department of Radiology, Ohio State University, Columbus, OH 43210 USA; 5 Department of Drug Discovery and Development, Harrison School of Pharmacy, Auburn University, Auburn, AL 36849 USA; 6 Department of Pharmaceutical Sciences, SUNY Binghamton University, Binghamton, NY 13902 USA; 7 Translational Therapeutics, Ohio State University Comprehensive Cancer Center, Ohio State University, Columbus, OH 43210 USA

**Keywords:** Macroautophagy, Lysosomes, Haematopoietic stem cells, Mesenchymal stem cells

## Abstract

Mutations exclusively in equilibrative nucleoside transporter 3 (ENT3), the only intracellular nucleoside transporter within the solute carrier 29 (*SLC29*) gene family, cause an expanding spectrum of human genetic disorders (e.g., H syndrome, PHID syndrome, and SHML/RDD syndrome). Here, we identify adult stem cell deficits that drive ENT3-related abnormalities in mice. ENT3 deficiency alters hematopoietic and mesenchymal stem cell fates; the former leads to stem cell exhaustion, and the latter leads to breaches of mesodermal tissue integrity. The molecular pathogenesis stems from the loss of lysosomal adenosine transport, which impedes autophagy-regulated stem cell differentiation programs via misregulation of the AMPK-mTOR-ULK axis. Furthermore, mass spectrometry-based metabolomics and bioenergetics studies identify defects in fatty acid utilization, and alterations in mitochondrial bioenergetics can additionally propel stem cell deficits. Genetic, pharmacologic and stem cell interventions ameliorate ENT3-disease pathologies and extend the lifespan of ENT3-deficient mice. These findings delineate a primary pathogenic basis for the development of ENT3 spectrum disorders and offer critical mechanistic insights into treating human ENT3-related disorders.

## Abstract (web summary)

Mutations in equilibrative nucleoside transporter 3 (ENT3), encoded by *SLC29A3*, cause a spectrum of human genetic disorders. Here, the authors show altered haematopoietic stem cell and mesenchymal stem cell fates in ENT3-deficient mice, due to misregulation of the AMPK-mTOR-ULK axis.

## Introduction

A series of human genetic disorders with dermatological, musculoskeletal, endocrinological, and hematological abnormalities have been reported over the past decades under various diagnostic terms, namely gonodermatosis, pigmented hypertrichosis with insulin-dependent diabetes mellitus (PHID), familial histiocytosis (FHC), sinus histiocytosis with massive lymphadenopathy (SHML), Rosai-Dorfman Disease (RDD), and familial plasmacytosis^1–4^. Despite the clinical similarities, the genotype-to-phenotype link and molecular pathogenesis remain unknown. In 2008, three single-nucleotide changes in *SLC29A3* (1279 G > A, 1309 G > A, 1045delC) were reported as causative for an autosomal-recessive gonodermatosis called ‘H syndrome’^5^. H syndrome refers to hyperpigmentation, hypertrichosis, hepatosplenomegaly, heart anomalies, hearing loss, hypogonadism, low-height, and hyperglycemia^5^. In 2009, five mutations in *SLC29A3* (347 T > G, 940delT, 1309 G > A, 1330 G > T, and 1346 C > G) were documented as causative for PHID^6^. PHID is associated with diabetes mellitus, lipodystrophy, and pigmented hypertrichosis with abnormal hematopoietic and myelofibrotic features^6^. In 2010, three mutations in *SLC29A3* (300 + 1 G > A, 307delT, 1309 G > A) were reported for FHC, SHML/FHC, and RDD syndromes, which exhibit stunted growth, lymphadenopathy, and histiocytosis^7^. Presently, more than 20 disease-causing mutations in *SLC29A3* and a broader spectrum of clinical manifestations have been reported^8–16^.

The *SLC29* gene family encodes four equilibrative nucleoside transporters (ENTs; ENT1 to ENT4) that facilitate the membrane translocation of hydrophilic nucleosides to regulate salvage DNA synthesis and purinergic signaling^17^. The *SLC29A3* gene (encoding ENT3) on chromosome 10 contains six exons, with mutation sites concentrated in the last exon encoding almost the entire carboxyl-half of ENT3^18^. ENT3 is unique as the only intracellular nucleoside transporter in the family with putative localization in late endosomes, lysosomes, and mitochondria (other human ENTs (hENTs) primarily function at the cell surface)^19,20^. In 2012, the roles of ENT3 in lysosomal homeostasis and macrophage biology were elucidated^21^. While impaired macrophage dysfunction accounts for some pathology in ENT3 disorders (e.g., histiocytosis), the molecular mechanisms of most disease manifestations are unknown. Nonetheless, the monogenic nature of ENT3 disorders suggests additional unifying mechanisms underlying ENT3 disease pathologies. Previous studies in our laboratory demonstrated that *SLC29A3* disease mutations impair nucleoside transport, subcellular localization, protein stability, and pH sensing ability^18,20,22,23^. Studies from other laboratories also indicated a correlation between human disease severity and residual hENT3 activity because mutations with a partial loss of function have hypomorphic features^24^. Recently, the transport features and developmental expression pattern of the mouse ortholog of hENT3 (mENT3) were characterized^7,19^. Intriguingly, mENT3 showed similar kinetic features to hENT3 and tissue expression consistent with the pattern of dysfunction in humans.

Here, we establish a functional link between ENT3 and the AMPK-mTOR-ULK-regulated lysosome-autophagy pathway and demonstrate that adult stem cell deficits primarily drive *Slc29a3* disorders in mice because of the loss of lysosomal transport of adenosine, a cargo transported through ENT3. Genetic, pharmacological, and stem cell interventions ameliorate the disease severity in mice, with therapeutic implications for human disorders.

## Results

### Slc29a3^−/−^ mice recapitulate human phenotypes

**Fig. 1:**
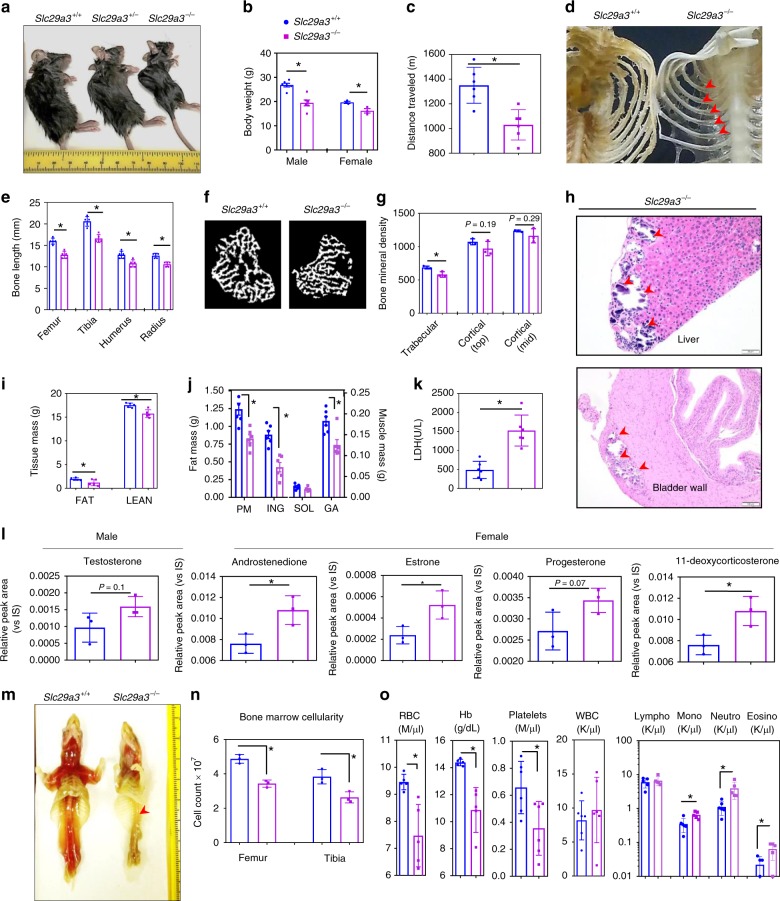
*Slc29a3*^−/−^ mice recapitulate the hENT3 disease features. *Slc29a3*^−/−^ mice presented a weak, hunched-back appearance (**a**) and weighed less than *Slc29a3*^+/+^ mice (males, *n* = 6; females, *n* = 3; mean ± SEM) (**b**). Treadmill run time to exhaustion (*n* = 6, mean ± SEM) (**c**). Translucent appearance of ribs in *Slc29a3*^−/−^ mice (**d**). Lengths of the femur, tibia, humerus, and radius measured using a Vernier caliper (*n* = 5, mean ± SEM) (**e**). Bone microarchitecture and mineral density assessed by microCT analysis (**f**) show reduced trabecular bone mineral density in *Slc29a3*^−/−^ mice (*n* = 3, mean ± SEM) (**g**). Displays ectopic mineralization of the liver and bladder wall (*purple*; liver, Scale bar: 50 μm; bladder wall, Scale bar: 100 μm) in *Slc9a3*^−/−^ mice (**h**). EchoMRI analysis of body fat and lean tissue composition (*n* = 5, mean ± SEM) (**i**). Weights of freshly isolated parametrial (PM) and inguinal (ING) fat pads (left axis), and soleus (SOL) and gastrocnemius (GA) skeletal muscles (right axis) (*n* = 6, mean ± SEM) (**j**). Increased plasma LDH levels in *Slc29a3*^−/−^ mice (*n* = 6, mean ± SEM) (**k**). Mass spectrometric analysis of sex steroid hormones (males, *n* = 3; females, *n* = 3, mean ± SEM). IS, internal standard (**l**). Medullary hematopoiesis examined at necropsy (**m**) and bone marrow cellularity (**n**) (*n* = 3, mean ± SEM). RBC counts (*n* = 5, mean ± SEM), hemoglobin concentration (*n* = 5, mean ± SEM), and platelet counts (*n* = 6, mean ± SEM) were significantly lower, while the counts of monocytes (Mono) (*n* = 5, mean ± SEM), neutrophils (Neutro) (*n* = 5, mean ± SEM) and eosinophils (Eosino) (*n* = 5, mean ± SEM) were significantly elevated in *Slc29a3*^−/−^ mice (**o**). All phenotypes assessed on *Slc29a3*^−/−^ mice at 12 weeks age compared with littermate *Slc29a3*^+/+^ controls. Arrowhead(s) point at abnormalities. Statistical analyses were performed by ANOVA with Tukey’s multiple comparisons post-test and two-tailed Student’s *t-*test. *P < 0.05. blue circles, *Slc29a3*^+/+^; magenta squares, *Slc29a3*^−/−^. Source data are provided as a Source Data file

The mENT3 knockout by gene trap of exons 1 and 2 (*Slc29a3*^−/−^; B6N.129S5-*Slc29a3*tm1Lex/Mmcd) generated mice with an apparently normal phenotype until 10–12 weeks of age^21^. However, mice exhibited profound deterioration of health after 12 weeks with overt manifestations of several seemingly unrelated disorders resulting in ~90% mortality at 18–20 weeks. At 12 weeks of age, *Slc29a3*^−/−^ mice demonstrated retarded growth and endurance capacity (Fig. 1a–c). Approximately 50–75% of mice displayed a characteristic ‘hunchback’’ kyphosis, hypertrichosis, malocclusion, and skeletal deformities consisting of impaired bone and cartilage development (Fig. 1a, d, e and Supplementary Fig. 1a, b). The microCT analysis identified a loss of trabecular bone mineral density (Fig. 1f, g). A third of *Slc29a3*^−/−^ mice presented with penile or vaginal prolapse with the mineralization of soft tissues (Fig. 1h). EchoMRI and necropsy revealed reductions in fat and lean mass (Fig. 1i, j), and elevated plasma lactate dehydrogenase suggested the presence of muscle damage (Fig. 1k). Many *Slc29a3*^−/−^ mice demonstrated hypogonadism (Supplementary Fig. 1c) and infertility, and, if bred, had ~2–3 pups/litter with quick cessation of breeding. Mass spectrometry analysis of mouse plasma revealed endocrinopathy as a possible source of reproductive deficiency (Fig. 1l).

At necropsy, the bone medulla was pale in appearance with reduced bone marrow cellularity (Fig. 1m, n) reminiscent of abnormal hematopoietic features reported in humans^25^. The spleen (and occasionally liver) showed massive enlargements (Supplementary Fig. 1d)^21^. Histopathological examination revealed severe histiocytosis in numerous organs^21^ (Supplementary Fig. 1e). Additionally, the spleen, lymph nodes, and thymus showed lymphoid cell death (Supplementary Fig. 1f). Enlarged lymph nodes also showed architectural effacement with sinusoidal infiltration of histiocytes admixed with tingible body macrophages (Supplementary Fig. 1f). Blood parameters revealed anemia, hemoglobinemia, thrombocytopenia, and leukocytosis (Fig. 1o). Overall, the abnormalities in *Slc29a3*^−/−^ mice displayed striking similarities to several clinical features reported in human *SLC29A3* disorders (Supplementary Fig. 2).

### Stem cell deficits drive ENT3-related dysfunctions

**Fig. 2:**
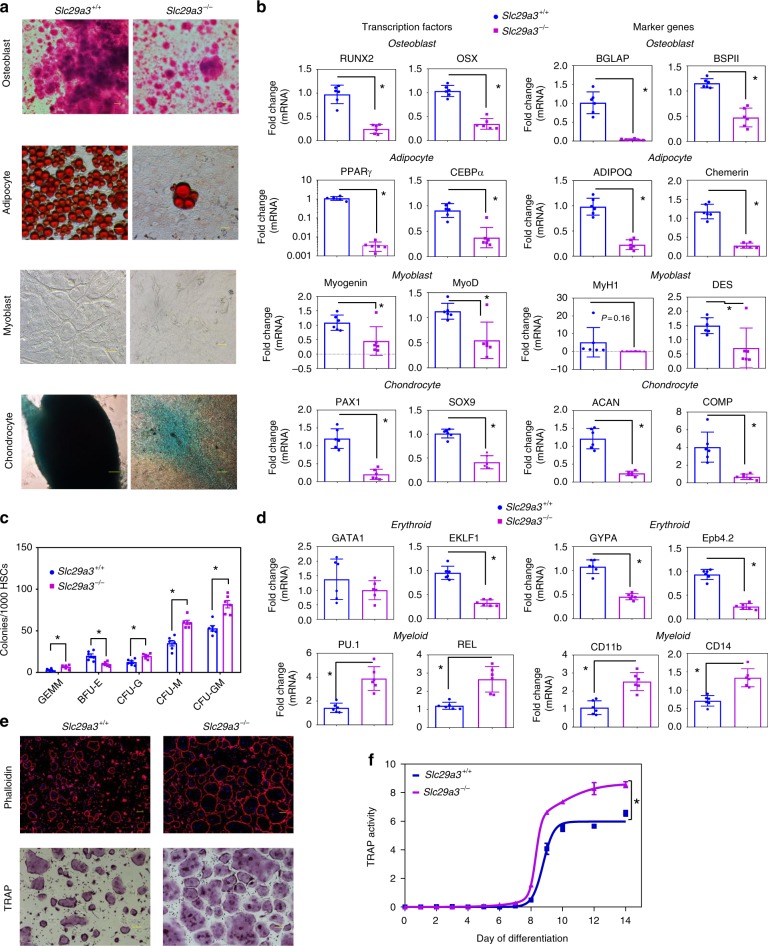
ENT3 is essential for stem cell differentiation. MSC differentiation into osteoblasts (mineralized nodules; pink), adipocytes (lipid droplets; red), myoblasts (myotube formation), and chondrocytes (cartilage nodule; bluish green). Scale bar: 100 μm (**a**). mRNA expression of transcription factors and markers after 14 (osteoblasts and adipocytes) and 28 (myoblasts and chondrocytes) days of MSC differentiation (*n* = 6, mean ± SEM) (**b**). HSC colony-forming cell assay (*n* = 6, mean ± SEM) (**c**) and transcript expression after 14 days of differentiation (*n* = 6, mean ± SEM) (**d**). Osteoclast formation (actin ring; red, above) (Scale bar: 100 μm) (**e**) and TRAP (tartrate-resistant acid phosphatase) expression (purple; below) (Scale bar: 100 μm) (**e**) and secretion with HSC differentiation (*n* = 6, mean ± SEM) (**f**). GEMM, granulocyte, erythrocyte, monocyte, megakaryocyte; BFU-E, burst-forming unit-erythroid; CFU, colony-forming unit; CFU-M, CFU-macrophage; CFU-G, CFU-granulocyte; CFU-GM, CFU-granulocyte/macrophage. Statistical analyses were performed using ANOVA with Tukey’s multiple comparisons post-test and two-tailed Student’s *t-*test. *P < 0.05. Source data are provided as a Source Data file

Adult stem cells are crucial for the maintenance and repair of adult tissues^26^. The phenotypes observed in several mesenchymal (bone, cartilage, muscle, fat) and hematopoietic (blood cells and blood-forming tissues) tissues (Fig. 1 and Supplementary Fig. 1) suggested a possible defect arising from common adult mesenchymal stem cells (MSCs) and hematopoietic stem cells (HSCs). Therefore, we investigated the putative roles for ENT3 in the differentiation capabilities of MSCs and HSCs. When forced to differentiate into each of the mesenchymal lineages, *Slc29a3*^−/−^ MSCs exhibited distinct deficits in forming mesenchymal lineages, osteoblasts, adipocytes, myocytes, and chondrocytes (Fig. 2a), with the reduction in lineage-specific markers (Fig. 2b). These findings suggested a common defect in ENT3-deficient MSCs for lineage specification resulting in widespread defects. Likewise, when *Slc29a3*^−/−^ HSCs were induced to differentiate into multiple lineages, a distinct skewing toward myeloid (G/M/GM/GEMM) over erythroid (E) commitment was observed (Fig. 2c). The induction of CFU-GEMM indicated that HSC differentiation is altered from hierarchically more primitive cell types^27^; however, the reciprocal induction of G/M/GM factors at the expense of E factors (Fig. 2d) also suggested a role for transcriptional reprogramming in skewing the balance toward myeloid production^28^. Nevertheless, the identification of bona fide erythroid-to-myeloid skewing provided a compelling explanation for the exacerbated formation of histiocytes and leukocytes with accompanied anemia and thrombocytopenia in *Slc29a3*^−/−^ mice. Consistent with the increased ability of HSCs to differentiate into CFU-M, increased osteoclastogenesis from CFU-M (Fig. 2e, f) further explained the occurrence of skeletal deformities, aberrant bone mineralization, and ectopic mineralization in *Slc29a3*^−/−^ mice.

**Fig. 3:**
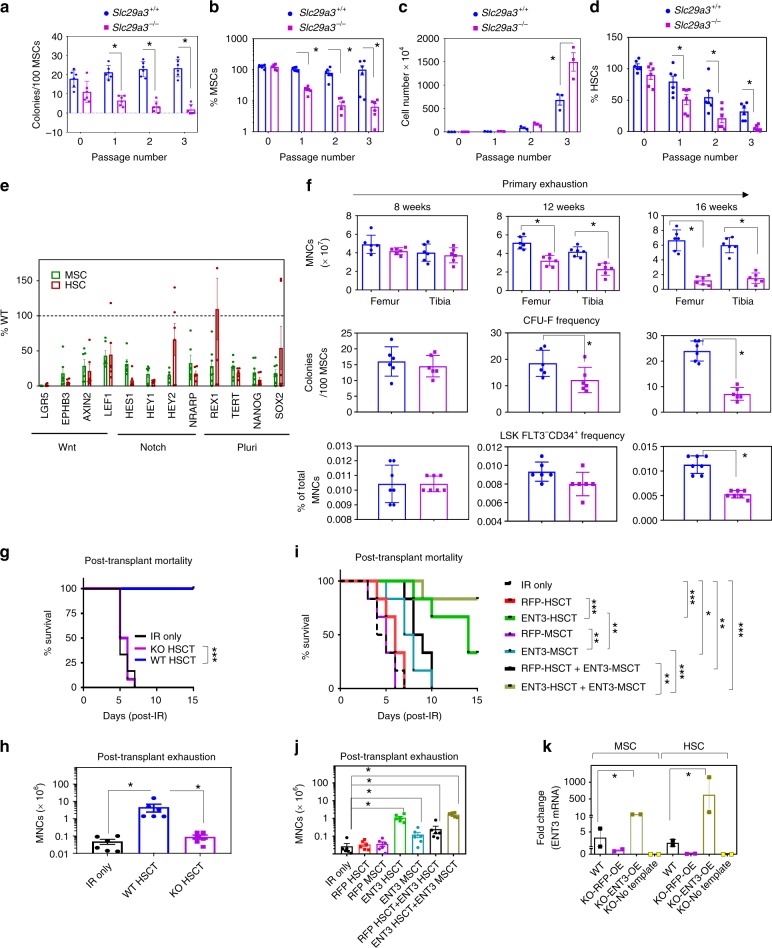
ENT3 is essential for self-renewal. MSC clonogenicity when subjected to serial passage (*n* = 6, mean ± SEM) (**a**). Percent cells expressing MSC markers after each passage of serial cloning (*n* = 6, mean ± SEM) (**b**). Culture expansion capacity of HSCs (*n* = 3, mean ± SEM) (**c**). Percent of cells expressing HSC markers after each passage (*n* = 6, mean ± SEM) (**d**). Expression of Wnt and Notch targets and pluripotency (pluri) marker genes in *Slc29a3*^−/−^ MSCs and HSCs (*n* = 6, mean ± SEM) (**e**). HSCs and MSCs were derived from 12-weeks-old animals (**a**–**e**). Cellularity (*n* = 6, mean ± SEM), MSC CFU-F (*n* = 6, mean ± SEM) and LSK FLT3^−^ CD34^+^ (*n* = 7, mean ± SEM) frequencies in bone marrow measured in different age groups (). MNC, mononuclear cells; CFU-F, CFU-Fibroblast (**f**). Ability of HSCs (1 × 10^4^ cells) to rescue radiation lethality in *Slc29a3*^−/−^ mice after transplantation presented as Kaplan–Meier survival curves (*n* = 6/group, ***P < 0.001; Mantel-Cox test) (**g**) and post transplantation bone marrow cellularity (*n* = 6, mean ± SEM) (**h**). Ability of *Slc29a3*^−/−^ HSCs (1 × 10^4^ cells), *Slc29a3*^−/−^ MSCs (5 × 10^5^ cells), alone or combined, to rescue radiation lethality in *Slc29a3*^−/−^ mice after the expression of RFP or ENT3 presented as Kaplan–Meier survival curves, *n* = 6/group, *P < 0.05, **P < 0.01, ***P < 0.001; Mantel-Cox test (**i**) and post transplantion bone marrow cellularity (*n* = 6/group, *P < 0.05; Mantel-Cox test) (**j**). Relative expression of ENT3 in MSCs and HSCs transduced with a lentiviral construct harboring RFP or ENT3 compared with expression in WT mice (**k**). HSCs and MSCs were derived from 8-weeks-old animals (**g**–**k**). Statistical analyses were performed using ANOVA with Tukey’s multiple comparisons post-test and two-tailed Student’s *t-*test. *P < 0.05. Source data are provided as a Source Data file. blue circles, *Slc29a3*^+/+^; magenta squares, *Slc29a3*^−/−^

To understand the nature of ENT3-related dysfunctions, we evaluated the behavior of *Slc29a3*^−/−^ MSCs and HSCs in culture. Primary *Slc29a3*^−/−^ MSCs exhibited rapid exhaustion of clonogenic potential within 2–3 passages (Fig. 3a) with a near-complete loss of MSC-specific marker profiles (Fig. 3b and Supplementary Fig. 3a)^29^. By contrast, *Slc29a3*^−/−^ HSCs exhibited an enhanced proliferative capacity (Fig. 3c). However, the progenies lacking HSC markers (Fig. 3d and Supplementary Fig. 3b) also indicated the loss of HSC clonogenicity. Consistently, *Slc29a3*^−/−^ MSCs and HSCs showed decreased expression of genes (Fig. 3e) essential to maintain multipotentiality^29,30^. In vivo analysis of bone marrow revealed an apparent loss of stem cells beginning at ~12 weeks that reached a classical state of stem cell exhaustion at ~16 weeks (Fig. 3f and Supplementary Fig. 4a). To test whether the lack of ENT3 is a mechanism underlying the exhaustion of bone marrow stem cell populations, we examined the ability of WT and *Slc29a3*^−/−^ HSCs for hematopoietic reconstitution in a uniform C57BL6/J-congenic 129S5/SvEvBrd mouse line. We observed that *Slc29a3*^−/−^ HSCs, and not *Slc29a3*^+/+^ HSCs, failed to reconstitute the bone marrow and thus were unable to rescue the survival of the lethally irradiated mice (Fig. 3g, h). Importantly, when *Slc29a3*^−/−^ HSCs ectopically expressing ENT3 were transplanted into lethally irradiated mice, a dramatic improvement in survival over nontransplanted mice was observed (Fig. 3i–k). In addition, the transplantation of *Slc29a3*^−/−^ MSCs ectopically expressing ENT3 moderately extended survival (Fig. 3i–k). Consistently, combined transplantation of *Slc29a3*^−/−^ HSCs overexpressing ENT3 and *Slc29a3*^−/−^ MSCs overexpressing ENT3 (stem cell transplant; SCT) showed the highest increase in survivability and bone marrow reconstitution (Fig. 3i–k). No survival benefits were observed in mice transplanted with RFP-overexpressing *Slc29a3*^−/−^ MSCs or RFP-overexpressing *Slc29a3*^−/−^ HSCs (Fig. 3i–k). These data further supported the hypothesis that ENT3-dependent stem cell mechanisms are primarily involved in bone marrow exhaustion.

As MSCs and HSCs that jointly share the same microenvironmental niche are both affected in their differentiation functions (Fig. 2a–f), it is possible that the alterations in one cell type are secondary to changes in paracrine factors derived from the other and/or common microenvironmental niche. Therefore, we sought to assess whether the influence of ENT3 on HSCs is altered according to the bone marrow microenvironment to which they are provided access for reconstitution. To eliminate the basal HSCs from *Slc29a3*^*+/+*^ and *Slc29a3*^*−/−*^ bone marrow, we lethally irradiated the mice that were subsequently subjected to HSC transplantation performed using the opposite donor-recipient combination. The failure of hematopoietic reconstitution in irradiated WT mice (that contains *Slc29a3*^+/+^ niche) by *Slc29a3*^−/−^ HSC and rescue of bone marrow failure in irradiated *Slc29a3*^−/−^ mice (that contains *Slc29a3*^−/−^ niche) by *Slc29a3*^+/+^ HSCs suggested that ENT3 affected stem cell functions in a cell-autonomous manner (Supplementary Fig. 4b).

### An autophagy defect underlies stem cell deficits

**Fig. 4:**
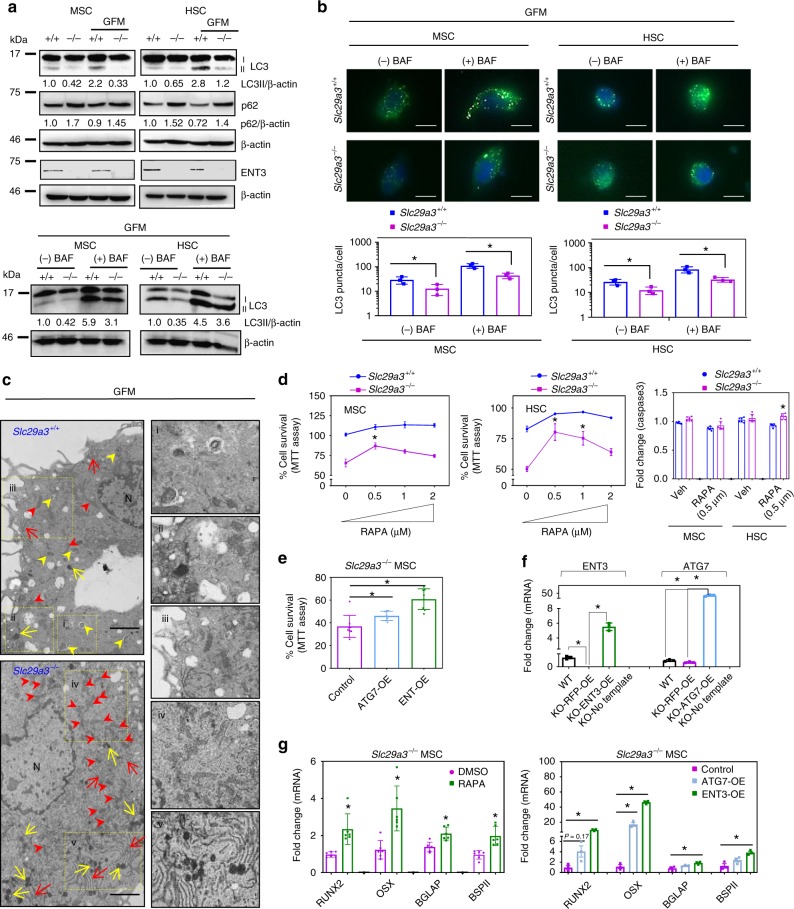
ENT3 loss impairs induction of autophagic response. Representative immunoblots of LC3 and p62 protein expression in MSCs and HSCs under glucose starvation (1 h; above) followed by treatment with vehicle ((−) BAF) or bafilomycin A1 (( + )BAF; 100 nM, 4 h) under glucose starvation (below). β-Actin served as the loading control (**a**). Representative fluorescent images (above) and quantification (below) of LC3 puncta formation (green) in MSCs and HSCs under glucose starvation (1 h) followed by treatment with vehicle ((−) BAF) or bafilomycin A1 (( + )BAF; 100 nM, 4 h) under glucose starvation. Original magnification, × 60; Scale bar: 10 μm. Nuclei stained with DAPI. (*n* = 3, mean ± SEM) (**b**). TEM analysis of autophagosome (yellow arrowhead) and lysosome (yellow arrow), mitochondria (red arrowheads), and ER (red arrow) in *Slc29a3*^+/+^ and *Slc29a3*^−/−^ MSCs. Insets are expanded (right). Scale bar: 2 μm. N, nucleus (**c**). Effect of RAPA (rapamycin) treatment (0.5–2 µM) on GFM-induced MSC and HSC survival (*n* = 4, mean ± SEM) and death (*n* = 6, mean ± SEM) as assayed by MTT assay and active caspase 3 measurement, respectively. The ‘0’’ concentration compares *Slc29a3*^−/−^ cell survival with *Slc29a3*^+/+^ cells and ‘0.5–2’’ µM compares *Slc29a3*^−/−^ cell survival with *Slc29a3*^+/+^ cells in the presence of respective RAPA concentrations. HSCs and MSCs were derived from 12-weeks-old mice (**d**). Effect of ATG7 (ATG7-OE) or ENT3 (ENT3-OE) overexpression on glucose starvation-induced MSC survival (*n* = 8, mean ± SEM) (**e**). Relative expression of ENT3 and ATG7 in *Slc29a3*^−/−^ MSCs and HSCs transduced with lentiviruses harboring RFP or ENT3 compared with expression in *Slc29a3*^+/+^ (WT) mice (*n* = 3, mean ± SEM) (**f**). Effect of RAPA (0.5 µM), ATG7-OE or ENT3-OE on osteogenic medium (3 days)-induced transcription factor and marker gene expression in *Slc29a3*^−/−^ MSCs (*n* = 6, mean ± SEM) (**g**). Statistical analyses were performed using ANOVA with Tukey’s multiple comparisons post-test and two-tailed Student’s *t-*test. *P < 0.05. All phenotypes were assessed in MSCs and HSCs derived from 12-week-old mice. GFM, glucose-free medium. Source data are provided as a Source Data file

As autophagy plays obligate roles in stem cell regulation^31^ and is inextricably linked to lysosomes^32,33^, defective nucleoside transport by ENT3 across lysosomal membranes might impair autophagic regulation of stem cells. Consistent with the hypothesis, basal autophagy was reduced in *Slc29a3*^−/−^ MSCs and HSCs as evidenced by the decreased appearance of the autophagosome marker LC3-II and accumulation of the autophagy substrate Sequestosome1/p62^34,35^ (Fig. 4a). This defect was more evident when cells were deprived of glucose (Fig. 4a, b), indicating that ENT3 regulates both basal and starvation-induced autophagy. The reduction in LC3-II accumulation was more dramatically visualized when autophagic flux was inhibited with bafilomycin A_1_ (an inhibitor of vacuolar-type H^+^-ATPase) under glucose-starved conditions (Fig. 4a, b). TEM analysis revealed fewer autophagosomes and an increased number of lysosomes in *Slc29a3*^−/−^ MSCs compared to MSCs isolated from WT littermate controls (Fig. 4c). In addition, *Slc29a3*^−/−^ MSCs showed an increased presence of mitochondria and ER (Fig. 4c), suggesting a possible lack of autophagic removal of these organelles^36–38^. Following glucose deprivation, loss of ENT3 was associated with compromised cellular viability in both MSCs and HSCs, which could be acutely rescued via activation of the autophagy response with rapamycin^39^, overexpression of the autophagy-related gene ATG7^40^ or reconstitution of the system with an ENT3 expression plasmid (Fig. 4d–f). Caspase activity measurements indicated that the cell death program was minimally involved in the rapamycin-induced recovery of *Slc29a3*^−/−^ cells (Fig. 4d). Moreover, when *Slc29a3*^−/−^ MSCs were induced to differentiate into osteoblasts in the presence of rapamycin or ATG7 overexpression, both the expression of osteoblast transcription factor and marker genes were rescued (Fig. 4g). Taken together, these data suggest that autophagic deficits contribute to stem cell defects in an ENT3-deficient state.

The differentiation of adult stem cells and autophagy are interdependent mechanisms^41^. When stem cells differentiate down a lineage, they reduce their dependence on autophagy, and, conversely, when autophagy is defective, stem cell functions are compromised^42^. We next investigated whether ENT3 could modulate autophagy in a non-stem cell (HEK293) line. Silencing ENT3 (Supplementary Fig. 5a–c) compromised autophagy in HEK293 cells as well, resulting in reduced LC3-II levels (Supplementary Fig. 5d). Importantly, this autophagic regulation appears to be specific to ENT3. Overexpression of ENT3, but not ENT1, and to a lesser extent ENT2, was associated with increased LC3-puncta and increased processing of LC3^35^ (Supplementary Fig. 5e–g). Again, reduced LC-3 II accumulation in ENT3-silenced cells and increased LC-3 II accumulation in ENT3-overexpressing cells were more evident when autophagic flux was inhibited with bafilomycin A1 (Supplementary Fig. 5d, e). To further define the nature of the autophagic regulation by ENT3, we analyzed flux through the pathway using the tandem tagged LC3 (RFP-GFP-LC3) construct. The GFP signal is quenched in the acidic environment of the lysosome, while the red signal is maintained, allowing rapid differentiation of autophagosomes (yellow puncta) and autolysosomes (singly RFP + puncta) by fluorescence^35^. ENT3 increased singly-positive RFP LC-3 puncta fluorescence (Supplementary Fig. 5h, i). When autophagic flux was induced with rapamycin in the presence of chloroquine (to prevent lysosomal degradation), ENT3-silenced HEK293 cells, but not ENT3-reconstituted cells, showed decreased LC3-II (Supplementary Fig. 5j). Altogether, these results identified a direct role for ENT3 in autophagy induction and further supported the hypothesis that ENT3 regulation of stem cell proliferation and differentiation occurs via autophagic modulation.

### ENT3 regulates autophagy via the AMPK-mTOR-ULK pathway

**Fig. 5:**
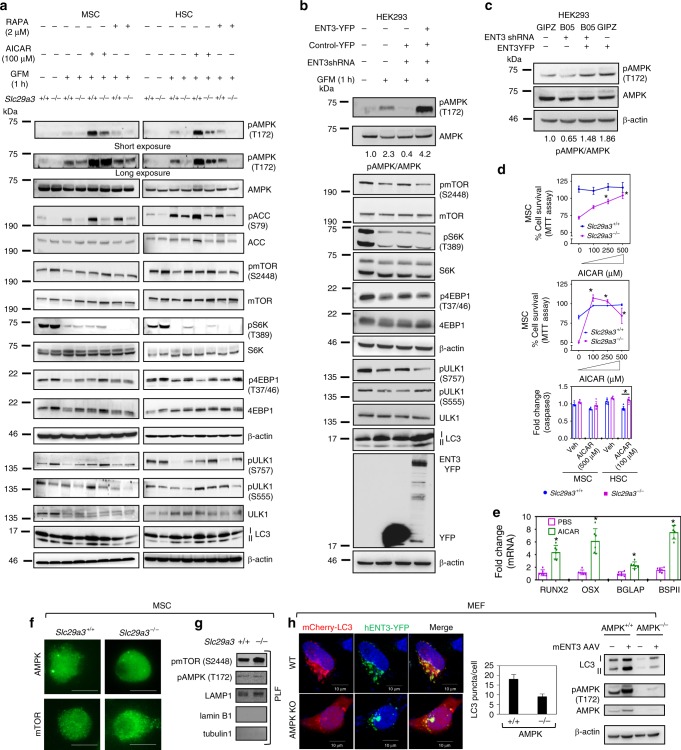
ENT3 activates the AMPK signaling pathway. Representative immunoblots of proteins involved in the AMPK-mTOR-ULK axis in MSCs and HSCs derived from 12-week-old mice under basal, glucose-starved (GFM), and AICAR- or rapamycin (RAPA)-treated conditions (**a**). Immunoblot analysis of the AMPK-mTOR-ULK axis in HEK293-expressing control-shRNA, ENT3-shRNA, pE-YFP, or pEYFP-ENT3 subjected to GFM (**b**). Immunoblotting and quantification of the ENT3 shRNA inhibition of AMPK phosphorylation and reversal by ENT3YFP in HEK293 (**c**). Effect of AICAR on GFM-induced MSC and HSC survival and death as assayed by MTT assay and active caspase 3 measurement, respectively (*n* = 6, mean ± SEM). The ‘0’’ concentration compares *Slc29a3*^−/−^ cell survival with *Slc29a3*^+/+^ cells and ‘100–500’’ µM compares *Slc29a3*^−/−^ cell survival with *Slc29a3*^+/+^ cells in the presence of respective AICAR concentrations (*n* = 4, mean ± SEM). HSCs and MSCs were derived from 12-week-old mice (**d**). Effect of AICAR (500 µM) on osteogenic medium (3 days)-induced transcription factors (TF) and marker genes expression (*n* = 6, mean ± SEM) in *Slc29a3*^−/−^ MSCs. HSCs and MSCs were derived from 12-week-old mice (**e**). Immunostaining analysis of AMPK (green) and mTOR (green) in *Slc29a3*^−/−^ and *Slc29a3*^+/+^ MSCs (derived from 12-week-old mice). Original magnification, × 60; Scale bar: 10 μm (**f**). Immunoblot analysis of pmTOR, pAMPK, LAMP1 (lysosomal marker), lamin B1 (nuclear marker), and tubulin 1 (cytoplasmic marker) in lysates prepared from purified lysosomal fraction (PLF) isolated from *Slc29a3*^−/−^ and *Slc29a3*^+/+^ MSCs (derived from 12-week-old mice) (**g**). WT and AMPK KO (α1/α2^−/−^) MEFs were transfected with mCherry-LC3 and pEYFP-hENT3 plasmids and LC3 and YFP fluorescence were visualized and quantified (*n* = 3, mean ± SEM). Scale bar: 10 μm. Immunoblots of AMPK, pAMPK, and LC3 forms in WT and AMPK KO MEFs transduced with AAV harboring GFP (-) or mENT3 ( + ) (right) (**h**). GFM, glucose-free medium. Statistical analyses were performed using ANOVA with Tukey’s multiple comparisons post-test and two-tailed Student’s *t-*test. *P < 0.05. Source data are provided as a Source Data file

As a high-capacity adenosine transporter^20,22,23^, whether ENT3 influences adenosine monophosphate kinase (AMPK) activity to perturb autophagy has been unexplored. Interestingly, *Slc29a3*^−/−^ MSCs and HSCs showed impaired activation of AMPK (measured via phosphorylation on Thr172 on AMPK) in response to glucose deprivation (Fig. 5a) or AICAR (an inducer of AMPK activity) treatment (Fig. 5a). Reduced phosphorylation of the AMPK target, acetyl CoA carboxylase (ACC)^43^ in *Slc29a3*^−/−^ MSCs and HSCs (Fig. 5a) and a loss of AMPK phosphorylation in ENT3 knockdown HEK293 cells (HEK293KD) (Fig. 5b) further suggested a relationship between ENT3 expression and AMPK activity. Reconstitution of ENT3 in HEK293KD cells resulted in more robust AMPK phosphorylation than observed in parental cells, suggesting an additive effect of ENT3 and glucose deprivation in regulating AMPK phosphorylation (Fig. 5b, c). Intriguingly, when glucose-deprived *Slc29a3*^−/−^ HSCs and MSCs were treated with increasing concentrations of AICAR, there was a near-complete rescue of cellular viability with marked induction of MSC differentiation into osteoblasts (Fig. 5d, e), an effect even more pronounced than that seen with rapamycin (mTOR inhibitor^44^; (Fig. 4d, g)). When investigated for possible AMPK-mTOR interplay, *Slc29a3*^−/−^ MSCs and HSCs, as well as HEK293KD cells, had constitutively high phospho-mTOR (S2448)^45^ that was sustained even under glucose deprivation, AICAR treatment or rapamycin treatment (Fig. 5a, b). Phosphorylation of downstream mTOR targets phospho-S6K (T389)^46^ and phospho-4EBP1 (T37/46),^47^ remained relatively high in *Slc29a3*^−/−^ HSCs, MSCs and HEK293KD cells (Fig. 5a, b). When analyzed for the differential phosphorylation of the autophagy-initiating kinase, ULK1 (Unc-51 like Autophagy Activating Kinase 1)^48^, the levels of phosphoULK1-S757 and phosphoULK1-S555 (the mTOR and AMPK phosphorylation sites, respectively) were reciprocally regulated such that ULK1 phosphorylation at the mTOR site (S757; that inhibits autophagy) was sustained at a higher level while that at the AMPK site (S555; that activates autophagy) was reduced (Fig. 5a, b). Treatment with AICAR marginally restored the levels of ULK1 phosphorylation at S555 in *Slc29a3*^−/−^ MSCs and HSCs although *Slc29a3*^−/−^ MSCs required a higher concentration of AICAR (500 µM) than *Slc29a3*^−/−^ HSCs (100 µM) to produce the same effect (Fig. 5a and Supplementary Fig. 5k). In addition, AICAR induced LC3-II levels in both *Slc29a3*^−/−^ MSCs and HSCs albeit with lesser magnitudes than that observed in WT cells (Fig. 5a, b and Supplementary Fig. 5k). Other AMPK activators such as metformin and azacytidine also moderately induced LC3-II levels in *Slc29a3*^−/−^ MSCs (Supplementary Fig. 5l). Furthermore, ENT3 expression synergistically enhanced the effects of the mTOR inhibitor, Torin1, to induce autophagy (Supplementary Fig. 5m). In addition to glucose stimulation, amino acid stimulation, to a lesser degree, increased mTOR phosphorylation, suggesting that mTORC1 regulation by ENT3, might not be exclusively due to AMPK activity (Supplementary Fig. 6a).

As both AMPK and mTOR have been shown to be regulated on the lysosome^46,48^, we next interrogated whether the subcellular distribution of these kinases differed in *Slc29a3*^−/−^ MSCs. While AMPK displayed a diffuse, cytosolic staining pattern with the occasional presence of large dotted structures, mTOR showed a predominantly vesicular staining pattern throughout the cytoplasm (Fig. 5f). However, unlike the reduced phospho-AMPK levels observed in total cell lysates prepared from *Slc29a3*^−/−^ MSCs (Fig. 5a), the phospho-AMPK levels were not reduced in the lysosomal fractions isolated from *Slc29a3*^−/−^ MSCs (Fig. 5g). These results suggested that the AMPK regulatory events predominantly occurred in the non-lysosomal (i.e., cytosolic) compartments of *Slc29a3*^−/−^ MSCs. On the other hand, the phospho-mTOR levels (S2448) were relatively higher in the lysosomal fractions isolated from *Slc29a3*^−/−^ MSCs (Fig. 5g), suggesting that mTOR regulation, at least in part, occurred on the lysosome. These results were also consistent with the increased proliferation of lysosomes observed in *Slc29a3*^−/−^ MSCs (Fig. 4c). Exogenous ENT3 expression in AMPK α1/α2 double KO mouse embryo fibroblasts (MEFs)^49^ failed to generate an adequate autophagic response with reduced LC3 II formation, which corroborated an integral role for AMPK signaling in eliciting ENT3’s autophagic responses (Fig. 5h).

**Fig. 6:**
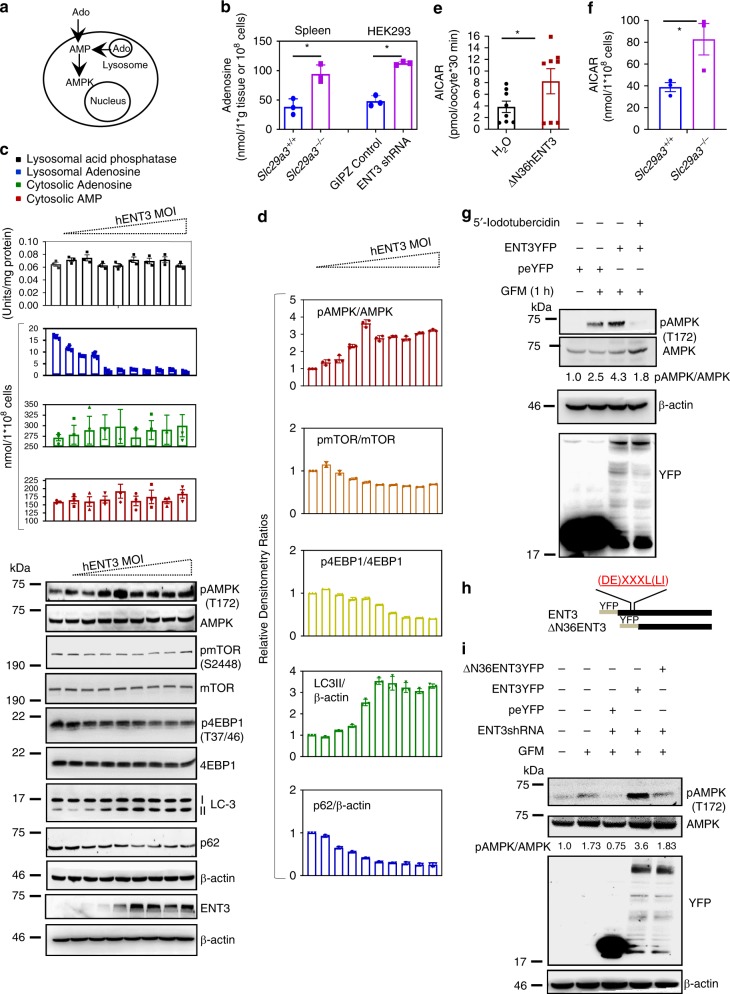
ENT3 lysosomal adenosine transport regulates AMPK and autophagy signaling. Schematic representation of the activation of AMPK by extracellular and lysosomal pools of adenosine; Ado, adenosine; AMP, adenosine monophosphate (**a**). Mass spectrometry analysis of adenosine levels in lysosomes derived from mouse spleen lysates and HEK293 cells expressing GIPZ-shRNA (control) or ENT3 shRNA (*n* = 3, mean ± SEM) (**b**). HEK293 cells transduced with increasing MOI of hENT3 retroviruses were analyzed for lysosomal adenosine and cytosolic adenosine and AMP, and lysosomal acid phosphatase activity (*n* = 3, mean ± SEM) (**c**; above). Whole-cell lysates prepared from cells infected with the corresponding MOIs of hENT3 viruses were probed with the indicated antibodies. β-Actin served as the loading control (**c**; below). Quantification results from densitometric analysis of the immunoblots (*n* = 3, mean ± SEM) (**d**). Uptake of AICAR (20 μm) into Xenopus oocytes at 25 °C after 22 h of injection of N-terminal deleted ENT3 transcripts (ΔN36ENT3; to remove the lysosomal-targeting signal (shown in (**h**)) and enabling cell surface localization) was measured in transport buffer with pH 5.5. AICAR concentrations measured by mass spectrometry (*n* = 8 oocytes, mean ± SEM) (**e**). Mass spectrometry analysis of AICAR levels in lysosomes derived from MSCs derived from 12-week-old mice after the treatment of MSCs with AICAR (20 μm) for 4 h (*n* = 3, mean ± SEM) (**f**). HEK293 cells cultured in GFM were transfected with pE-YFP or pE-ENT3-YFP and treated with iodotubericidin (50 nM) for 4 h and lysates examined for indicated proteins. Iodotubericidin abrogates glucose starvation and ENT3-induced pAMPK activation. GFM, glucose-free medium (**g**). Schematic representation of WT ENT3 (ENT3) and localization-deficient ENT3 (ΔN36ENT3) mutants fused with YFP. A lysosomal-targeting motif in the N-terminus of ENT3 (red) and YFP tag (yellow) at the N-terminus are shown (**h**). HEK293 cells transduced with WT ENT3 but not N-terminal deleted ENT3 elicit glucose starvation-induced pAMPK activation (**i**). Statistical analyses were performed using two-tailed Student’s *t-*test. *P < 0.05. Source data are provided as a Source Data file

As ENT3 is an acidic pH-activated transporter^19,22,23^ and the acidic milieu of eukaryotic lysosomes can generate high levels of adenosine through the degradation of nucleic acids arising from autophagic (or phagocytic) pathways, we next examined whether ENT3-mediated translocation of a lysosomal pool of adenosine into cytosol activates AMPK signaling (Fig. 6a). Supportively, cell fractionation and mass spectrometry analysis of lysosomal fractions of *Slc29a3*^−/−^ spleen (derived from mesodermal mesenchyme and harbors HSCs) and HEK293KD cells demonstrated the increased accumulation of adenosine (Fig. 6b and Supplementary Fig. 6). These findings indicated that reduced lysosomal recycling of adenosine negatively influences AMPK signaling in *Slc29a3*^−/−^ mice. To test directly, we tracked the efflux of adenosine from lysosomes and measured the changes in cytosolic adenosine, AMP and AMPK phosphorylation after the transduction of HEK293KD with increasing multiplicity of infection (MOI) of hENT3-harboring retroviruses. An increase in the lysosomal efflux of adenosine with parallel increases in AMP and AMPK phosphorylation levels and LC3 lipidation as well as a decrease in AMPK downstream effectors (pmTOR) was observed in a saturable manner with a graded increase in ENT3 expression (Fig. 6c, d). In addition to the accumulation of endogenously generated adenosine in lysosomes, a relatively higher accumulation of AICAR (an adenosine analog transported by ENT3 (Fig. 6e)) was also observed in the lysosomal fractions isolated from AICAR-treated *Slc29a3*^−/−^ MSCs (Fig. 6f), partly explaining the attenuated response of AICAR to activate AMPK in *Slc29a3*^−/−^ MSCs (Fig. 5a). Moreover, the activation of AMPK by adenosine was dependent on its conversion to AMP because the inhibition of adenosine kinase by iodotubericidin completely abolished ENT3-induced AMPK phosphorylation (Fig. 6g). Furthermore, the capacity of ENT3 to increase AMPK phosphorylation was dependent on its lysosomal localization because the overexpression of the N-terminal truncated mutant (∆N36-ENT3-YFP) that removes the lysosomal-targeting signal from ENT3^20^ failed to induce AMPK phosphorylation in HEK293KD cells (Fig. 6h, i).

### ENT3 loss alters fatty acid utilization and bioenergetics

**Fig. 7:**
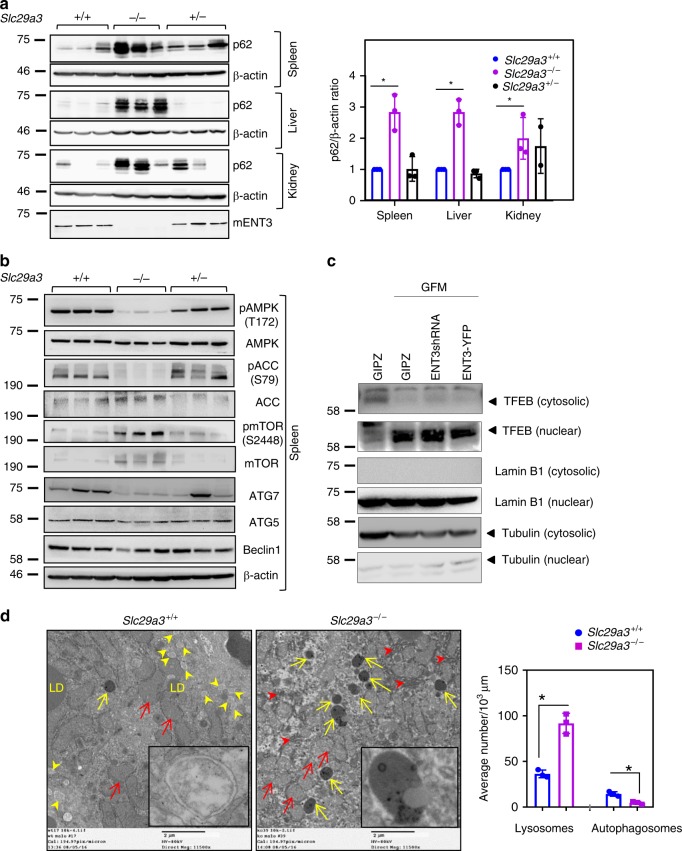
Autophagy perturbation in *Slc29a3*^−/−^ adult mouse tissues. Immunoblotting analysis (left) and quantification (right) of lysates prepared from the *Slc29a3*^−/−^ spleen, liver, and kidney showing the accumulation of p62 compared with that in *Slc29a3*^+/+^ tissues (**a**). Immunoblotting analysis of the AMPK-mTOR-ULK axis and core autophagy genes in *Slc29a3*^+/+^, *Slc29a3*^−/−^ and *Slc29a3*^+/−^ splenic lysates (**b**). HEK293 cells were transfected with control or hENT3YFP plasmids, and the cytosolic and nuclear fractions were isolated and examined for alterations in the TFEB levels using immunoblotting analysis. No significant changes in the TFEB levels were identified in the presence or absence of hENT3. Lamin B1 was used as a nuclear marker, and tubulin was used as a cytosolic marker. β-Actin served as the loading control (**c**). TEM analysis (left) and quantification (right; (*n* = 3, mean ± SEM)) of lysosomes (yellow arrow) and autophagosomes (yellow arrowhead) in *Slc29a3*^+/+^ and *Slc29a3*^−/−^ mouse livers. Insets show representative lysosome and autophagosome structure(s) in *Slc29a3*^+/+^ and *Slc29a3*^−/−^ livers, respectively. Increased proliferation of mitochondria (red arrow) and ER (red arrowhead) in *Slc29a3*^−/−^ livers. LD, lipid droplets; Scale bars: 2 μm (**d**). Statistical analyses were performed using two-tailed Student’s *t-*test. *P < 0.05. Source data are provided as a Source Data file

**Fig. 8:**
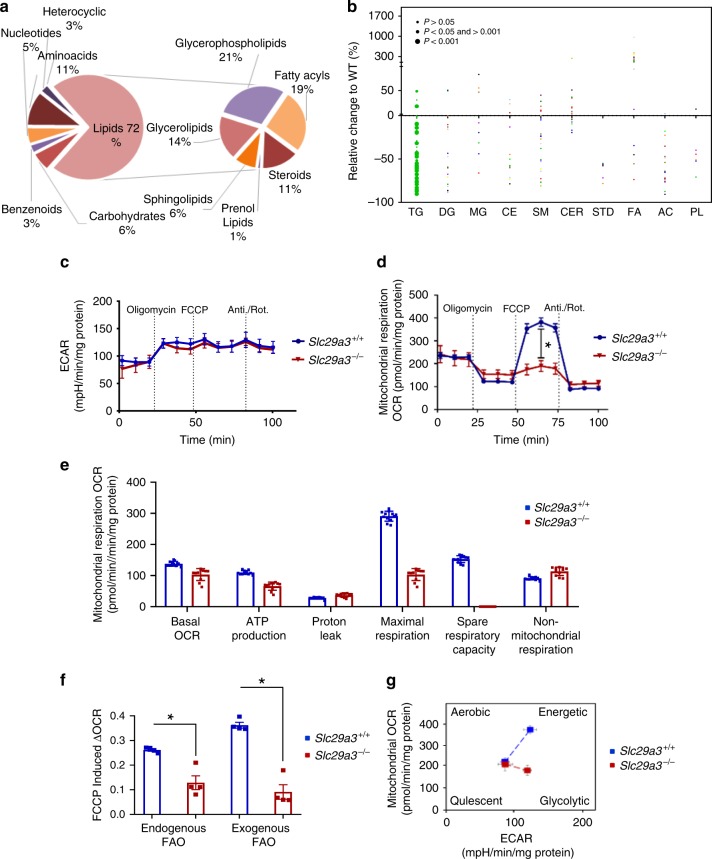
Disrupted lipid homeostasis and altered fuel source selection in ENT3 deficiency. Pie-of-pie chart representation of total metabolites and those within the lipid superclass in the *Slc29a3*^−/−^ liver (**a**). Bubble chart representation of quantified lipids by targeted lipidomics (shown in Supplementary Fig. 9). Bubble, individual lipid; bubble size, significance (inset); *n* = 12 (**b**). Real-time measurements of the extracellular acidification rate (ECAR) (**c**) and oxygen consumption rate (OCR) (**d**) in *Slc29a3*^−/−^ MSCs (*n* = 11, mean ± SEM). In addition to the basal levels, the fraction of OCR and ECAR contributing to ATP-linked OCR/ECAR measured by sequential injection of oligomycin, FCCP and antimycin/rotenone (Anti/Rot) are presented (**c**, **d**). Mitochondrial substrate utilization parameters in *Slc29a3*^−/−^ MSCs derived from **c** and **d** (**e**). Quantification of the oxidation of endogenous and exogenous palmitic acid (*n* = 4, *Slc29a3*^+/+^ MSCs; *n* = 6, *Slc29a3*^−/−^ MSCs, mean ± SEM). FAO, fatty acid oxidation (**f**). Bioenergetics phenotype derived from the MitoStress test (**g**). Statistical analyses were performed using two-tailed Student’s *t-*test. *P < 0.05. Source data are provided as a Source Data file

In the next set of experiments, we tested whether the autophagy defect observed in cultured MSCs and HSCs was also evident in mouse tissues. Examination of several organs from adult *Slc29a3*^−/−^ mice demonstrated the increased accumulation of p62 (Fig. 7a). In addition, *Slc29a3*^−/−^ spleens showed alterations in AMPK and downstream targets (Fig. 7b). Reduced levels of ATG7 (Fig. 7b), but not transcription factor EB (TFEB) (Fig. 7c), further suggested the involvement of the core autophagic machinery but not lysosome-to-nucleus signaling^32,35^. Consistently, TEM analysis of *Slc29a3*^−/−^ mouse livers demonstrated fewer autophagosomes and more lysosomes, mitochondria, and ER (Fig. 7d). Intriguingly, *Slc29a3*^−/−^ livers displayed fewer and smaller lipid droplets (Supplementary Fig. 7a). In addition, BODIPY 493/503 staining of neutral lipid droplets was lower in oleic acid-treated *Slc29a3*^−/−^ hepatocytes, indicating defects in the formation of lipid droplets (Supplementary Fig. 7b). These observations, along with the perturbation in the major energy and nutrient-sensing pathways (AMPK and mTOR), suggested potential metabolic alterations additionally driving ENT3-related dysfunction in mice. As the formation of neutral lipid droplets largely relies on glucose and patients with PHID syndrome exhibit insulin-dependent diabetes mellitus^6^, we tested whether *Slc29a3*^−/−^ mice display an abnormal glycemic profile. Although the intraperitoneal glucose tolerance test revealed a mildly elevated glycemic response in *Slc29a3*^−/−^ mice, the extent of glycemia was minimal (Supplementary Fig. 7c). Therefore, to gain a better understanding of the metabolic dysregulation in *Slc29a3*^−/−^ cells, we performed mass spectrometry-based untargeted metabolomic profiling of the *Slc29a3*^−/−^ mouse liver. As stem cells (particularly HSCs) were sample limiting for in-depth metabolomic analyses, we chose liver tissue for this analysis primarily because of its high responsiveness to autophagy and its role in regulating systemic metabolic homeostasis. Additionally, the liver has the highest ENT3 expression among all mouse tissues (http://symatlas.gnf.org). This analysis detected 8410 total metabolites in the *Slc29a3*^−/−^ liver, among which 879 metabolites were significantly altered (*P* < 0.05 and fold change > 2.0) (Fig. 8a, Supplementary Fig. 7d, and Supplementary Data 1). A subsequent VIP plot and S-plot generated from supervised PLS-DA and OPLS-DA analysis, respectively, and mass spectral confirmation of second-order MS fragmentation patterns identified lipids as the most affected superclass (95 out of 175; Fig. 8a and Supplementary Fig. 7e, f and 8). To delineate alterations within the lipid superclass, we subsequently conducted mass spectrometry-based targeted lipidomics. This analysis revealed that 120 of 498 individual lipids examined were significantly altered (*P* < 0.05 and fold change > 2.0) (Fig. 8b, Supplementary Fig. 9, and Supplementary Data 2). Remarkably, highly elevated polyunsaturated and monounsaturated free fatty acids and generalized decreases in neutral lipids (fatty acylglycerol esters (changes in triglycerides > diglycerides > monoglycerides) and cholesteryl esters), prenol lipids, glycerophospholipids, and steroids were identified as striking features (Fig. 8b and Supplementary Fig. 9). Consistently, *Slc29a3*^−/−^ mouse plasma displayed severe dyslipidemic features, including hypocholesterolemia, hypotriglyceridemia, and hypolipoproteinemia (Supplementary Fig. 10a).

As metabolic plasticity is a critical requirement for stem cell lineage specification, we next investigated whether metabolic reprogramming also occurred in *Slc29a3*^−/−^ MSCs. Although the extracellular acidification rate (a measure of glycolysis) was unaltered (Fig. 8c), a Seahorse XF96 cell Mito test demonstrated that the mitochondrial oxygen consumption rate was reduced in *Slc29a3*^−/−^ MSCs under bioenergetics stress (Fig. 8d). In addition, decreased maximal respiration and ATP production, increased non-mitochondrial respiration and proton leak, and a loss of the spare reserve capacity were identified in *Slc29a3*^−/−^ MSCs (Fig. 8c–e). Importantly, when measured for endogenous and exogenous palmitic acid oxidation, simultaneous reductions in mitochondrial β-oxidation capacities were identified from both sources (Fig. 8f). Furthermore, targeted mass spectrometry analysis of select lipid metabolites recapitulated the elevated presence of several free fatty acids and the decreased presence of diglycerides, triglycerides, phosphatidylcholine, and phosphatidylethanolamine in *Slc29a3*^−/−^ MSCs (Supplementary Fig. 10b) similar to that observed in the mouse liver. Compared with WT MSCs, *Slc29a3*^−/−^ MSCs displayed a relatively quiescent and glycolytic phenotype (Fig. 8g), indicating metabolic reprogramming and alterations in fuel source selection. Treatment of *Slc29a3*^−/−^ MSCs with AICAR partially normalized lipid metabolite alterations with the restoration of several metabolites to levels comparable to those of WT (Supplementary Fig. 10b). As AMPK also restored proliferation and differentiation defects in *Slc29a3*^−/−^ MSCs (Fig. 5d, e), taken together, these findings suggested mitochondrial bioenergetics alterations can additionally propel stem cell deficits in *Slc29a3*^−/−^ mice.

### Rescue of ENT3-related dysfunctions in KO mice

**Fig. 9:**
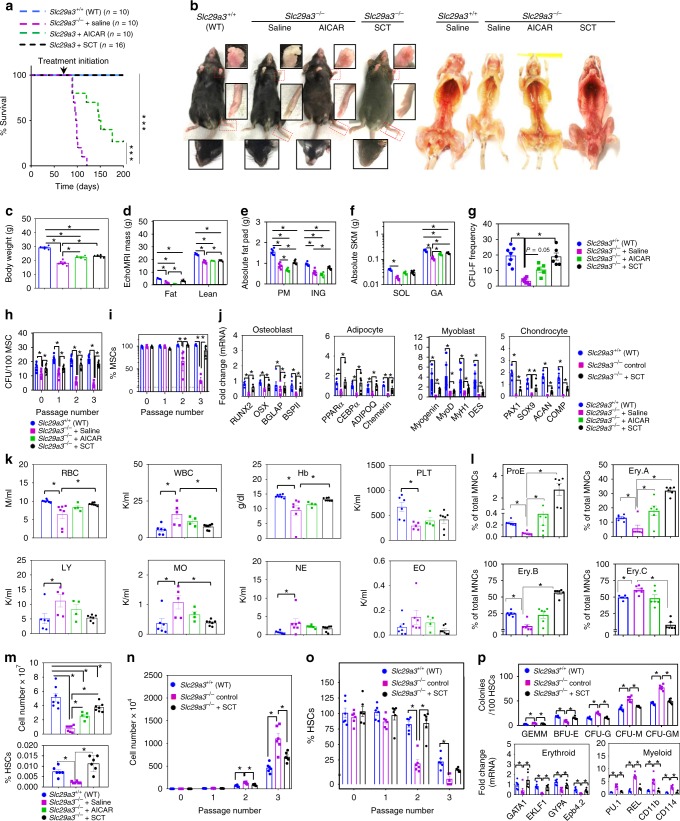
AICAR and SCT improve survival and alleviate dysfunction in *Slc29a3*^−/−^ mice. AICAR injection (500 mg/kg; SID) (*n* = 10/group) and SCT (1 × 10^4^ *Slc29a3*^+/+^ HSCs and 5 × 10^5^ MSCs) (*n* = 16/group) extend the survival of *Slc29a3*^−/−^ mice (***P < 0.001; Mantel-Cox test) (**a**). AICAR-treated surviving mice show facial alopecia (insets; below), while both the AICAR and SCT groups show improved appearance and medullary hematopoiesis (right) (**b**). Changes in body weight (**c**), EchoMRI-measured fat and lean mass (**d**), absolute parametrial (PM) and inguinal (ING) fat pad mass (**e**), absolute soleus (SOL) and gastrocnemius (GA) skeletal muscle (SKM) mass (**f**) and bone marrow CFU-F frequency (**g**) in AICAR-treated surviving mice and SCT mice (*n* = 6, mean ± SEM). Clonogenicity (**h**) and marker expression (**i**) with serial passage of MSCs derived from SCT mice (*n* = 6, mean ± SEM). mRNA expression of transcription factors and markers after 14 (osteoblasts and adipocytes) and 28 (myocytes and chondrocytes) days of the differentiation of MSCs derived from SCT mice (*n* = 6, mean ± SEM) (**j**). Hematological parameters in AICAR-treated surviving mice and SCT mice. AICAR-treated surviving *Slc29a3*^−/−^ mice (*n* = 4, mean ± SEM) and SCT mice (*n* = 7, mean ± SEM) at 28 weeks are compared with saline-treated *Slc29a3*^−/−^ (*n* = 5, mean ± SEM) and WT mice (*n* = 6, mean ± SEM) at 12 weeks (**k**). Frequency of erythroid subpopulations within the bone marrow of AICAR-treated surviving mice and SCT mice (*n* = 6, mean ± SEM) (**l**). Cellularity (above) and HSC frequencies (below) in AICAR-treated (*n* = 5, mean ± SEM) and SCT mouse bone marrow (*n* = 7, mean ± SEM) (**m**). Culture expansion capacity (**n**) and percent of cells expressing HSC markers (**o**) with serial passage of SCT mouse bone marrow-derived HSCs (*n* = 6, mean ± SEM). CFU-forming capacity (above) and mRNA expression of transcription factors and markers (below) after 14 days of HSC differentiation in SCT mice (*n* = 6, mean ± SEM) (**p**). SCT, stem cell transplantation; CFU, colony-forming unit; CFU-F, CFU-Fibroblast; Hb, hemoglobin; PLT, platelet; LY, lymphocyte; MO, monocyte; NE, neutrophil; EO, eosinophil; ProE, proerythroblasts; EryA, early basophilic erythroblasts; EryB, late basophilic and polychromatic erythroblasts; EryC, orthochromatic erythroblasts/reticulocytes; GEMM, granulocyte, erythrocyte, monocyte, megakaryocyte; BFU-E, burst-forming unit-erythroid, colony-forming unit, CFU; CFU-M, CFU-macrophage, CFU-G, CFU-granulocyte; CFU-GM, CFU-granulocyte, macrophage. Statistical analyses were performed using ANOVA with Tukey’s multiple comparisons post-test and two-tailed Student’s *t*-test. *P < 0.05. Source data are provided as a Source Data file

Finally, we evaluated whether stem cell transplantation (SCT, which increases short-term survival in lethally irradiated mice (Fig. 3i)) or in vivo AMPK activation (AICAR treatment) can reduce ENT3 disease pathologies and increase the long-term survival of *Slc29a3*^−/−^ mice. We injected 1 × 10^4^ *Slc29a3*^+/+^ HSCs and 5 × 10^5^ MSCs (once) intravenously into 10-week-old asymptomatic *Slc29a3*^−/−^ mice (SCT) or AICAR at 50 mg/kg (daily) by intraperitoneal delivery beginning at 10 weeks of age (Fig. 9a). While saline-treated *Slc29a3*^−/−^ mice showed a rapid decline in survival as expected, the chronic administration of AICAR significantly prevented morbidity and improved the overall survival in *Slc29a3*^−/−^ mice with ~50% of mice surviving beyond 22 weeks (Fig. 9a). Despite the patchy alopecia observed in facial regions particularly near the muzzle and surrounding the eyes (Fig. 9b), the AICAR-treated surviving mice at 28 weeks of age (after ~10 weeks of death of saline-treated *Slc29a3*^−/−^ mice) showed overall improvement in health, body weight and hematopoiesis compared with *Slc29a3*^−/−^ mice at 12 weeks of age (Fig. 9a–c). Strikingly, SCT mice showed 100% survival at 28 weeks of age with notable improvement in health, body weight and hematopoiesis compared with *Slc29a3*^−/−^ mice at 12 weeks of age (Fig. 9a–c). EchoMRI and necropsy revealed in both AICAR-treated and SCT mice with preserved muscle mass, but only SCT mice showed preserved adipose tissue mass (Fig. 9d–f). When the capacity of bone marrow MSCs were analyzed, MSCs derived from SCT mice, but not AICAR-treated mice, fully restored the colony-forming unit-fibroblast (CFU-F) to WT levels (Fig. 9g). To further confirm whether the CFU-F-derived progenies were truly MSCs, we assessed MSC surface marker expression by flow cytometry. The percentage of MSCs within the CFU-F at each passage was markedly higher in the SCT group (than KO group), confirming the recovery of MSC self-renewal in addition to the gain in CFU-F formation in the SCT group (Fig. 9h, i and Supplementary Fig. 11a). Furthermore, the MSC differentiation abilities in SCT mice were substantially rescued as assessed by the levels of lineage-specific markers and transcription factors (Fig. 9j).

Consistent with the gross observations in medullary regions showing improved hematopoiesis in the AICAR-treated and SCT groups (Fig. 9b), blood analysis revealed a trend of an increase in RBCs, the hemoglobin content and the platelet count with a decrease in the granulocytic and agranulocytic WBC counts (Fig. 9k). Compared with the AICAR-treated group, the SCT group showed a superior rescue effect for many parameters (Fig. 9k). When bone marrow erythroid subpopulations were further characterized, we observed a reversal of the changes in the proportion of cells at various erythroblast maturation stages in the SCT group compared with that in *Slc29a3*^−/−^ mice at 12 weeks of age (Fig. 9l and Supplementary Fig. 11b). The reduced proportion of immature stages (proerythroblasts (ProE), basophilic erythroblasts (EryA), late basophilic erythroblasts (EryB)) observed in *Slc29a3*^−/−^ mice at 12 weeks of age was increased in the SCT group to levels beyond that seen in the WT group, suggesting the expansion of erythroid precursors in the SCT group (Fig. 9l). This shift towards an immature population resulted in a percent decrease in the mature orthochromatic/reticulocyte erythroblast (EryC) in the SCT group (Fig. 9l). However, the findings regarding the erythroid frequencies with increased bone marrow cellularity (and % HSCs in SCT group) (Fig. 9m) suggested that the absolute numbers of HSCs and immature cell types (ProE to EryB) are dramatically increased while the number of mature erythroblasts (EryC) is still maintained in the SCT group (relative to *Slc29a3*^−/−^ mice at 12 weeks of age). The increase in the percentage of HSCs further suggested that the bone marrow regeneration might have resulted from the continuous proliferation and self-renewal of the primitive HSCs in the SCT group (Fig. 9m). To examine this directly, we assessed the changes in the HSC number and expression of surface markers during in vitro proliferation with serial passage. Abnormal proliferation with the loss of markers in *Slc29a3*^−/−^ HSCs was normalized in the SCT group, suggesting a recovery of self-renewal (Fig. 9n, o and Supplementary Fig. 11c). Similarly, the abnormal increase in myeloid subsets (CFU-G, CFU-M, and CFU-GM) with the loss of BFU-E was normalized in the SCT group with corresponding changes in erythroid and myeloid transcription factors and markers (Fig. 9p). In addition to SCT, AICAR treatment moderately increased erythroid (ProE, EryA, and EryB) frequencies (Fig. 9l and Supplementary Fig. 11b) and bone marrow cellularity (Fig. 9m), however, sample limitation from AICAR-treated surviving mice at the end of the treatment period precluded us from conducting many detailed studies.

To study the effect of AICAR on freshly isolated stem cells, we treated another batch of *Slc29a3*^−/−^ mice with saline or AICAR (daily; 50 mg/kg; intraperitoneal) for a shorter period (6 weeks, starting at 10 weeks) and examined for changes in HSC and MSC pools. Consistent with the results on continuous administration of AICAR on *Slc29a3*^−/−^ mice survival (for 28 weeks), even the AICAR-treated younger *Slc29a3*^−/−^ mice (at 16 weeks) showed improvement in the number of HSCs and MSCs (Supplementary Fig. 12a–d). Further, lysates prepared from the bone marrow and spleens isolated from the AICAR-treated mice at 16 weeks showed increased LC3-II levels (Supplementary Fig. 12e). These results provided further support that the AICAR induced increases in HSC and MSC abilities contribute to the partially reduced ENT3 disease pathologies and improved survival of *Slc29a3*^−/−^ mice.

## Discussion

**Fig. 10:**
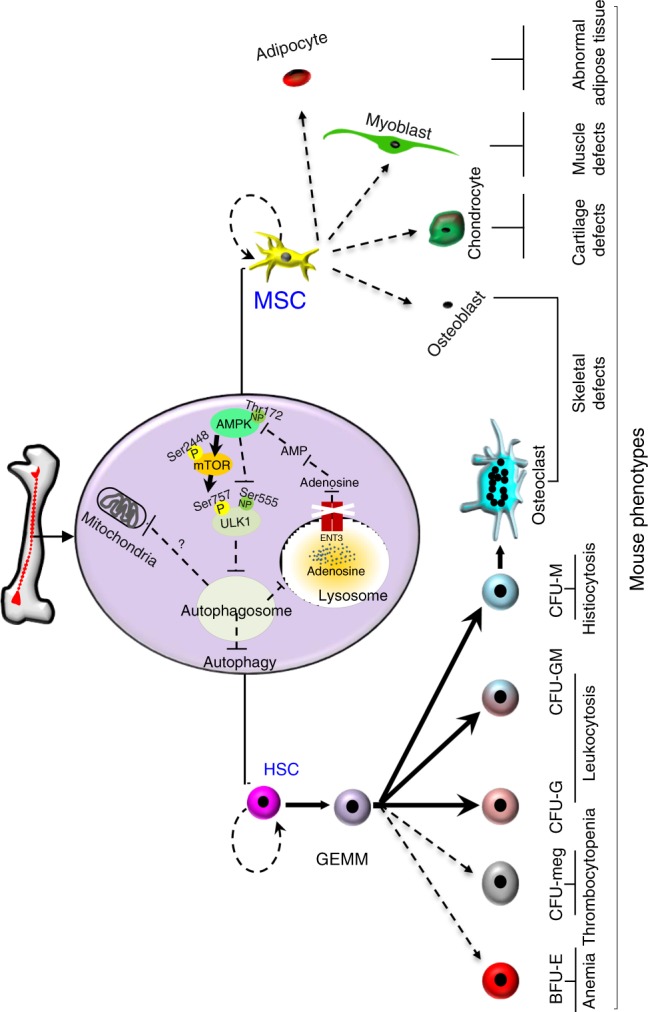
Schematic representation of disease pathogenesis in *Slc29a3*^−/−^ mice. Molecular, cellular and tissue defects in *Slc29a3*^−/−^ mice are depicted. Broken lines or arrows indicate inhibitory pathways, and thick arrows indicate the stimulatory pathways occurring in *Slc29a3*^−/−^ mice. Phosphorylation statuses of AMPK, mTOR, and ULK in *Slc29a3*^−/−^ cells are shown (P, phosphorylated, yellow circle; NP, non-phosphorylated, green circle). CFU, colony-forming unit; E, erythroid; meg, megakaryocyte; G, granulocyte; M, macrophage; GM, granulocyte/macrophage; GEMM, granulocyte/erythrocyte/macrophage/megakaryocyte

It is increasingly evident that mutations in the ENT3 gene are solely responsible for a growing spectrum of human genetic disorders^5–16^. However, due in part to the complexity and heterogeneity of ENT3-related dysfunctions, the origin of dysfunction and molecular mechanism(s) by which ENT3 regulates cellular and tissue homeostasis have remained obscure. Here, we first established a role for ENT3 in the self-renewal and differentiation capabilities of multipotent MSCs and HSCs. Second, we identified that ENT3 mobilizes lysosomal adenosine pool to regulate AMPK-mTOR-ULK signaling, the disruption of which leads to widespread autophagic insufficiency. Third, we found that the impairment of differentiation capabilities of HSCs and MSCs in ENT3-null mice was associated with stem cell exhaustion and breaches of mesodermal tissue integrity. Finally, stem cell transplantation demonstrated the dramatic rescue of tissue pathologies and survivability in ENT3-deleted mice, whereas the pharmacological activation of AMPK by AICAR produced a partial recovery. Altogether, these discoveries identified the involvement of an intracellular nucleoside transporter in adult stem cell homeostasis. Additionally, the data provide a mechanistic explanation for the complex and heterogeneous tissue pathologies observed in ENT3-related disorders (Fig. 10). Moreover, the findings support an intriguing possibility that ENT3 senses the lysosomal adenosine levels and determines the activation levels of the central energy sensor (AMPK), which, in turn, relays the information to core autophagic machinery to ensure normal tissue and organismal development (Fig. 10).

Our study provides the evidence for the impairment of self-renewal and differentiation functions in ENT3-deficient MSCs and HSCs through perturbation of the transport of adenosine across lysosomal membranes. As *Slc29a3*^−/−^ mice largely phenocopy the abnormalities reported in autophagy-deficient models^31,37,50,51^ and autophagic induction via AMPK activation partially ameliorates dysfunctions, our findings support that the loss of autophagic signaling as a probable cause of stem cell dysfunction in ENT3 KO mice. Overall, the evidence support that the autophagy inducing effects of AMPK pathway to contribute to the survival of ENT3-deficient cells although additional contributions from the autophagic-independent effects of this multifunctional kinase cannot be completely negated. The preponderance of dysfunction in mesoderm-derived tissues demonstrates that the defects originated at the lower hierarchical MSCs and HSCs and not the higher hierarchical cells, a phenomenon that would be expected to produce abnormalities in all three germ layers. This dysfunction also suggests that MSCs and HSCs exhibit a higher dependency on ENT3-regulated autophagic needs to sustain mesodermal tissue integrity. Our study also uncovered that ENT3 mobilizes the intracellular pool of adenosine from the lysosomes to increase AMPK phosphorylation, a process that directly activates autophagy. The constitutively active mTOR activity downstream to AMPK loss also indicates a higher energy state in *Slc29a3*^−/−^ cells that supports cell survival mechanisms but compromises the ability to adapt to higher energy demands via autophagy. Thus, the present findings indicate ENT3 intricately regulates autophagy by involving both AMPK and mTOR signaling pathways.

In summary, by evaluating the mutational consequences of hENT3 disorders in ENT3-deficient mice, we investigated hallmarks of hENT3 pathophysiologies and evaluated therapeutic interventions. Forced activation of AMPK by AICAR partially ameliorates the severity of ENT3-related dysfunction, whereas stem cell transplantation appears even more promising with dramatic survival benefits in mice. Altogether, these findings have important implications to understand ENT3 pathophysiology and to treat humans with ENT3 disorders.

## Methods

### Cells, plasmids, reagents, and antibodies

Retroviral (ATCC, CRL 9078) and lentiviral packaging (ATCC, CRL 11268) cell lines and HEK 293 cells (ATCC, CRL 1573) were purchased from American Type Culture Collection (ATCC, Manassas, VA, USA). AMPK α1/α2 double-knockout MEFs were a kind gift from Dr. B. Viollet (Institut Cochin INSERM U1016, CNRS UMR 8104, Université Paris Descartes, Department of Endocrinology, Metabolism and Cancer, Paris, France. Each cell line was tested negative for mycoplasma contamination (MycoAlertTM Mycoplasma Detection kit, Lonza).

pEYFP-hENT3, pOX-ΔN36hENT3, LNCX2-ΔN36hENT3, and mRFP-EGFP-LC3 plasmid constructs were described previously^18,20^. Human *SLC29A1* and h*SLC29A2* were cloned into pEYFP-C1 (Clontech, Mountain View, CA) and were designated as pEYFP-hENT1 and pEYFP-hENT2, respectively. The pcDNA3.1-mCherry-hLC3B plasmid (40827) was obtained from Addgene^52^. The GIPZ lentiviral vector harboring shRNA sequences targeting *SLC29A3* (RHS4531-EG55315) and a non-targeting control-shRNA were obtained from Dharmacon, Chicago, IL. The pAAV-mSlc29a3 (Cat. AAV0925011), pAAV-CMV-GFP-control (Cat. AAV1000), pLenti-GIII-CMV-ATG7 (Cat. LV082738) and pLenti-GIII-Slc29a3-Puro (Cat. LV451098) plasmid constructs were obtained from Applied Biological Materials Inc. (Richmond, BC, Canada).

All chemicals were procured from Sigma (St. Louis, MO) unless otherwise indicated. Rapamycin was purchased from Enzo Life Sciences, and Torin1 was purchased from Sellekchem (Houston, TX). The BCA protein assay kit was purchased from Thermo Scientific, the lentiviral packaging kit was purchased from Dharmacon (Chicago, IL, USA) and the adeno-associated viral packaging kit was purchased from Applied Biological Materials Inc. (Richmond, BC, Canada). Fetal bovine serum and horse serum were from HyClone Laboratories (Logan, UT). Dialyzed fetal bovine serum was from Sigma (St. Louis, MO). Fluorescent Antifade mounting reagent and penicillin–streptomycin were obtained from Molecular Probes (Eugene, OR). High-glucose DMEM and no-glucose DMEM cell culture media were obtained from Thermo Scientific (Waltham, MA).

Goat polyclonal antibodies against carboxyl (C20; sc-48147; 1:1000 dilution) or amino (N18; sc-48149; 1:1000 dilution) terminus of hENT3 were obtained from Santa Cruz Biotechnology (Santa Cruz, CA), and the rabbit polyclonal antibody generated against the third intracellular loop of hENT3 was described earlier^20^. The rabbit polyclonal anti-hENT3 antibody (PA5-38039; 1:1000 dilution) was purchased from Thermo Scientific (Waltham, MA). Rabbit monoclonal antibodies against LC3B (#3868; 1:1000 dilution), pmTOR (#5536; 1:1000 dilution), mTOR (#2983; 1:1000 dilution), p4EBP1 (#2855; 1:1000 dilution), 4EBP1 (#9644; 1:1000 dilution), PS6K (#9234; 1:1000 dilution), S6K (#2708; 1:1000 dilution), pULK1-S555 (#5869; 1:500 dilution), pULK1-S757 (#14202; 1:500 dilution), ULK1 (#6439; 1:200 dilution), pACC (#11818; 1:1000 dilution), ATG7 (#8558; 1:1000 dilution), and Beclin1 (#3495; 1:1000 dilution) and rabbit polyclonal antibodies against pAMPK (#2531; 1:200 dilution), AMPK (#2532; 1:1000 dilution), ACC (#3662; 1:1000 dilution), ATG5 (#2630; 1:1000 dilution), lamin B1 (#12586; 1:1000 dilution), tubulin (#2125; 1:1000 dilution) and LAMP1 (#D2D11; 1:200 dilution) were obtained from Cell Signaling Technology (Beverly, MA). Antibody conjugates for FLT3 (APC-anti-CD135, clone A2F10.1; 1:50 dilution), IL7Ra (V450-anti-CD127, clone SB/199; 1:50 dilution), Sca1 (PE-Cy7-anti-Ly-6A/E, clone E13–161.7; 1:50 dilution), and CD34 (FITC-anti-CD34, clone RAM34; 1:50 dilution) were obtained from BD Biosciences (San Jose, CA), while c-kit (PE-anti-CD117, clone REA791; 1:50 dilution) was obtained from Miltenyi Biotech (Bergisch Galadbach, Germany). Additionally, BUV395-anti-Sca1 (clone D7; 1:50 dilution) and PE-Cy7-anti-c-kit (Clone 2B8; 1:50 dilution) antibodies were purchased from BD Biosciences (San Jose, CA) and PE-anti-FLT3 (clone A2F10; 1:50 dilution) was purchased from eBioscience. Rabbit polyclonal antibodies against EEA-1 (1:1000 dilution) and calreticulin (1:1000 dilution) and hsp70 (1:1000 dilution) mouse monoclonal antibodies were obtained from Affinity Bioreagents (Golden, CO). The mouse monoclonal Anti-SQSTM1 (p62) antibody (#610833; 1:500 dilution) was purchased from BD Biosciences, anti-β-actin antibody (#A5316; 1:1000 dilution) was purchased from Sigma (St. Louis, MO) and anti-GFP antibody (sc-9996; 1:1000 dilution) was purchased from Santa Cruz Biotechnology (Santa Cruz, CA). Anti-F4/80 antibody (ab6640; 1:1000 dilution) and anti-β-COP antibody (ab2899; 1:200 dilution) from were obtained from Abcam. Horseradish peroxidase-conjugated anti-rabbit IgG, anti-mouse IgG and anti-goat IgG (1:2000 dilutions) were purchased from Bethyl Laboratories (Montgomery, TX). Alexa 488- and 594-conjugated anti-rabbit, anti-mouse, and anti-goat secondary antibodies (1:2000 dilutions) were obtained from Invitrogen, Thermo Scientific (Waltham, MA).

### Isolation and maintenance of MSCs and HSCs

The mice were euthanized with CO_2_ asphyxiation, and the bone marrow from the femur and tibiae was flushed with stem cell medium [(minimum essential medium with no nucleosides (α-MEM, ThermoFisher Scientific) containing 1% l-glutamine, 10% fetal bovine serum, 100 U/ml of penicillin, and 100 mg/ml of streptomycin)] and filtered through a 40-µm pore size cell strainer (BD Biosciences, Bedford, MA). For HSC isolation, freshly harvested bone marrow cells were c-kit-enriched using c-kit-conjugated magnetic beads (Miltenyi, Bergisch Gladbach, Germany), and subsequently costained with antibodies against lineage markers (Lineage Cell Detection Cocktail, Miltenyi), FLT3 (APC-anti-CD135), IL7Ra (V450-anti-CD127), c-kit (PE-anti-CD117), Sca1 (PE-Cy7-anti-Ly-6A/E), and CD34 (FITC-anti-CD34). In other instances, lineage-positive (Lin^+^) cells were depleted by magnetic-activated cell sorting (MACS) using the MagCellect mouse hematopoietic cell lineage depletion kit (R&D Systems, Minneapolis, MN). The Lin^−^cells from MACS were again gated in FACS for the negative selection of Lin + populations using the APC mouse lineage antibody cocktail (BD Biosciences, San Jose, CA). Next, the Lin^−^ cells were gated using BUV395-anti-Sca1, PE-Cy7-anti-c-kit, PE-anti-FLT3 and FITC-anti-CD34 antibodies and were sorted to obtain Lin^-^Sca1^+^c-kit^+^FLT3^−^CD34^+^ HSCs^53^. The LIVE/DEAD Fixable Near-IR Dead Cell Stain Kit (ThermoFisher Scientific, Waltham, MA) was used to exclude dead cells. Cell staining was performed on ice and analyzed using a BD LSRFortessa equipped with five lasers (355, 405, 488, 561, and 633 nm). Color compensation was performed using BD FACSDIVA Software (BD Biosciences, Bedford, MA) and FACS data analysis was performed using FlowJo (Tree Star, Ashland, OR).

Long-term HSC (LT HSC), short-term HSC (ST HSC), and multipotent progenitor (MPP) cell types were quantified following gating schemes described previously^54^. In short, cells exhibiting a Lin^−^IL7Ra^−^ Sca1^+^c-kit^+^phenotype were resolved into LT HSC, ST HSC, or MPP cell types based on differential expression of CD34 and FLT3^55^. Among Lin^−^IL7Ra^−^ Sca1^+^c-kit^+^ cells, LT HSCs were CD34^−^ and FLT3^−^, ST HSCs were CD34^+^ and FLT3^−^, and MPPs were CD34^+^ and FLT3^+^. HSCs were maintained in HSC medium [serum-free stem cell expansion medium (StemSpan, Stem Cell Technologies) supplemented with Stem Cell Factor (SCF) (100 ng/ml)(R&D systems)] for downstream applications.

For MSCs, the Lin^−^ cells were allowed to adhere for 3 days in stem cell medium. The non-adherent hematopoietic cells were discarded, and the adherent populations were trypsinized and expanded for 2 weeks. BUV395-anti-Sca1, PerCP-Cy5.5-anti-CD44, PE-CF594-anti-CD49e, APC-anti-CD90 and BV510-anti-CD105 antibodies (BD Biosciences) were used to obtain Lin^-^Sca1^+^CD44^+^CD49e^+^CD90^+^CD105^+^ MSCs^53,56^. MSCs were maintained in stem cell medium (as detailed above) for all experiments.

Gating strategies for flow cytometry analysis of HSC and MSC in various experiments are indicated in Supplementary Fig. 13.

### Differentiation of MSCs and HSCs

Adipogenic or osteoblastogenic differentiation of MSCs was induced using established methods^57^. Mineralization was performed by Alizarin red-S staining^57^. Chondrocyte differentiation was induced using a commercial StemPro chondrogenesis differentiation kit (Thermo Scientific, Waltham, MA). Alcian blue staining was used to detect collagen production with chondrocyte differentiation^58^. Myocyte differentiation was induced by treating MSCs with 2% horse serum^59^. Cell nuclei were counterstained with 1 µg/ml of Hoechst 33258 (Sigma-Aldrich). A detailed description of these methods is available in the Supplementary Methods.

HSCs were cultured in complete methylcellulose media, and their differentiation potential was assessed by colony-forming cell assays (MethoCult GF M3434 (Stem Cell Technologies)). Burst-forming units-erythroid (BFU-E), colony-forming units-macrophage (CFU-M), colony-forming units-granulocyte (CFU-G), and colony-forming unit-granulocyte/ macrophage (CFU-GM) were characterized and scored according to their morphology^60^. Osteoclast differentiation of HSCs was induced by 30 ng/ml of M-CSF (R&D Systems) and 50 ng/ml of RANKL (R&D Systems) for 14 days^53^. On day 14, the osteoclasts were fixed and stained with tartrate-resistant acid phosphatase (TRAP) using a leukocyte acid phosphatase kit (Sigma-Aldrich, St. Louis, MO) or were subjected to visualization of actin ring formation using tetramethylrhodamine-phalloidin (Life Technologies, Carlsbad, CA)^53^.

### Retroviral production and transduction

A retroviral packaging cell line (ATCC, CRL 9078) was maintained in Dulbecco’s modified minimum essential medium (DMEM) supplemented with 5% fetal bovine serum. These cells were transfected with retroviral plasmid constructs harboring *SLC29A3* or ΔN36*SLC29A3*^18,20^ for viral production. Cells were incubated with a complex containing 8 μg of each of the plasmids, 24 μl of FuGENE 6 reagent (Roche Applied Science), and 800 μl of Opti-MEM serum-free medium (Invitrogen). After 48 h of transfection, viruses in the supernatant were harvested, filtered, and target cells were transduced with recombinant viruses at 4 μg/ml polybrene to virus concentration.

### Maintenance of *Slc29a3*^−/−^ mice

All animal procedures were performed according to protocols approved by the Ohio State University (OSU) IACUC. The *Slc29a3*^+/-^ gene trap knockout line in a 129S5/SvEvBrd x C57BL6/J hybrid background was obtained from the Mutant Mouse Resource and Research Center. A heterozygote breeding strategy was utilized to generate *Slc29a3*^+/+^, *Slc29a3*^+/-^ and *Slc29a3*^−/-^ littermates. In general, F1 HET mice were crossed to produce F2 WT, HET, and HOM null cohorts, and phenotypic analyses were conducted on 3–12 animals of either sex (unless otherwise stated) in age groups 8–16 weeks as indicated. In addition to littermate WT, HOM null cohorts were also compared to WT reference controls (C57BL/6NCrl) as needed. When HOM null male and female animals were crossed, several breeding pairs were set up to obtain viable litters. For genotyping, tail clips were obtained at 3 weeks of age, and DNA was isolated by proteinase K digestion followed by the isopropanol/ethanol precipitation method. Genotyping was performed by PCR using the following primers: WT forward: GCCAGAGGATCGCTTCA; WT reverse: GACAACAGCAGGCAATCTAAA; KO forward: ATGCAAATGGCCTCCCTCAAGTACC; KO reverse: GAGGAAATTGCATCGCATTGTCT primers. The PCR cycling conditions to generate product sizes of 415 and 284 bp, for WT and KO, respectively, were as follows: WT: denaturation at 94 °C for 2 min; four cycles of 94 °C for 20 s, 62 °C for 20 s, and 72 °C for 20 s; 36 cycles of 94 °C for 20 s, 55 °C for 20 s, and 72 °C for 20 s; and final extension at 72 °C for 1 min; KO: denaturation at 94 °C for 2 min; 40 cycles of 94 °C for 10 s, 58 °C for 20 s, and 72 °C for 20 s; and final extension at 72 °C for 1 min for KO. All the mice were maintained at an ambient temperature of 20–22 °C with a 12-h light/dark cycle and were given free access to standard rodent chow and water.

### Clinical phenotyping of *Slc29a3*^−/−^ mice

The animals were acclimated to the motorized treadmill (model Eco3/6; Columbus Instruments) by running for 5 min at 8 m/min each day for 3 days. On the day of experimentation, an electrical stimulus (163 V, 0.45-mA current, 1 Hz) was applied using electrical shock grids at the rear end of the treadmill that allowed the animals to exercise until exhaustion. The inability to continue treadmill running at treadmill exhaustion was used to evaluate the endurance capacity.

MicroCT images of the tibia were acquired at 80 kVp and 490 mA using a General Electric Locus RS (General Electric HealthCare, UK). The voxel size was 0.020367 × 0.020367 × 0.020367 mm. For image analysis, a region-of-interest (ROI) that included 0.3055 mm above the growth plate was used for trabecular analysis. The trabecula was segmented using a locally adaptive segmentation algorithm. The percent bone and percent marrow were calculated from the segmented trabecula image^61^. The trabecular thickness, spacing, and number were calculated from the Euclidean distance map of the segmented trabecula^61,62^. Bone mineral density measurements for the trabecular region and two cortical regions (approximately 2.67, and 6.5 mm from the growth in the diaphysis of the bone) were calculated using a hydroxyapatite calibration phantom.

The mice were subjected to non-invasive monitoring of whole-body lean and fat mass by Echo MRI at the Small Animal Imaging Facility, OSU^63^.

For bone marrow transplantation experiments, recipient WT or *Slc29a3*^−/−^ mice were given total body irradiation with 9.5 Gy using an RS 2000 × -ray irradiator or 10.5 Gy using a Small Animal Radiation Research Platform (SARRP) system. After irradiation, 1 × 10^4^ bone marrow-derived Lin^-^Sca1^+^c-kit^+^FLT3^-^CD34^+^ WT or *Slc29a3*^−/−^ HSCs in 100 µl of PBS were injected intravenously into the tail vein (details in Supplementary Methods). The mice were monitored for survival over a period of 15 days. The Kaplan–Meier survival plot was created based on the estimation of survival data. Whole bone marrow was collected at the end of 15 days (or at the day of mortality), and the number of bone marrow cells per limb was assessed.

### Lysosome isolation and analyte quantification using liquid chromatography–mass spectrometry (LC-MS/MS)

Enriched lysosomes from the mouse spleen and HEK293 cells were obtained by differential centrifugation, followed by density gradient centrifugation and calcium precipitation (LYSISO1; Sigma-Aldrich, St. Louis, MO). To determine the enrichment and recovery of lysosomes, the total homogenate and different lysosomal fractions were compared for the protein concentration (using BCA protein assay) and acid phosphatase activity (CS0740; Sigma-Aldrich, St. Louis, MO), while the intactness of the lysosomes was assessed using the neutral red dye following manufacturer’s protocol (CS0740; Sigma-Aldrich, St. Louis, MO).

The extraction of adenosine from the purified lysosomal fractions and adenosine and AMP from the cytosolic fractions was performed using 100 µl of cold acetonitrile: methanol (3:1, v/v) spiked with reserpine (1 µg/ml) as the internal standard. The samples were vortex mixed and then were centrifuged at 15,000 rpm at 4 °C for 10 min. The supernatant was filled in auto-sampler vials, and volumes of 10 µl were injected into an LC-MS/MS system. LC-MS/MS analysis was performed using a Thermo Scientific Vanquish UPLC system (Waltham, MA) interfaced with a Thermo Scientific TSQ Quantiva triple-stage quadrupole mass spectrometer (Waltham, MA) equipped with an H-ESI ion source controlled by Thermo XcaliburTM 3.0 data acquisition and analysis software. MS detection was carried out in the positive ionization mode, and the transitions monitored were *m*/*z* 268.12 to 136.11 for adenosine, 348.13 to 136.11 for AMP, 259.00 to 136.11 for AICAR and 609.39 to 195.05 for reserpine. The operational mass spectrometric parameters included the following: capillary voltage, 4.5 kV; sheath gas, 35 arbitrary units; auxiliary gas, 10 arbitrary units; sweep gas, 2 arbitrary units; ion transfer tube temperature, 350 °C; vaporizer temperature, 450 °C; Dwell time, 50 ms per transition; collision-induced dissociation (CID) gas, 1.5 mtorr. The collision energy was set at 20 V for adenosine, AICAR, and AMP, and at 38 V for reserpine. Chromatographic separation was carried out using a Scherzo SMC_18_ column (150 × 4.6 mm; 3-µm particle size) from Imtakt (Portland, OR). The mobile phase consisted of solvent A [water (0.1% formic acid)] and solvent B [acetonitrile (0.1% formic acid)]. The flow rate was set at 0.5 ml/min, and the gradient program used was 0 min: 95% B; 2 min: 95% B; 10 min: 10% B; 11 min: 95% B; 17 min 95% B. The total run time was 17 min per sample, and the auto-sampler was maintained at 4 °C throughout the analysis.

### Untargeted and targeted metabolomics

Metabolites were extracted using a liquid–liquid extraction (LLE) method and a mixture of methanol/chloroform/hexane (2/0.5/0.5, *v/v/v*). Further analyses were carried out using a Thermo Scientific LTQ Orbitrap™ Hybrid Ion Trap-Orbitrap mass spectrometer (MS) and a Waters 2795 HPLC separation module interfaced by an electrospray ionization (ESI) source. The metabolites were separated on a Scherzo SMC_18_ column (150 mm × 4.6 mm; 3-µm particle size; Imtakt, Portland, OR). The mobile phase used for chromatographic separation was as follows: solvent A, 10 mM ammonium acetate:acetonitrile (40:60, *v/v*); solvent B, 2-propanol:acetonitrile (90:10, *v/v*). The optimized chromatographic gradient conditions were as follows: 0 min, 95% A; 2 min, 95% A; 22 min, 10% A; 27 min, 10% A; 28 min, 95% A; 35 min, 95% A; the flow rate was set at 0.5 ml/min. Data acquisition and analysis were conducted using Thermo Xcalibur^TM^ software. The data were collected separately in the positive and negative electrospray ionization modes, and the acquisition was performed in the centroid mode using the full-scan MS method. The general mass spectrometer operating conditions were as follows: Spray voltage: + 4.5 kV for the positive mode and −3.5 kV for the negative mode; source temperature: 350 °C; vaporizer temperature: 450 °C; sheath gas: 35 arbitrary units; auxiliary gas: 10 arbitrary units; sweep gas: 2 arbitrary units; mass scan range: *m*/z 50–1200 at 0.1 scan/s.

For MS/MS analysis, the mass parameters were as follows: isolation width: 2.0 (*m*/*z*); collision-induced dissociation (CID): 40%; resolution: 30,000; maxIT: 100 ms. An open source software package MZmine v.2.21 (http://mzmine.sourceforge.net/) was used for data processing. The RANSAC algorithm was applied to align the detected peaks in the different samples, generating an aligned peak list for *Slc29a3*^+/+^ & *Slc29a3*^−/−^ samples. A database search using the Human Metabolome Database (HMDB) (http://www.hmdb.ca), KEGG pathway database (http://www.genome.jp/kegg/) & Lipid Metabolites and Pathway Strategy (Lipid Maps) (http://www.lipidmaps.org) was performed to establish peak identities for each *m/z* in the aligned peak list with a mass tolerance set to ± 10 ppm. Lipidomics analysis of the liver samples from *Slc29a3*^+/+^ & *Slc29a3*^−/−^ was performed using the method described by Park et al.^64^

Functional enrichment of the metabolome data with KEGG pathway annotations was performed using MBROLE2^65^ (http://csbg.cnb.csic.es/mbrole2/). Integrated pathway analysis of transcriptome and metabolome data was performed using MetaboAnalyst^®^3.0^66^ by employing the KEGG pathway database. The integrated metabolomics and transcriptomics network was constructed based on the HMDB ID for metabolites and gene symbols for transcripts using the open source software GAM^67^ (https://artyomovlab.wustl.edu/shiny/gam), ipath^68^, and LRpath^69^.

### Transmission electron microscopy

Liver sections from animals or MSCs were fixed with 2% glutaraldehyde for 3 h. After washing in phosphate buffer (0.1 M), the tissues were processed for secondary fixation with 1% osmium tetroxide for 1 h, followed by ethanol dehydration, infiltration, and embedding in Eponate 12 resin for ultrathin sectioning using a Reichert Ultracut E ultramicrotome. The sections were stained using 2% aqueous uranyl acetate followed by Reynolds lead citrate and observed by TEM using FEI Technai Spirit electron microscope at 80 kV. For scoring autophagosomes and/or lysosomes in TEM studies, slide identities were blinded and predefined stereoptic criteria were used to quantify structures of interest.

### Immunocytochemical analysis

Cells were plated on coverslips in six-well clusters and were allowed to grow until they were 60–80% confluent. The cells were fixed with 4% paraformaldehyde (PFA), rinsed with PBS, permeabilized with 0.1% Triton X-100, and incubated in a standard blocking solution (10% donkey serum, 1% BSA in PBS) for 1 h. The cells were then incubated overnight at 4 °C with primary antibody at a 1:200 to 1:1000 dilution. Subsequently, the cells were washed with PBS and incubated for 1 h with a 1:1000 dilution of Alexa Fluor 647 goat anti-rabbit IgG (Invitrogen) or Alexa Fluor 647 donkey anti-Goat IgG (Invitrogen) secondary antibody, respectively. Nuclei were stained with DAPI (Thermo Fisher Scientific) and examined using an Olympus Spectral FV1000 confocal microscope or Nikon ti2 inverted fluorescence microscope.

### Gene expression analysis

Total RNA was extracted from cells using the E.Z.N.A Total RNA Kit (Omega Biotek, Norcross, GA) following the manufacturer’s protocol, and the RNA integrity was measured using the Agilent 2100 Bioanalyzer (Agilent Technologies, Palo Alto, CA). One microgram of total RNA was reverse transcribed using the RevertAid RT Reverse Transcription kit (Thermo Fisher Scientific, Waltham, MA). Quantitative PCR was performed using a StepOnePlus Real-Time PCR System (Applied Biosystems, CA, USA). The Ct values for GAPDH and GusB were used to normalize the expression level of the gene of interest using the ∆∆CT method^70^. The PrimerBank web application was used to design the exon-spanning primers listed below.

BSPII-F: GACTTTTGAGTTAGCGGCACT; BSPII-R: CCGCCAGCTCGTTTTCATC; BGLAP-F: CTGACCTCACAGATCCCAAGC; BGLAP-R: TGGTCTGATAGCTCGTCACAAG; OSX-F: ACCCCAAGATGTCTATAAGCCC; OSX-R: CGCTCTAGCTCCTGACAGTTG; Runx2-F: GACTGTGGTTACCGTCATGGC; Runx2-R: ACTTGGTTTTTCATAACAGCGGA; PPARγ-F: TCGCTGATGCACTGCCTATG; PPARγ-R: GAGAGGTCCACAGAGCTGATT; CEBPα-F: GCGGGAACGCAACAACATC; CEBPα-R: GTCACTGGTCAACTCCAGCAC; Adiponectin- F: AGCCGCTTATATGTATCGCTCA; Adiponectin-R: TGCCGTCATAATGATTCTGTTGG Chemerin-F: TTGCTGATCTCCCTAGCCCTA; Chemerin-R: TGGGTGTTTGTGGAACTCCTC; MyoD-F: GACAGGGAGGAGGGGTAGAG; MyoD-R: TGCTGTCTCAAAGGAGCAGA; Myogenin-F: CCGAGCCTCTACAACAGGAG; Myogenin-R: GGTAACTTCCTCACCCACGA; MYH1-F: GCGAATCGAGGCTCAGAACAA; MYH1-R: GTAGTTCCGCCTTCGGTCTTG; DES-F: CCTGGAGCGCAGAATCGAAT; DES-R: TGAGTCAAGTCTGAAACCTTGGA; SLC29A3-F: GGGCATATAAACTCCGAAACTGC; SLC29A3-R: GGAGGAAGTATCCACCTTCACC; PAX1-F: CCGCCTACGAATCGTGGAG; PAX1-R: CCCGCAGTTGCCTACTGATG; SOX9-F: AGTACCCGCATCTGCACAAC; SOX9-R: ACGAAGGGTCTCTTCTCGCT; ACAN-F: GTGGAGCCGTGTTTCCAAG; ACAN-R: AGATGCTGTTGACTCGAACCT; COMP-F: ACTGCCTGCGTTCTAGTGC; COMP-R: CGCCGCATTAGTCTCCTGAA; GATA1-F TGTCCTCACCATCAGATTCCA; GATA1-R: TCCCTCCATACTGTTGAGCAG; EKLF1-F: TCTGAGGAGACGCAGGATTTG; EKLF1-R: ACAGGTCACGTCCCTCTCATC; GYPA-F: TACCAAGAAGAGCATTCACCATC; GYPA-R: TGCTGATTTGGGTTACCTACAGT; Epb4.2-F: CCACGCAGCAGAAAACAACG; Epb4.2-R: GAGCACGGAAGTTCAGGGT; PU.1-F: TTACAGGCGTGCAAAATGGAA; PU.1-R: GACGTTGGTATAGCTCTGAATCG; REL-F: AGAGGGGAATGCGGTTTAGAT; REL-R: TGTCCGGTTGTTGTCTGTGC; CD11b-F: CCATGACCTTCCAAGAGAATGC; CD11b-R: ACCGGCTTGTGCTGTAGTC; CD14-F: ACTTCTCAGATCCGAAGCCAG; CD14-R: CCGCCGTACAATTCCACAT; LGR5-F: ACATTCCCAAGGGAGCGTTC; LGR5-R: ATGTGGTTGGCATCTAGGCG; EPHB3-F: CTGTGCGTGCCTTCTACAAGA; EPHB3-R: GCTTGAGTGGTACAGAGACCTC; AXIN2-F: AACCTATGCCCGTTTCCTCTA; AXIN2-R: GAGTGTAAAGACTTGGTCCACC; LEF1-F: CCAGAGAACACCCTGATGAAGG; LEF1-R: GGCACTTTATTTGATGTCCTCGG; HES1-F: TCAACACGACACCGGACAAAC; HES1-R: ATGCCGGGAGCTATCTTTCTT; HEY1-F: CCGACGAGACCGAATCAATAAC; HEY1-R: TCAGGTGATCCACAGTCATCTG; HEY2-F: GAGGTCCAATTCACCGACAAC; HEY2-R: AGCATGGGCATCAAAGTAGCC; NRARP-F: TTCAACGTGAACTCGTTCGGG; NRARP-R: TTGCCGTCGATGACTGACTG; REX1-F: AAGCTGCCAGCCAGTAACC; REX1-R: CCTTGCGTTCCACCAACTTTC; TERT-F: CTGAGTCTCACCAGTACAAGTGT; TERT-R: TTGGCACCCATGATTTGCCT; NANOG-F: AGGACAGGTTTCAGAAGCAGA; NANOG-R: CCATTGCTAGTCTTCAACCACTG; SOX2-F: CCCACCTACAGCATGTCCTAC; SOX2-R: GCCTCGGACTTGACCACAG; GAPDH-F: TGGCCTTCCGTGTTCCTAC; GAPDH-R: GAGTTGCTGTTGAAGTCGCA

### Statistical analyses

Comparisons between values were performed using Student’s *t*-test (two-tailed), unless otherwise indicated, after confirming that the data met appropriate assumptions (normality, homogenous variance, and independent sampling). All comparisons between multiple groups were performed using one-way or two-way analysis of variance (ANOVA) with Tukey’s post-hoc analysis. For all statistical analyses, *P* < 0.05 was considered statistically significant, unless otherwise indicated. The results are expressed as the means ± SEM, unless otherwise mentioned.

Multivariate statistical analysis was performed for the identified metabolite peak lists using MetaboAnalyst^®^ 3.0. Principal component analysis (PCA), partial least squares discriminant analysis (PLS-DA) and orthogonal projection to latent structures discriminant analysis (OPLS-DA) were used to visualize clustering of the *Slc29a3*^+/+^ & *Slc29a3*^−/−^ samples. Unsupervised hierarchical clustering for data overview was performed using a heatmap generated using a *t*-test, the Euclidean distance measurement, and the Ward clustering algorithm.

For all in vitro experiments, a sample size of *n* = 3–12 was used based on the effect size and overlap between distributions. A power of 0.8 was set as minimal to decide sample size for each experiment. For the in vivo studies that assessed the effects of AICAR treatment or stem cell transplant on survival of *Slc29a3*^−/−^ mice, a sample size of *n* = 10–16 independent mice per group was used to include inherent variabilities in survival times among *Slc29a3*^−/−^ mice. Sample sizes were estimated using power calculations for guidance (http://biomath.info/power/ttest.htm) with effect sizes adjudged from pilot studies. With alpha = 0.05 and power = 0.9 and allowing for some unexpected mortalities (~10% mouse/group), ≥ 7–8 mice per group were needed for in vivo studies. For in vivo bone marrow transplant mice study, a sample size of *n* = 6 independent mice per group was set because a definitive end point mortality within 7 days post irradiation was achieved in all nontransplanted mice. The sample size for the metabolomics and lipidomics experiments was calculated based on the power analysis module in Metaboanalyst software^66^, which uses algorithms described by van Iterson et al.^71^. The desired power of 0.8 was achieved for both the metabolomics and lipidomics data validating a sample size of *n* = 12 independent mice per group. No animals or samples were excluded from the analyses.

### Reporting summary

Further information on research design is available in the Nature Research Reporting Summary linked to this article.

## Supplementary information

Supplementary Information

Reporting Summary

Description of Additional Supplementary Files

Supplementary Data 1

Supplementary Data 2

Source Data

## Data Availability

The data sets generated during and/or analyzed during the current study are available within the article and its Supplementary Information files, or from the corresponding author on reasonable request. A reporting summary for this Article is available as a Supplementary Information file. The source data underlying Figs. 1–9 and Supplementary Figs. 4–7, 10 and 12 are provided as a Source Data file. Metabolomics and lipidomics data have been deposited to the EMBL-EBI MetaboLights database (10.1093/nar/gks1004. PubMed PMID: 23109552) with the identifier MTBLS949.
