# Supplementary material for: Adult stem cell deficits drive Slc29a3 disorders in mice
Source: Nat Commun. 2019 Jul 3;10:2943. doi: 10.1038/s41467-019-10925-3 (PMC6610100; doi:10.1038/s41467-019-10925-3)
Supplement: Supplementary file 1 — Supplementary Information [file 41467_2019_10925_MOESM1_ESM.pdf]

Supplementary Information

Adult stem cell deficits drive *Slc29a3* disorders in mice

Nair, *et al.*

Supplementary Figures

Supplementary Fig. 1

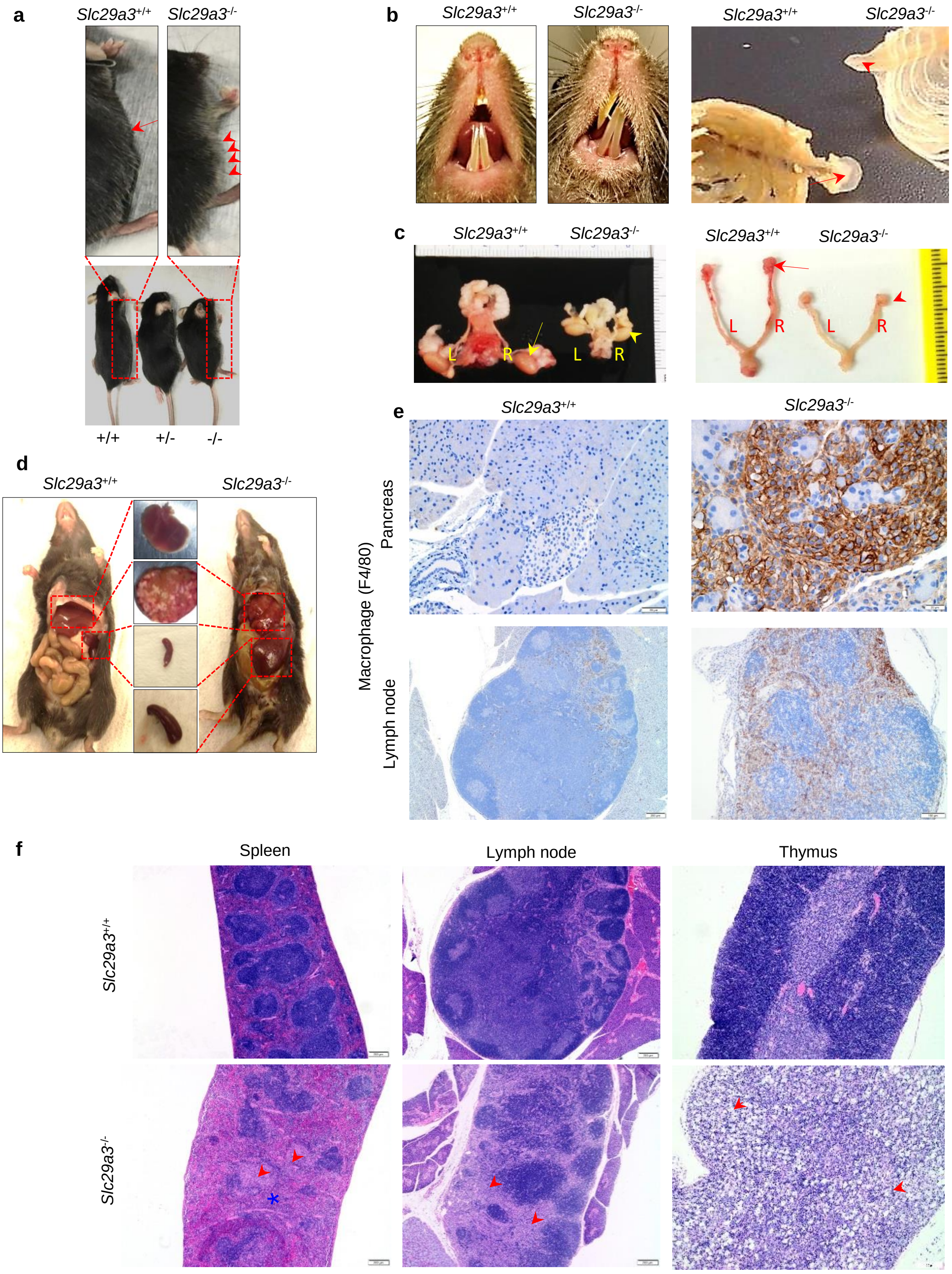

Supplementary Fig. 1: *Slc29a3*$^{-/-}$ mice recapitulate the hENT3 disease features. Hypertrichosis of the body coat (a), malocclusion (b; left) and defective sternal cartilage formation (b; right) in a subset of *Slc29a3*$^{-/-}$ mice. Hypogonadism (testicles (left); ovaries (right)) in a subset of *Slc29a3*$^{-/-}$ mice (c). Hepatosplenomegaly in *Slc29a3*$^{-/-}$ mice (d). The pancreas shows multifocal infiltration of intensely stained histiocytes, and the lymph node shows multifocal expansion of histiocytes to the cortical, medullary and subcapsular sinuses. Macrophages are stained with anti-F4/80 antibody (brown). Scale bar: 50 µm (e). The spleen shows white pulp atrophy with marked histiocytic infiltration (blue asterisk) and multifocal hematopoietic cell apoptosis/necrosis (red arrowheads) Scale bar: 200 µm; the submandibular lymph node shows enlargement and increased lymphocyte apoptosis with histiocytic infiltration and tingible body macrophages (red arrowheads). Scale bar: 100 µm; the thymus shows increased apoptosis/necrosis of lymphocytes (red arrowheads) disrupting the normal cortex and medulla. Scale bar: 100 µm (f). All phenotypes assessed in *Slc29a3*$^{-/-}$ mice at 12 weeks of age compared with litter-mate *Slc29a3*$^{+/+}$ controls. The arrows point at normal structures, and the arrowhead(s) point at abnormalities. L-left; R-right.

Supplementary Fig. 2

Dysfunctions associated with human *SLC29A3* disorders	Associated *SLC29A3* mutations	Analogous phenotypes identified in *Slc29a3*$^{-/-}$ mice	References (Pubmed ID)
Hematological features (erythroid hypoplasia, anemia, thrombocytopenia, monocytosis, lymphocytosis, elevated ESR)	pG427S (c.300+1G>C), pR134H (c.401G>A), pS184R (c.552C>G), pR363T (c.1087C>T), pT449R (c.1346 C>G), pY314T (c. 940delT), pE444X (c. 1330 G>T), pM116R (c. 347 T>G), pG437R (c. 1309 G>A), pA367T (c. 1099 G>A), c.1269_1270delinsA	Hematological features (erythroid hypoplasia, multifocal erythroid necrosis in spleen and lymph nodes, blanched appearance of bone medulla at end stage, anemia, thrombocytopenia, monocytosis, neutrophilia, eosinophilia, elevated ESR)	23623699, 24894595, 20399510, 19889517, 19336477, 26015165, 25963354
Hepatosplenomegaly	pG427S (c.300+1G>C), pR363T (c.1087C>T), pR363Q (c. 1088G>A), c.1045delC, pT449R (c.1346 C>G), pY314T (c. 940delT), pE444X (c. 1330 G>T), pM116R (c. 347 T>G), pG437R (c. 1309 G>A), pR133C (c. 400 C>T), c.243delA, pA367T (c. 1099 G>A), c.1269_1270delinsA	Hepatosplenomegaly (splenomegaly more common than hepatomegaly), Extramedullary hematopoiesis	23623699, 19889517, 18940313, 19336477, 20199539, 23058913, 26015165, 25963354
Lymphadenopathy	pG427S (c.300+1G>C), pR363T (c.1087C>T), pT449R (c.1346 C>G), pY314T (c. 940delT), pE444X (c. 1330 G>T), pM116R (c. 347 T>G), pG437R (c. 1309 G>A), pR133C (c. 400 C>T), c.243delA, pA367T (c. 1099 G>A), pG2098R (c. 625 G>A), P324L (c.971 C>T), c.1269_1270delinsA	Generalized lymphadenopathy, Extramedullary hematopoiesis	23623699, 19889517, 19336477, 20199539, 23058913, 26015165, 24172204, 25963354
Cutaneous lesions (hypertrichosis, epidermal thickening, hyperpigmentation, skin induration and sclerosis)	pG427S (c.300+1G>C), IVS1+2T>G, pR386Q (c.1157G>A), pG427S (c.1279G>A), pG437R (c.1309 G>A), pS184R (c.552C>G), pR363T (c.1087C>T), pR363Q (c.1088G>A), c.1045delC, pT449R (c.1346 C>G), pY314T (c. 940delT), pE444X (c. 1330 G>T), pM116R (c. 347 T>G), pR133C (c. 400 C>T), c.243delA, pA367T (c. 1099 G>A), P324L (c.971 C>T), c.1269_1270delinsA	Hypertrichosis, Increased skin fibroblast proliferation (Ki67 staining), CB57 mouse background precludes hyperpigmentation testing	23623699, 22653152, 20140240, 24894595, 20399510, 19889517, 18940313, 19336477, 20199539, 23058913, 26015165, 24172204, 25963354
Massive widespread histiocytosis	pG427S (c.300+1G>C), pR363T (c.1087C>T), pG2098R (c. 625 G>A)	Massive widespread histiocytosis	23623699, 19889517, 26015165
Skeletal deformities (stunted growth, hallux valgus, camptodactyly, lateral tibial torsion, arthrogryposis, cartilage defects, dysosteosclerosis, dysmorphic skeletal features)	pG427S (c.300+1G>C), c.300+1 G>A, pG437R (c.1309 G>A, c.307delTTT), pS203P (c.607T>C), pR386Q (c.1157 G>A), pT449R (c.1346 C>G), pR134H (c.401G>A), pR363T (c.1087C>T), pR363Q (c,1088G>A), c.1045delC, pR133C (c. 400 C>T), c.243delA, pA367T (c. 1099 G>A), c.1269_1270delinsA	Skeletal deformities (stunted long bones, kyphosis, translucent costal ribs, poor sternum formation, poor cartilage formation, poor trabecular bone mineralization, malocclusion, ectopic calcification).	23623699, 20140240, 22875837, 24894595, 19889517, 18940313, 20199539, 23058913, 26015165, 25963354
Endocrinopathy (hypogonadism, infertility, stunted growth)	pG427S (c.300+1G>C), pG427S (c.1279G>A), pG437R (c.1309 G>A), pT449R (c.1346 C>G), pY314T (c. 940delT), pE444X (c. 1330 G>T), pM116R (c. 347 T>G), pR133C (c. 400 C>T), c.243delA	Endocrinopathy (hypogonadism, high serum androgens and estrogens, poor fertility, stunted growth)	23623699, 24894595, 20140240, 19336477, 20199539, 23058913
Genital Abnormalities (micropenis, gynecomastia)	pG427S (c.300+1G>C), pR363T (c.1087C>T), c.1045delC, pR133C (c. 400 C>T), pA367T (c. 1099 G>A), P324L (c.971 C>T), c.1269_1270delinsA	Genital abnormalities at the end-stage of disease (penile prolapse and vaginal prolapse that may be associated with aberrant mineral homeostasis)	23623699, 19889517, 18940313, 20199539, 26015165, 24172204, 25963354
IDDM (Insulin dependent diabetes mellitus)	pG427S (c.300+1G>C), pG427S (c.1279G>A), pT449R (c.1346 C>G), pY314T (c. 940delT), pE444X (c. 1330 G>T), pM116R (c. 347 T>G), pG437R (c. 1309 G>A), c.243delA, pA367T (c. 1099 G>A), pG2098R (c. 625 G>A)	Mild glucose intolerance	23623699, 24894595, 19336477, 23058913, 26015165
RDD symptoms (emperipolesis, Elevated S100)	pR363T (c.1087C>T), pG2098R (c. 625 G>A)	Elevated S100A8, S100A9 levels	22356918, 26015165
Cardiac anomalies (Cardiomegaly, systolic murmor, pericarditis, ventricular hypertrophy, agenesis of inferior vena cava)	pG427S (c.300+1G>C), c.300+1 G>A, pG437R (c.1309 G>A, c.307delTTT), pS184R (c.552C>G), c.1045delC, pT449R (c.1346 C>G), pY314T (c. 940delT), pE444X (c. 1330 G>T), pM116R (c. 347 T>G), c.243delA, c.1269_1270delinsA	Sudden death	23623699, 20140240, 20399510, 18940313, 19336477, 23058913, 25963354
Sensori-neural hearing loss/Congenital deafness	pG427S (c.300+1G>C), pG427S (c.1279G>A), pG437R (c.1309 G>A), pS184R (c.552C>G), pR363T (c.1087C>T), pR363Q (c,1088G>A), c.1045delC, pT449R (c.1346 C>G), pY314T (c. 940delT), pE444X (c. 1330 G>T), pM116R (c. 347 T>G), P324L (c.971 C>T), c.1269_1270delinsA	Not tested	23623699, 24894595, 20140240, 19889517, 20399510, 18940313, 19336477, 24172204, 25963354
Lipodystrophy	Unknown	Hypocholesterolemia, hypotriglyceridemia, decreased plasma LDL and HDL	-
Pancreatic Insufficiency	pT449R (c.1346 C>G), pY314T (c. 940delT), pE444X (c. 1330 G>T), pM116R (c. 347 T>G)	Abnormal pancreatic architecture with extensive loss of cellularity	19336477
Mental retardation, low IQ	pG427S (c.300+1G>C), c.1269_1270delinsA	Not tested	23623699, 25963354
Myelofibrosis, decreased fat stores, lactic Acidosis	Unknown	Decreased fat mass, increased plasma LDH levels	-

Supplementary Fig. 2: Comparisons of the ENT disease phenotypes in humans and *Slc29a3*$^{-/-}$ mice.

The human symptoms associated with *SLC29A3* mutations were aligned with the observed phenotype in *Slc29a3*$^{-/-}$ mice. The columns represent human symptoms, associated mutations in *SLC29A3*, analogous phenotype in *Slc29a3*$^{-/-}$ mice and relevant references. The color-coding associates each *SLC29A3* mutation to its reference.

Supplementary Fig. 3

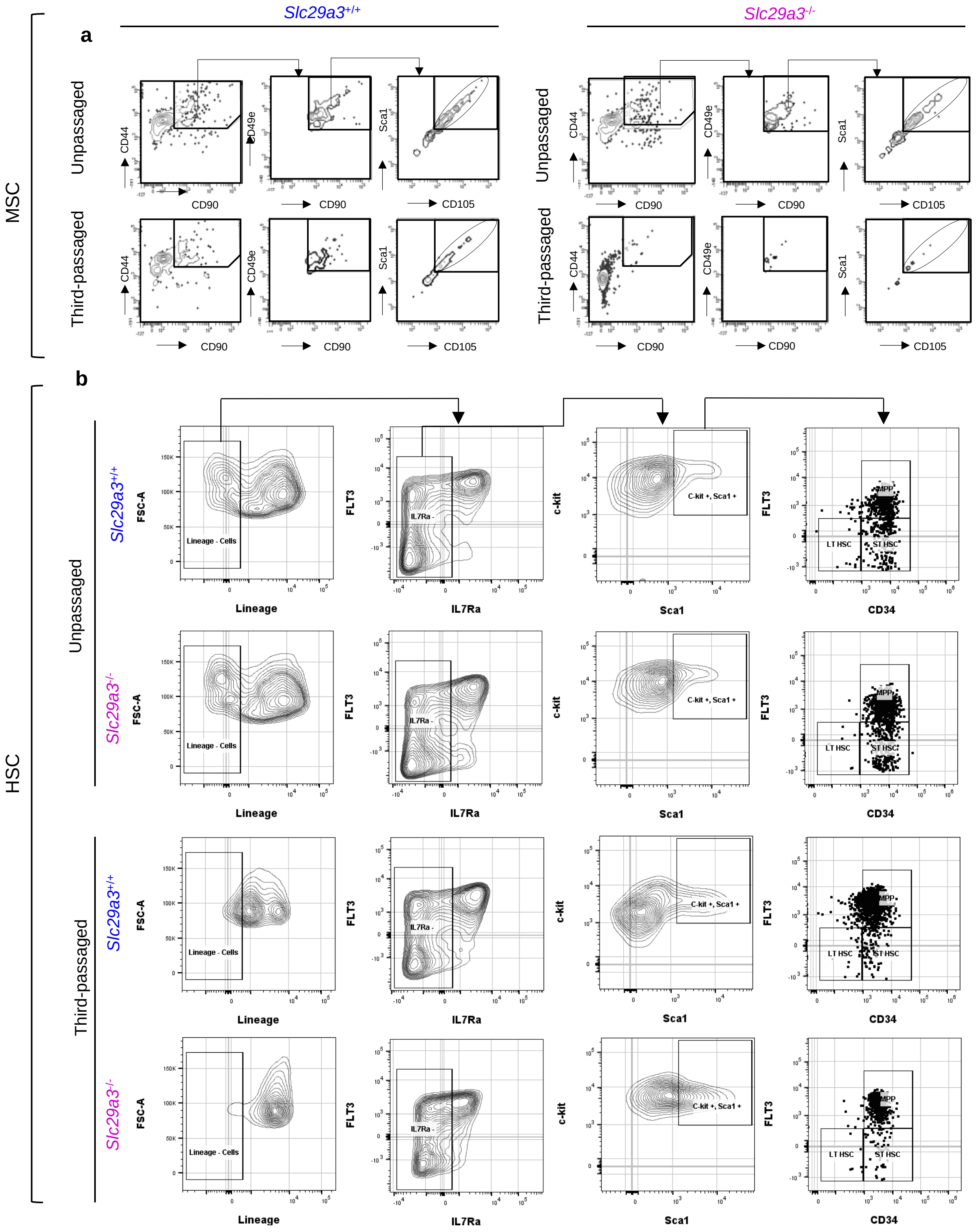

Supplementary Fig. 3: Impaired self-renewal in *Slc29a3*$^{-/-}$ mice. Representative flow cytometric dot plots show CD44^{+} CD49e^{+} Sca1^{+} CD90^{+} CD105^{+} MSC frequencies subjected to self-renewal on the serial passage of CFU-F clones (a). Representative flow cytometric dot plots show Lin^{-} Sca1^{+} c-kit^{+} FLT3^{-} CD34^{+} HSC frequencies (post-c-kit enriched) subjected to self-renewal on the serial passage of HSCs. The Lin^{-} IL7Ra^{-} Sca1^{+}c-kit^{+} HSC pool was further resolved into LT HSC, ST HSC, or MPP cell types based on CD34 and FLT3 expression (b).

Supplementary Fig. 4

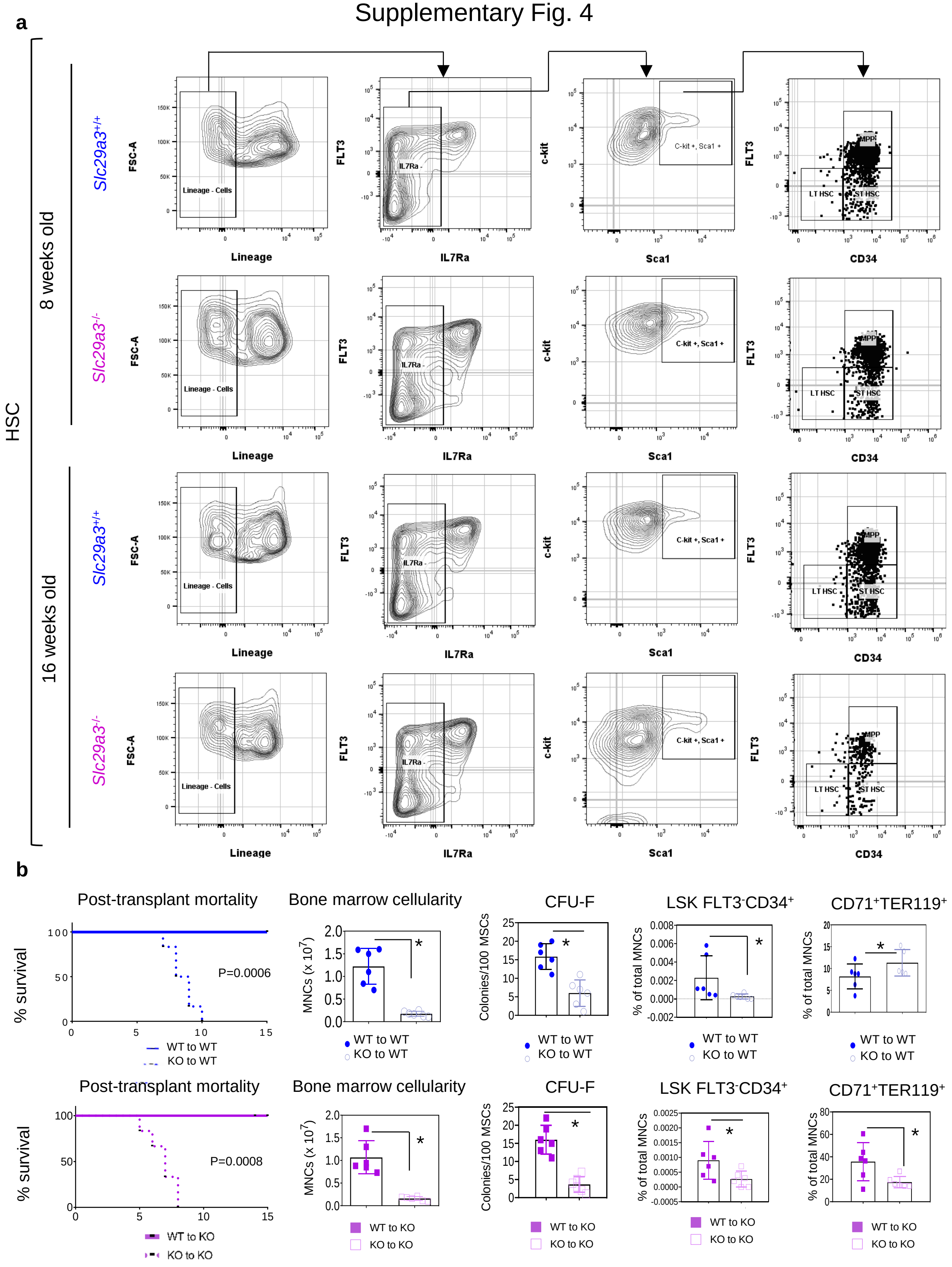

Supplementary Fig. 4: Bone marrow exhaustion in *Slc29a3*$^{-/-}$ mice. Representative flow cytometric contour plots show the Lin^{-} IL7Ra^{-} Sca1^{+}c-kit^{+} HSC pool post c-kit enrichment in 8-week-old *Slc29a3*$^{-/-}$ mice compared with 16-week-old *Slc29a3*$^{-/-}$ mice. The Lin^{-} IL7Ra^{-} Sca1^{+}c-kit^{+} HSC pool was further resolved into LT HSC, ST HSC, or MPP cell types based on CD34 and FLT3 expression (a). Cell autonomous defects in *Slc29a3*$^{-/-}$ Lin^{-} Sca1^{+} c-kit^{+} FLT3^{-} CD34^{+} HSCs identified by HSC transplantation in opposite donor-recipient combination experiment (b). Lethally irradiated mice were used to assess HSC function (n=6, mean±SEM). Statistical analyses were performed by ANOVA with Tukey's multiple comparisons post-test and two-tailed Student's t test. *P < 0.05. Source data are provided as a Source Data file.

Supplementary Fig. 5

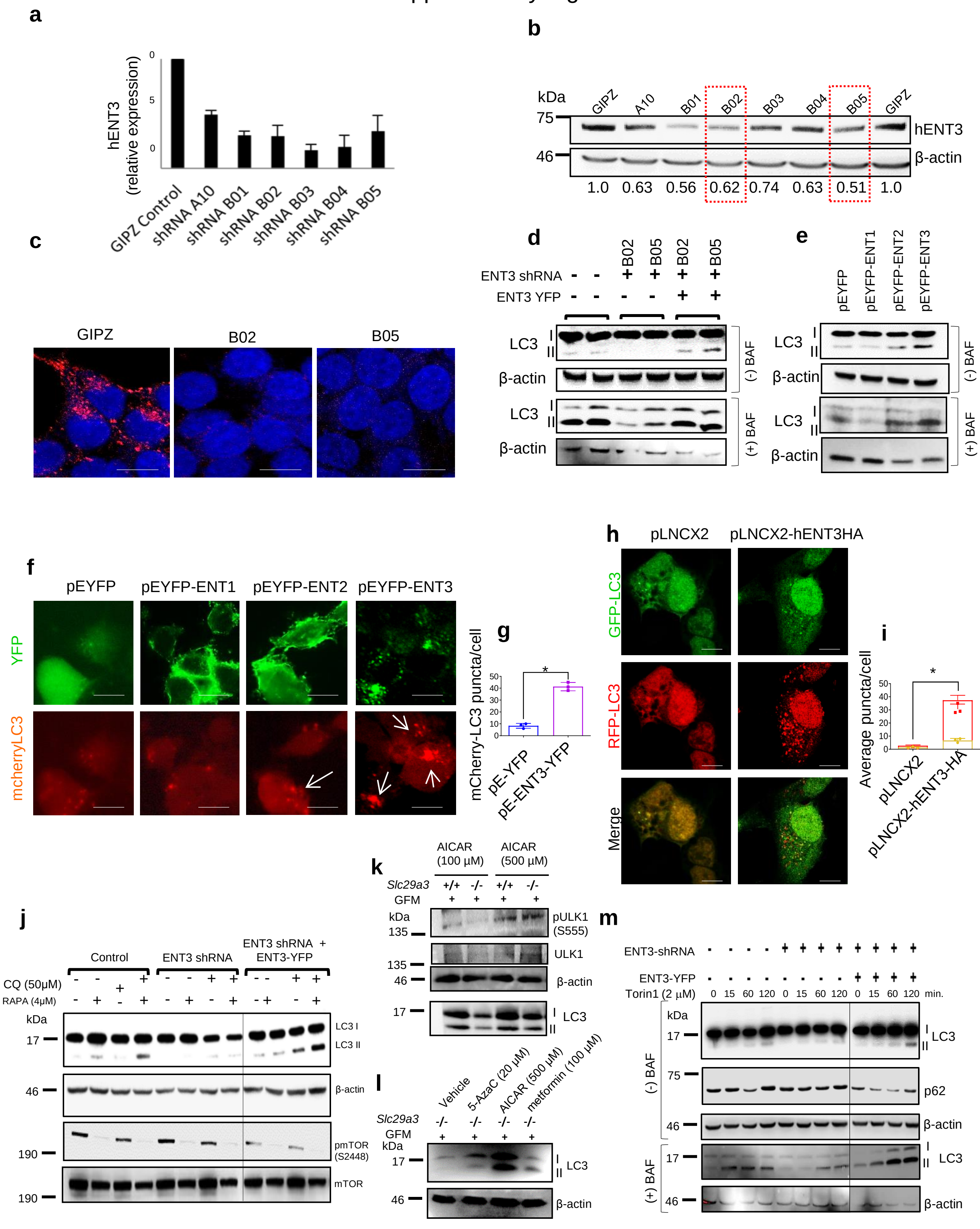

Supplementary Fig. 5: ENT3 regulates autophagy in HEK293 cells.
HEK293 cells were infected with lentivirus harboring the indicated shRNAs and hENT3 transcript and protein expression analyzed by real-time PCR (a) and immunoblotting (b), respectively. Densitometry quantification of the hENT3/β-actin ratio (b, below). Immunofluorescence for hENT3 (red) in the GIPZ control, B02 ENT3 KD and B05 ENT3 KD clones. Nuclei stained with DAPI (blue). Scale bars: 100 µm, original magnification, ×60 (c). hENT3 shRNA diminishes and ENT3-YFP restores autophagic response. BAF, bafilomycin A1 (100 nM, 4 h) (d). Autophagy occurs in an ENT3-specific manner. BAF, bafilomycin A1 (100 nM, 4 h) (e). Representative images of HEK293 cells co-transfected with the mCherry-LC3 plasmid and either pEYFP, pEYFP-hENT1, pEYFP-hENT2 or pEYFP-hENT3 plasmids showing autophagosome formation (arrows) that appear as mcherry-LC3 puncta (red). Nuclei (blue) stained with DAPI. Scale bars: 100 µm (f). Quantification of mcherry-LC3 puncta in YFP- and ENT3YFP-expressing cells. Utilizing Image J, 50 cells were counted in each group and represented (n=3, mean±SEM) (g). HEK293 cells were co-transfected with tandem GFP-RFP LC3 and LNCX2 or LNCX2-hENT3-HA, and RFP LC3 puncta-positive cells and yellow puncta-positive cells (from merged images) were visualized (Scale bars: 10 µm) (h), quantified and depicted in red and yellow sections of the bars, respectively. Fifty cells were counted in each group and represented (n=3, mean±SEM) (i). HEK293 cells expressing control shRNA, hENT3-shRNA and hENT3shRNA+pEYFP-ENT3 were treated with 50 µM chloroquine (CQ) or 4 µM rapamycin (RAPA) or both for 4 h and then were probed for LC3, pmTOR, mTOR and β-actin. Lanes separated by a black line were noncontiguous but were run and exposed at the same time (j). Concentration-dependent effect of AICAR on pULK at Ser555 and LC3 forms in GFM-treated *Slc29a3*$^{-/-}$ MSCs (k). Effect of metformin and 5-azacytidine (4 h treatment) on LC3-II formation in *Slc29a3*$^{-/-}$ MSCs cultured in GFM compared with AICAR treatment (l). Representative immunoblots showing the influence of ENT3 shRNA (middle four lanes) and ENT3-YFP (last four lanes) on time-dependent changes in the protein expression of p62 and LC3B induced by Torin1 in HEK293 cells (first four lanes). Experiments were conducted in the presence (above) or absence (below) of BAF (100 nM). Lanes separated by a black line were non-contiguous but were run and exposed at the same time (m). β-Actin served as the loading control. Source data are provided as a Source Data file.

Supplementary Fig. 6

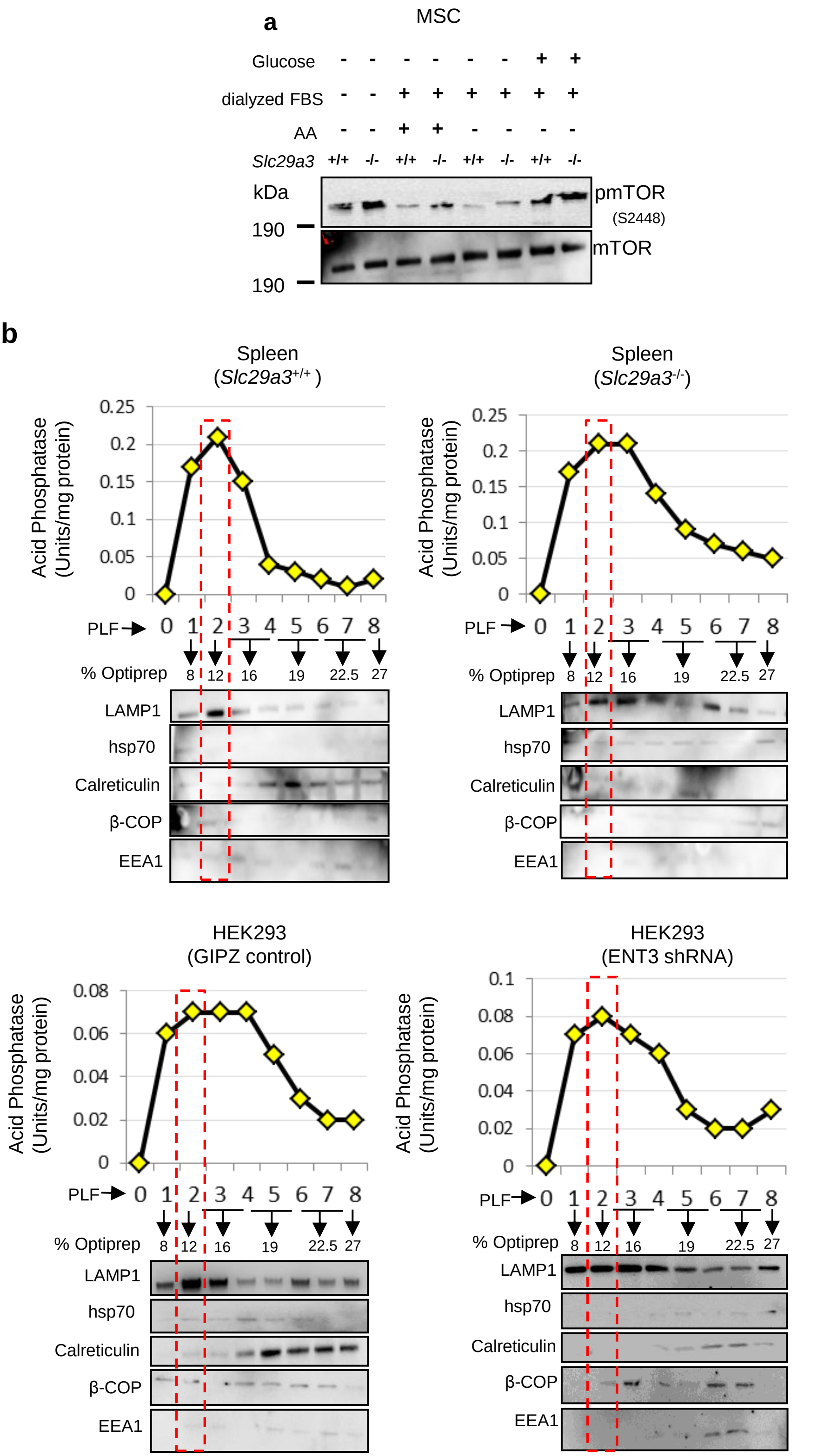

Supplementary Fig. 6. Glucose and aminoacid effects on mTOR phosphorylation and purification of lysosomes. Representative immunoblot of total and phosphor-mTOR expression in MSCs derived from 12-week-old mice when cultured in dialyzed fetal bovine serum (16 h; to remove amino acids and glucose) in the presence or absence of exogenously added essential aminoacids (AA) or glucose (concentrations twice that in growth media, 12 h) (a). Lysates prepared from *Slc29a3*$^{+/+}$ and *Slc29a3*$^{-/-}$ mouse spleens and HEK cells expressing GIPZ or ENT3 shRNA were subjected to differential centrifugation followed by density gradient centrifugation and calcium precipitation to separate purified lysosomal fractions (PLF). After an initial assessment of the integrity of lysosomes using neutral red assay, the purity of lysosomes was assessed by measuring acid phosphatase levels (above) and examining LAMP1 abundance by Western blotting analysis (below). Immunoblots containing various purified lysosomal fractions (PLFs) were also probed with antibodies against heat shock protein 70 (hsp70), calreticulin, coatomer subunit β (β-COP) and early endosomal antigen-1 (EEA-1) to assess possible contamination of mitochondria, the endoplasmic reticulum, the Golgi and early endosomes, respectively (below). The fraction that showed maximal enrichment for acid phosphatase and LAMP-1, but not for other proteins (red dotted box), was used for mass spectrometry analysis in Fig. 6b. Source data are provided as a Source Data file (b).

Supplementary Fig. 7

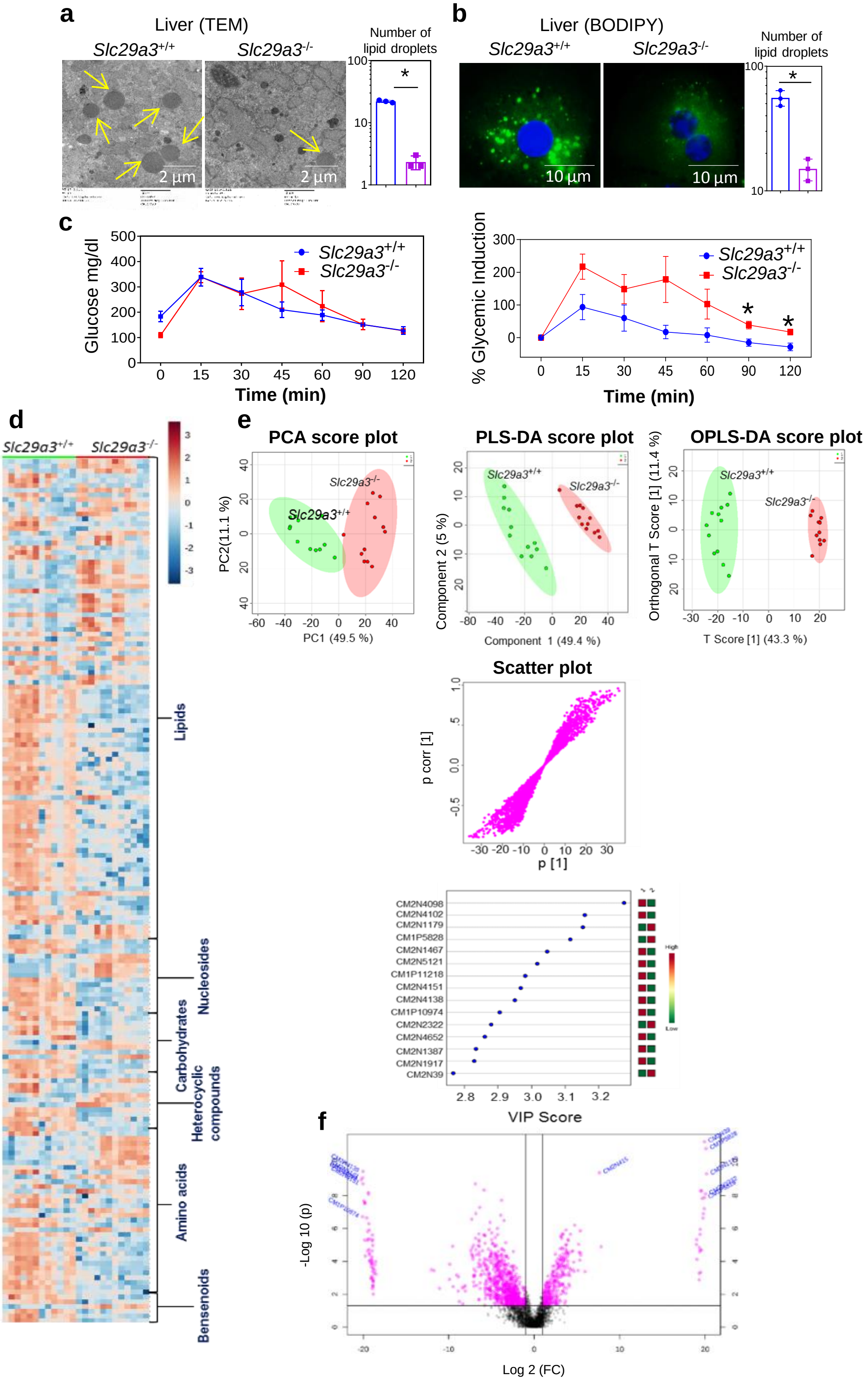

Supplementary Fig. 7. Untargeted metabolomics of the *Slc29a3*$^{-/-}$ adult liver. TEM analysis (left) and quantification (right) of lipid droplets (yellow arrows) in the *Slc29a3*$^{-/-}$ liver derived from 12 weeks old mice. Scale bar: 2 µm (n=3, mean±SEM) (a). BODIPY staining (green, left) and quantification of neutral lipid droplets (right) in cultured hepatocytes after oleic acid (1 µM) treatment. Nuclei stained with DAPI (blue). Scale bar: 10 µm (n=3, mean±SEM) (b). Blood glucose levels measured using a Contour Blood Glucose Monitoring System at the indicated time intervals before (0 h) or after intraperitoneal injection of glucose (2 g/kg) (left) in *Slc29a3*$^{+/+}$ and *Slc29a3*$^{-/-}$ mice (n=3, mean±SEM). The glycemic response was expressed as the percent of the basal fasting glucose levels (right) (n=3, mean±SEM) (c). Heatmap representing significantly altered metabolites (based on non-parametric two-sided t-test) across 12 liver samples from *Slc29a3*$^{-/-}$ mice compared with *Slc29a3*$^{+/+}$ mice (at 12 weeks) and arranged according to the super metabolic class. The rows in heatmap represent the metabolites, and the columns indicate individual liver samples (d). The PCA score plot (green dots: *Slc29a3*$^{+/+}$ and red dots: *Slc29a3*$^{-/-}$), constructed from the combined positive and negative mode untargeted LC-MS analysis, shows clear inter-group separation and intra-group clustering between the *Slc29a3*$^{-/-}$ & *Slc29a3*$^{+/+}$ sample groups. Illustration of supervised PLS-DA and OPLS-DA score plots (green dots: *Slc29a3*$^{+/+}$; red dots: *Slc29a3*$^{-/-}$) based on the liver metabolic profiles derived from LC-MS-positive and -negative mode analysis among the *Slc29a3*$^{+/+}$ and *Slc29a3*$^{-/-}$ groups. PLS-DA and OPLS-DA modeling was assessed based on R2X, R2Y and Q2 values with R2Y/Q2 closer to 1 considered as a reliable model. The model parameters for PLS-DA analysis were R2X (Cum) = 0.87, R2Y = 1.00, and Q2 = 0.91 while those for OPLS-DA analysis were R2X (Cum) = 0.63, R2Y = 0.99, and Q2 = 0.91. The shaded region within the score plots indicates 95 % confidence intervals (e, above). Scatter plot (S-Plot) of the OPLS-DA model. The x-axis (p [1]) represents the relative abundance of features, while the y-axis (p corr [1]) represents the correlation of each feature to the model. Thirty features were identified as significantly altered with a cut-off value of p corr > |0.8| (e, middle). Top 15 significantly different features between the two groups ranked by PLSDA modeling based on the VIP projection scores (> 2.7). The colored boxes represent the relative levels (red: high level; green: low level) of the corresponding feature in each group (e, below). Volcano plot of all detected features constructed from the LC-MS-positive and -negative mode profiling analysis. The horizontal line indicates the significance level (p-value), while the region between the two vertical lines signifies a fold-change < 2.0. The features in the upper right and left quadrant represent metabolites that are significantly increased and decreased, respectively, in the livers of *Slc29a3*$^{-/-}$ mice (f). Statistical analyses were performed by two-tailed Student's t test. *P < 0.05. Source data are provided as a Source Data file.

Supplementary Fig. 8

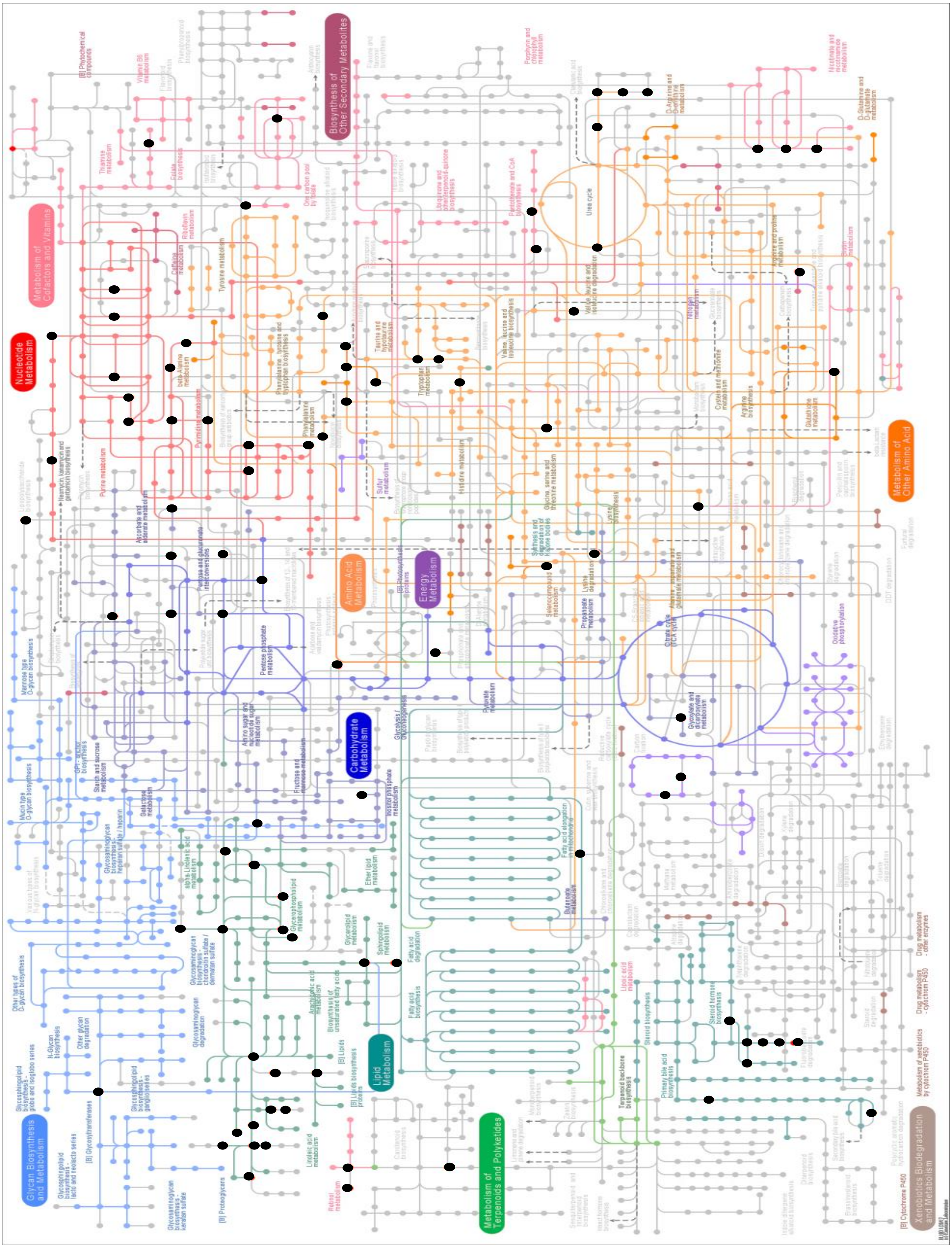

Supplementary Fig. 8: Metabolic module analysis map of the *Slc29a3*$^{-/-}$ mouse liver. The metabolic module analysis map constructed with the 180 significantly altered metabolites from the untargeted metabolomics analysis of the *Slc29a3*$^{-/-}$ mouse liver samples. This module shows a strong grouping of altered metabolites in lipid and nucleotide metabolism pathways. Metabolites that are significantly altered in the *Slc29a3*$^{-/-}$ mouse liver are indicated (black dots).

Supplementary Fig. 9

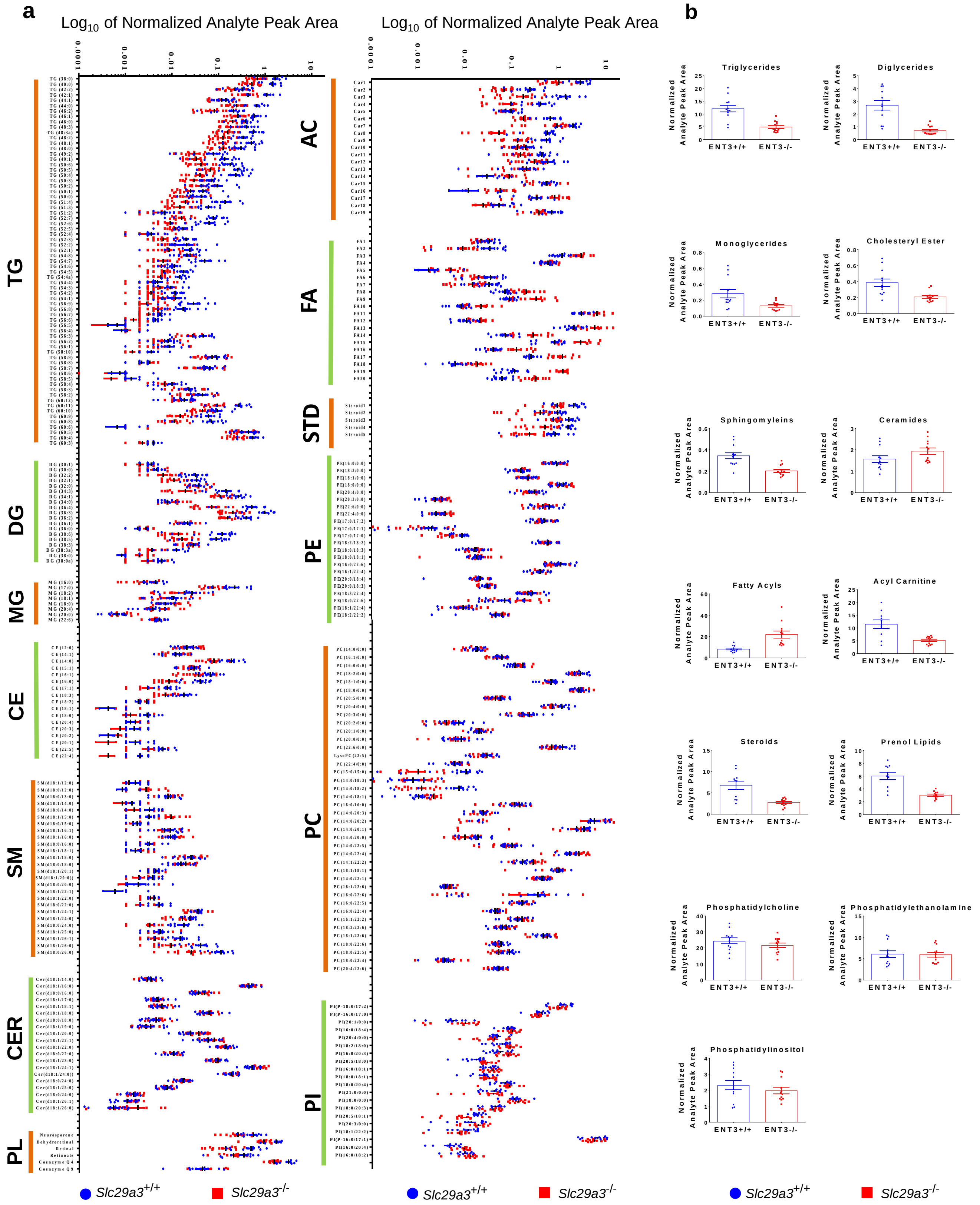

Supplementary Fig. 9: Mass spectrometry-based targeted lipidomic analyses of the *Slc29a3*$^{-/-}$ liver. The relative levels of ~500 quantified lipids expressed in the logarithmic scale (Log10) and arranged according to their respective lipid class (TG: triglycerides, DG: diglycerides, MG: monoglycerides, CE: cholesteryl esters, SP: sphingomyelins, Cer: ceramides, FA: fatty acyls, AC: acylcarnitines, STD: steroids, PL: prenol lipids, PC: phosphatidylcholine, PE: phosphatidylethanolamine and PS: phosphatidylserine). The relative levels were calculated in terms of the normalized analyte peak area obtained by dividing the MS peak area of each assayed lipid with the internal standard MS peak area. (n=12, mean±SEM) (a). Total relative levels of the 13 quantified lipid classes (b). The total relative levels are the sum of the individual levels of quantified lipids within each lipid class. Statistical analyses were performed using two-tailed Student's t test. *P < 0.05. Source data are provided as a Source Data file.

Supplementary Fig. 10

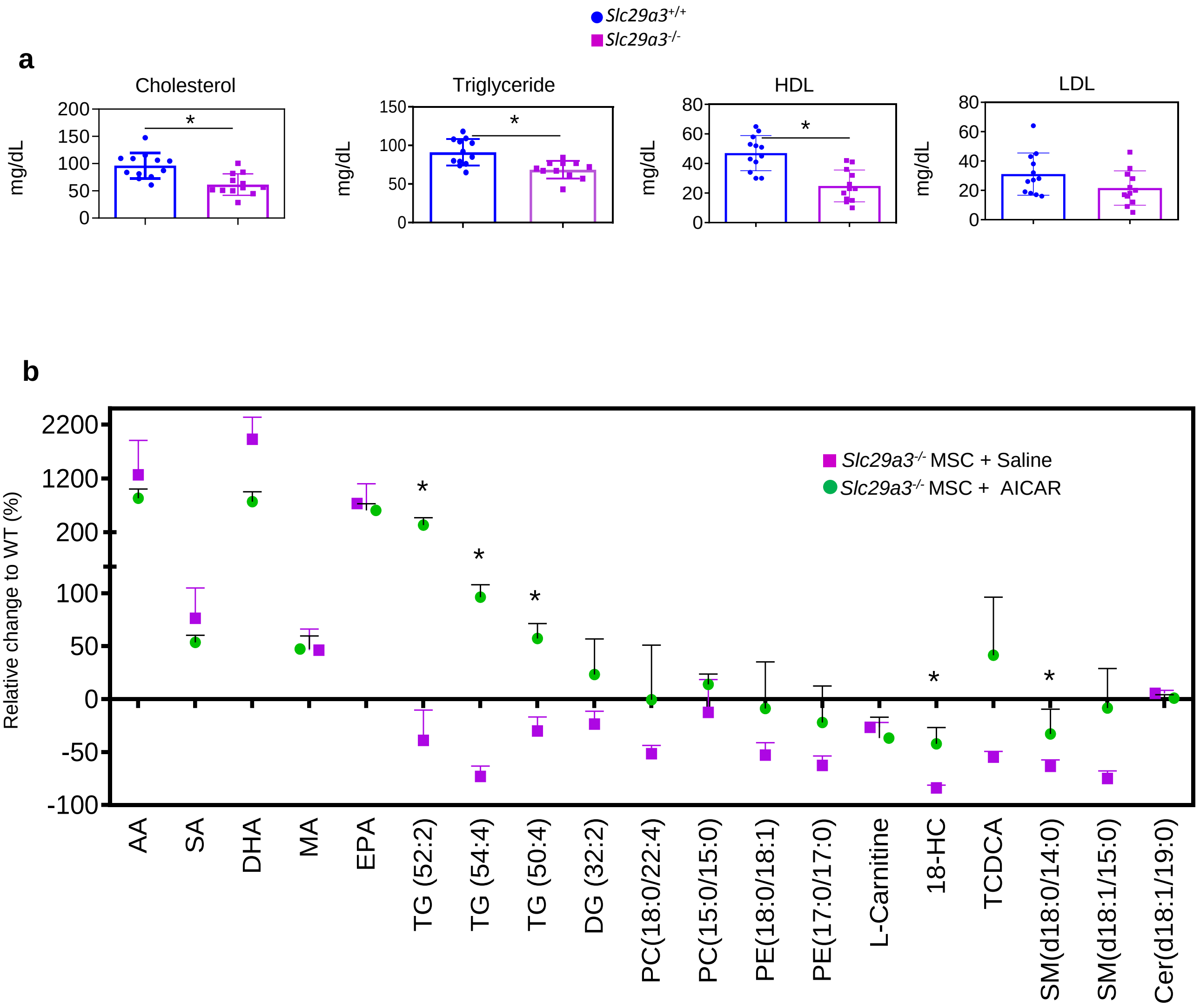

Supplementary Fig. 10: Lipid analysis in *Slc29a3*$^{-/-}$ mouse plasma and stem cells. Biochemical analysis of plasma lipid stores (n=12) (a) and targeted mass spectrometry-based relative quantification of lipids in *Slc29a3*$^{-/-}$ MSCs (n=3, mean±SEM) (b) from 12-week-old mice. Statistical analyses were performed using two-tailed Student's t test. *P < 0.05. AA, arachidonic acid; SA, stearic acid; DHA, docosahexaenoic acid; MA, myristic acid; EPA, eicosapentaenoic acid; TG, triglyceride; DG, diglyceride; PC, phosphatidylcholine; PE, phosphatidylethanolamine; HC, hexanoylcarnitine; TCDCA, taurochenodeoxycholic acid; SM, sphingomyelin; Cer, ceramide. Source data are provided as a Source Data file.

Supplementary Fig. 11

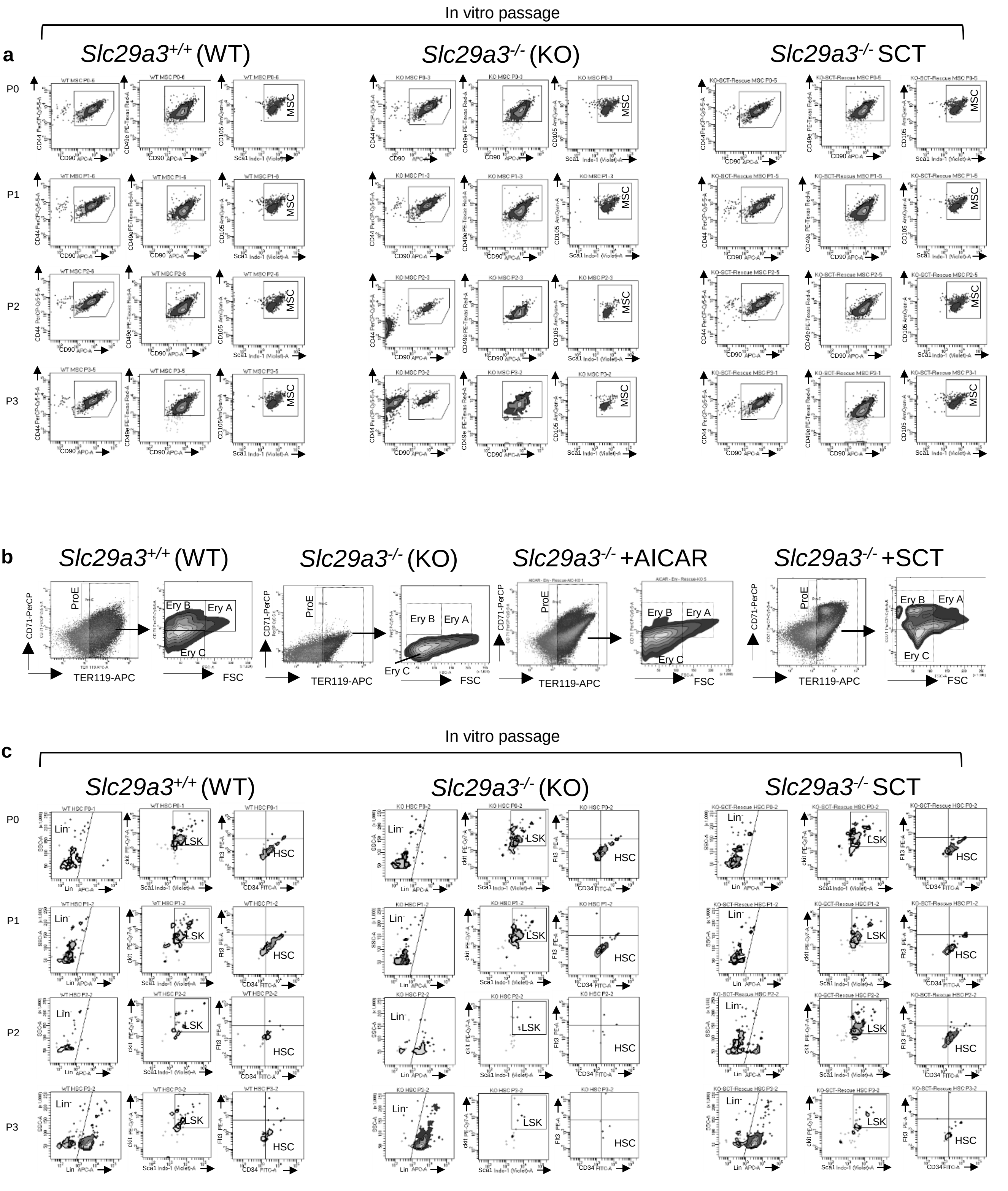

Supplementary Fig. 11: Flow cytometry analysis of serially passaged progenies of MSCs and HSCs derived from SCT mice and erythroid cell composition in AICAR-treated and SCT mice. Representative flow cytometric contour plots showing $CD44^+$ $CD49e^+$ $Sca1^+$ $CD90^+$ $CD105^+$ frequencies in serially passaged progenies (P0-P3) derived from the MSCs of WT, KO and SCT mice (a). Representative flow cytometric density plots showing the erythroid cell composition ($CD71^{high}Ter119^{med}$ proerythroblasts (ProE), $CD71^{high}Ter119^{high}FSC^{high}$ basophilic (Ery.A), $CD71^{high}Ter119^{high}FSC^{low}$ late basophilic and polychromatic (Ery.B) and $CD71^{low}Ter119^{high}FSC^{low}$ orthochromatic/reticulocyte (Ery.C) erythroblasts) of the bone marrow in AICAR-treated and SCT mice (b). Representative flow cytometric contour plots showing Lin^- $Sca1^+$ $c\text{-}kit^+$ $FLT3^-$ $CD34^+$ frequencies in serially passaged progenies (P0-P3) generated from the HSCs of WT, KO and SCT mice (c). Gating strategy to identify erythroid subpopulations, MSCs and HSCs from the total cell population are indicated by arrows and boxes.

Supplementary Fig. 12

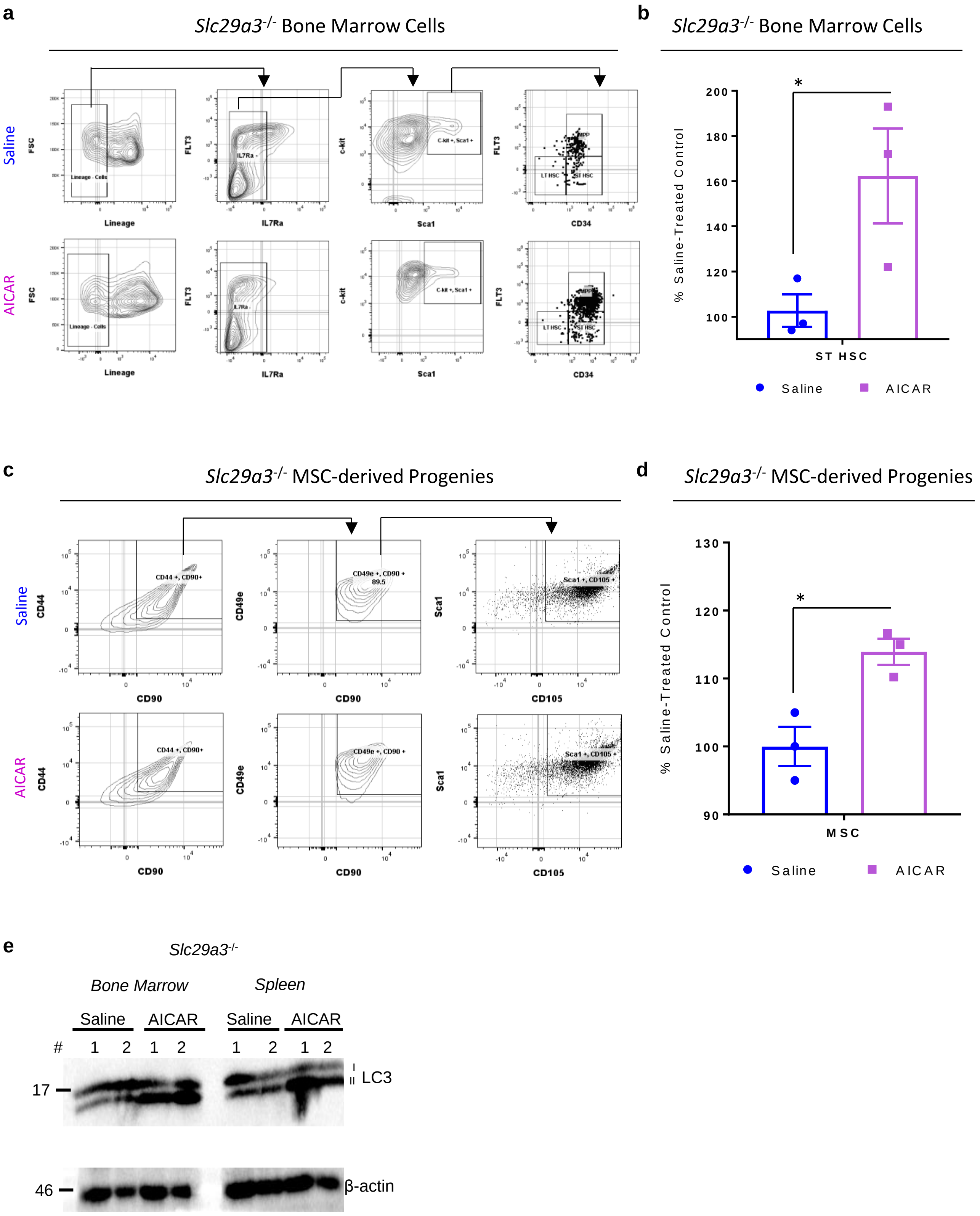

Supplementary Fig. 12: HSC and MSC analysis and LC3 assessment in short-term AICAR treated *Slc29a3*$^{-/-}$ mice. *Slc29a3*$^{-/-}$mice at 10 weeks old were injected with AICAR (500 mg/kg; SID; intraperitoneally) for 6 weeks and bone marrow samples from surviving mice were examined for HSCs and MSCs (n=8/group). Representative flow cytometric contour plots show the Lin^{-} IL7Ra^{-} Sca1^{+}c-kit^{+} HSC pool post c-kit enrichment isolated from saline and AICAR-treated *Slc29a3*$^{-/-}$mice (a). Plots show ST HSC (CD34^{+} and FLT3^{-}) frequencies in c-kit enriched bone marrow cells from saline and AICAR-treated *Slc29a3*$^{-/-}$mice (n=3; mean±SEM) (b). Freshly harvested bone marrow cells were cultured in StemSpan media for three days, and after this period, cellular debris and floating cells were removed, and the remaining MSC pool was cultured for an additional seven days. The adherent MSC pool was subsequently stained for MSC markers. Representative flow cytometric contour plots show the CD44^{+} CD49e^{+} Sca1^{+} CD90^{+} CD105^{+} MSC pool isolated from saline and AICAR-treated *Slc29a3*$^{-/-}$mice (c). Plots indicate relative MSC frequencies in saline and AICAR-treated *Slc29a3*$^{-/-}$mice (n=3; mean±SEM) (d). Western blotting analysis of lysates prepared from bone marrow and splenic samples derived from saline and AICAR-treated *Slc29a3*$^{-/-}$mice (n=2) (e). Gating strategies for both MSCs and HSCS are indicated with arrows and boxes. β-actin was used as an internal loading control. *P < 0.05. Source data are provided as a Source Data file.

Supplementary Fig. 13

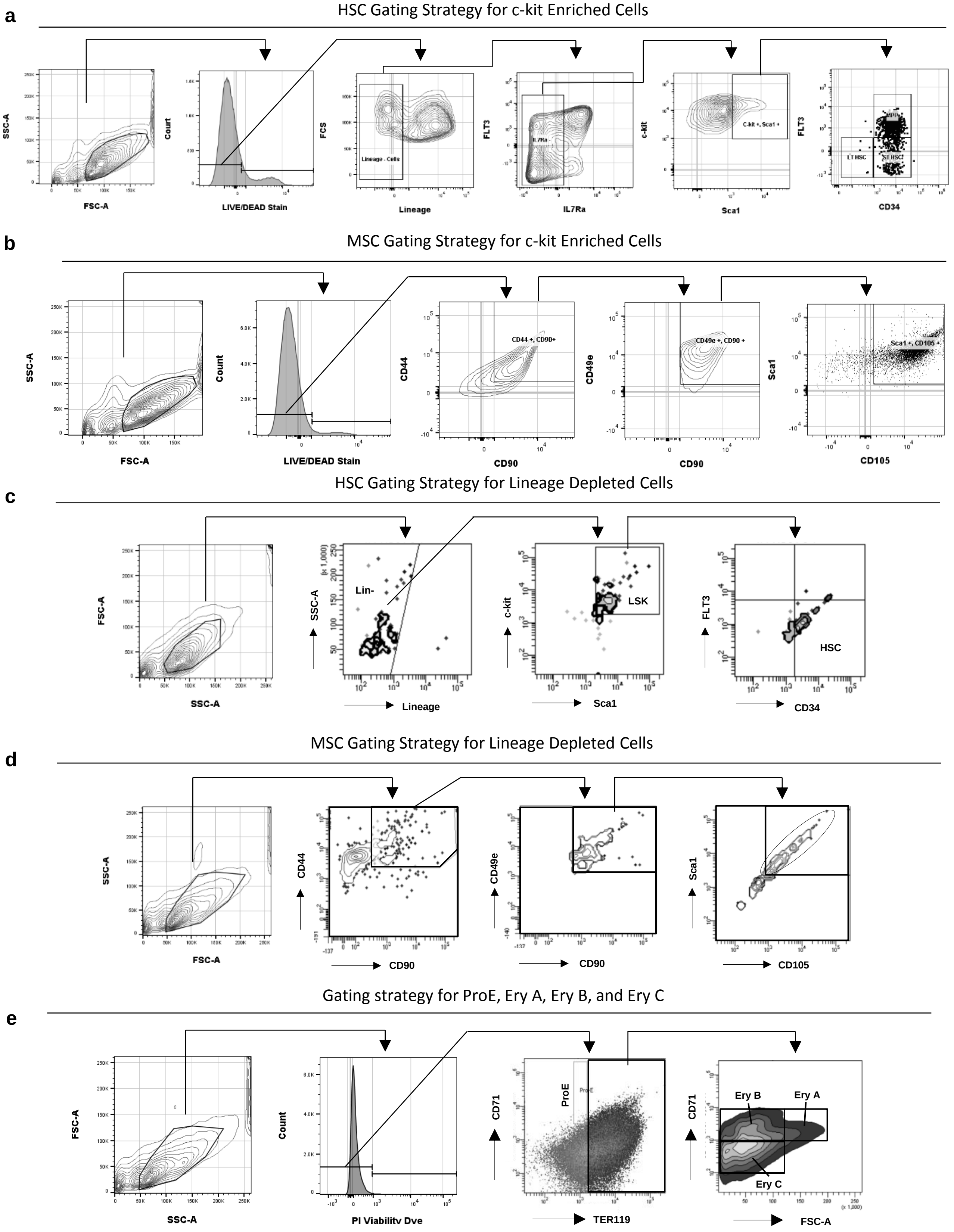

Supplementary Fig. 13: Gating strategies for HSC, MSC and ProE/EryA/EryB/EryC flow cytometry analysis. Gating strategy for c-kit enriched HSC populations (Lin$^-$, IL7Ra$^-$, c-kit$^+$, Sca1$^+$, FLT3$^-$, CD34$^+$) from *Slc29a3*$^{+/+}$ and *Slc29a3*$^{-/-}$ mice presented in Figure 3f and Supplemental Figure 3b, 4a, and 12a-b (a). Gating strategy for c-kit enriched MSC populations (CD44$^+$, CD90$^+$, CD49e$^+$, Sca1$^+$, CD105$^+$) from *Slc29a3*$^{+/+}$ and *Slc29a3*$^{-/-}$ mice presented in Supplemental Figure 12c-d (b). Gating strategy for lineage depleted HSC populations (Lin$^-$, c-kit$^+$, Sca1$^+$, FLT3$^-$, CD34$^+$) from *Slc29a3*$^{+/+}$ and *Slc29a3*$^{-/-}$ mice presented in Figure 3d-e, 3g-k, 4a-b, 4d, 5a, 5d, 9m, 9o-p and Supplemental Figure 4b and 11c (c). Gating strategy for lineage depleted MSC populations (CD44$^+$, CD90$^+$, CD49e$^+$, Sca1$^+$, CD105$^+$) from *Slc29a3*$^{+/+}$ and *Slc29a3*$^{-/-}$ mice presented in Figure 3b, 3e-f, 3i-k, 4a-g, 5a, 5d-g, 8a-g, 9g-j, and Supplemental Figure 3a, 4b, 5k-l, 10b and 11a (d). Gating strategy for ProE (CD71^{high}, TER119^{medium}, Ery A (TER119^{high}, CD71^{high}, FSC^{high}), Ery B (TER119^{high}, CD71^{high}, FSC^{low}), and Ery C (TER119^{high}, CD71^{low}, FSC^{low}) cells from *Slc29a3*$^{+/+}$ and *Slc29a3*$^{-/-}$ mice presented in Fig. 9l and Supplemental Figure 4b and 11b (e).

Supplementary Methods

Self-renewal capacity assessment of MSCs and HSCs

Freshly isolated MSCs were allowed to adhere and expand in stem cell medium. The number of colonies formed from 100 MSCs was counted 14 days after plating. The progenies derived from the initial population were examined for the expression of MSC markers by flow cytometry and were expressed as % MSCs. To assess HSC self-renewal capacity, the cells were allowed to expand in HSC medium containing 10% serum substitute, SCF (100 ng/ml) (R&D systems), IL3 (20 ng/ml) (R&D systems) and thrombopoietin (TPO, 20 ng/ml, Pepro Tech, Rocky Hill, NJ) [HSC expansion medium]. The progenies derived from the initial population were examined for the expression of HSC markers by flow cytometry and were expressed as % HSCs.

Differentiation of stem cells

Adipogenic or osteoblastogenic differentiation of MSCs was induced using established methods[55]. Briefly, MSCs were induced to differentiate into adipocytes by 3 cycles of 2 days of adipogenic induction medium (complete α-MEM containing 1 µM rosiglitazone, 5 µg/mL of insulin, 0.1 nM dexamethasone, 50 µg/mL of ascorbic acid, and 60 µM indomethacin) and 2 days of maintenance medium (complete α-MEM containing 1 µM rosiglitazone and 5 µg/mL of insulin) and were kept in maintenance medium beyond 12 days. Lipid accumulation was confirmed by oil-red O staining[55]. Osteoblast differentiation was induced by α-MEM

supplemented with 50 µg/ml of ascorbic acid and 10 mM β-glycerophosphate for 14 days. Mineralization was performed using Alizarin red-S staining[55]. Chondrocyte differentiation was induced using a commercial StemPro chondrogenesis differentiation kit (Thermoscientific, Waltham, MA). Alcian blue staining was used to detect collagen production with chondrocyte differentiation[56]. Myocyte differentiation was induced by treating MSCs with 2% horse serum[57]. Cell nuclei were counterstained with 1 µg/ml of Hoechst 33258 (Sigma-Aldrich).

HSCs were cultured in complete methylcellulose media, and their differentiation potential was assessed by colony-forming cell assays (MethoCult GF M3434 (Stem Cell Technologies)). Burst-forming unit-erythroid (BFU-E), colony-forming unit-macrophage (CFU-M), colony-forming unit-granulocyte (CFU-G) and colony-forming unit-granulocyte/macrophage (CFU-GM) were characterized and scored according to their morphology [58]. Osteoclast differentiation of HSCs was induced by 30 ng/ml of M-CSF (R&D Systems) and 50 ng/ml of RANKL (R&D Systems) for 14 days [53]. On day 14, osteoclasts were fixed and stained for tartrate-resistant acid phosphatase (TRAP) using a leukocyte acid phosphatase kit (Sigma-Aldrich, St. Louis, MO) or to visualize actin ring formation using tetramethylrhodamine-phalloidin (Life Technologies, Carlsbad, CA) as described previously [53].

Lentiviral production and transduction and real-time PCR analysis of gene expression

Lentiviruses harboring mouse *Slc29a3* and human ATG7 were produced in HEK293T cells by transfecting the target plasmids and packaging plasmids (Trans-Lentiviral ORF Packaging Kit;

Dharmacon, IL, USA) using the calcium phosphate transfection method according to the manufacturer's instructions. MSCs were transduced with lentivirus (10 MOI containing 10^8 transduction units, TU) in the presence of 8 μg/ml polybrene in α-MEM containing 1% L-glutamine for 24 h. The gene expression was confirmed by measuring the transcript levels of target genes at 2 days after transduction. Knockout MSCs transduced with RFP lentivirus served as the transduction control, and WT mouse MSCs were used to detect the basal expression of ENT3 or ATG7.

HSCs were transduced with lentivirus (10 MOI containing 10^8 transduction units, TU) in the presence of 8 μg/ml of polybrene in HSC medium in polypropylene tubes pre-coated with 1% bovine serum albumin (BSA), centrifuged at 1500 g for 3 h at 32 °C and incubated for 24 h without centrifugation. The gene expression was confirmed by measuring the transcript levels of target genes at 2 days after transduction.

The primers used for the relative estimation of transcripts by qPCR (SybrGreen assay) are as follows: Slc29a3-Forward: GGGCATATAAACTCCGAAACTGC; Slc29a3-Reverse: GGAGGAAGTATCCACCTTCACC; ATG7-Forward: TCTGGGAAGCCATAAAGTCAGG; ATG7-Reverse: GCGAAGGTCAGGAGCAGAA

Stem cell transplantation into irradiated mice

To measure the radiation rescue effects of ENT3 in MSCs and HSCs, 8-week-old *C57BL6/J* mice were exposed to total body irradiation with 9.5 Gy using an RS 2000 X-ray irradiator or 10.5 Gy using a Small Animal Radiation Research Platform (SARRP) system at the OSU Small Animal Imaging Core (SAIC) laboratory. Within 2 h after radiation, the mice were intravenously (tail vein) injected with 1×10^4 bone marrow-derived HSCs and/or 5×10^5 MSCs (derived from 8-week-old mice) after forced expression of ENT3 or RFP prior to transplantation.

Large-scale production of HSCs and MSCs for in vitro experiments

A total of 10^5 MSCs was used for each control or treatment condition in experiments that involved Sea-horse or Western blotting analyses. To generate this cell number, pooled MSCs (isolated as described above) derived from 2-3 mice were utilized to meet the above requirement.

A total of 5×10^4 HSCs was used for each control or treatment condition in Western blotting experiments. To generate this cell number, pooled HSCs (isolated as described above) derived from 5-6 mice were utilized to meet the above requirement. Prior to the experiment, freshly isolated HSCs were allowed to expand for a brief period of 7 days (without passage) in HSC expansion medium such that the purity was maintained in all groups as assessed by flow cytometry.

Western blotting analysis

Western blotting analysis was conducted as described previously.[20] Each immunoblot (for MSC and HSC experiments) was sequentially probed with 4-6 antibodies by stripping the pre-existing antibodies and re-probing with the next antibody. Uncropped blots are included in the Source Data file.

Mitochondrial bioenergetics

MSCs were plated in XF96 tissue culture plates and incubated in stem cell media. After attachment of MSCs to the substratum, cells in each well were monitored for uniform spreading and confluency. Additional wells of cells were plated for temperature correction. Growth media was replaced with unbuffered DMEM and the oxygen consumption rate and extracellular acidification rate were measured using the Seahorse XF96 flux analyzer (Agilent Technologies, Santa Clara, CA United States) following the manufacturer's protocol. Additional measurements were performed after injection of oligomycin (1 μM), carbonyl cyanide 4-trifluoromethoxy-phenylhydrazone (FCCP) (300 nM), antimycin A (0.3 mM), and rotenone (1 μM). Upon completion of the Seahorse XF24 Flux analysis, cells were lysed and total cellular protein content was estimated to normalize results to values obtained in WT MSCs.

Adeno-associated virus transduction

Recombinant adeno-associated viruses (AAV) harboring GFP (pAAV-CMV-GFP) and mENT3 (pAAV-EF1a-Slc29a3) mEnt3 were generated as per manufacturer's instructions (Applied Biological Materials Inc). Briefly, HEK293 cells at a concentration of 1 x 10^6 cells/10 cm dish were seeded and complete transfection complex (prepared using 10 µg of pAAV-CMV-GFP or pAAV-EF1a-Slc29a3 plasmids and 30 µg of AAV packaging mix as per manufacturer's protocol) was added to dishes and incubated at 37°C for 8 h in serum-free and antibiotic-free media. The cells were subsequently incubated in the presence of serum and continuously monitored for cytopathic effects. At the end of 5 days (or after reaching confluency), cells were trypsinized, washed with PBS, and centrifuged at 3000 rpm for 15 min at 4°C to isolate cell pellets. Cell pellets were later freeze-thawed for three times at -80°C and 37°C to release the virus into the supernatant and centrifuged at 10,000 rpm for 20 minutes. The cleared supernatant was filtered with a low-protein binding 0.45 µM sterile filter and AMPK α1/α2 double-knockout MEFs were directly infected with viral samples at an MOI of 50,000 for 12 h. Expression of mENT3 was confirmed using qPCR analysis after 5 days of infection.

Cell viability assay

Colorimetry-based MTT reduction depending on cellular mitochondrial dehydrogenase activity was used to assess cell viability. To this end, 10^4 cells were plated in each well of a 96-well plate for each treatment (or control), after which MTT at a final concentration of 1 µg/ml

was added and incubated at 37°C in a CO_2 incubator for 4 h. The formazan product formed within the cells was dissolved in DMSO after the separation of cells using a plate centrifuge. Absorption at 540 nm with background correction at 650 nm was measured using a spectrophotometer. In preliminary experiments, the linearity between cell viability and formazan product formation was confirmed using wild-type MSCs and HSCs. Optical density values were ensured to be within the linear range by serial dilutions of products derived from control cells.

To study cell death program, we further combined a caspase-3 based cell death (apoptosis) assay to MTT cell viability assay. The concentration of RAPA or AICAR that exhibited maximal cell viability effects (by MTT assay) was again examined for % apoptotic cells by flow cytometry using a caspase-3 FITC conjugate. 10^6 cells were treated for 4 h with camptothecin to induce apoptosis, permeabilized, fixed and stained for active caspase-3 as indicated in FITC Active Caspase-3 Apoptosis Kit (BD Biosciences). Results were expressed as fold change relative to WT cells.

In vitro transcription and expression of del36ENT3 in Xenopus oocytes

The details of pOX-Δ36hENT3 Xenopus expression constructs were described previously[18, 20]. The plasmids were linearized by the Not1 restriction enzyme and purified by phenol-chloroform extraction. The capped mRNAs were synthesized using mMESSAGE mMACHINE (Ambion) transcription kits following the manufacturer's instructions and purified

using the lithium precipitation method. Fifty nanoliters (400 ng/μl) of del36ENT3 mRNA was injected into defolliculated oocytes and was incubated at 15 °C for 24 h before conducting the AICAR transport assay. Uptake of AICAR (20 μM) was measured after 30 min of incubation in transport buffer (100 mm NaCl, 2 mm KCl, 1 mm $CaCl_2$, 1 mm $MgCl_2$, and 10 mm HEPES, pH 7.4) at 28°C. Uptake was stopped by washing the oocytes 3 times with arrest buffer[22] followed by washing 3 times in PBS, and the uptake of AICAR was quantified by LC-MS/MS.
