# Supplementary material for: Adult stem cell deficits drive Slc29a3 disorders in mice
Source: Nat Commun. 2019 Jul 3;10:2943. doi: 10.1038/s41467-019-10925-3 (PMC6610100; doi:10.1038/s41467-019-10925-3)
Supplement: Supplementary file 2 — Reporting Summary [file 41467_2019_10925_MOESM2_ESM.pdf]

nature research

Corresponding author(s): Rajgopal Govindarajan, PhD

Last updated by author(s): 4/26/2019

Reporting Summary

Nature Research wishes to improve the reproducibility of the work that we publish. This form provides structure for consistency and transparency in reporting. For further information on Nature Research policies, see Authors & Referees and the Editorial Policy Checklist.

Statistics

For all statistical analyses, confirm that the following items are present in the figure legend, table legend, main text, or Methods section.

n/a	Confirmed	
☐	☒	The exact sample size (*n*) for each experimental group/condition, given as a discrete number and unit of measurement
☐	☒	A statement on whether measurements were taken from distinct samples or whether the same sample was measured repeatedly
☐	☒	The statistical test(s) used AND whether they are one- or two-sided<br>*Only common tests should be described solely by name; describe more complex techniques in the Methods section.*
☐	☒	A description of all covariates tested
☐	☒	A description of any assumptions or corrections, such as tests of normality and adjustment for multiple comparisons
☐	☒	A full description of the statistical parameters including central tendency (e.g. means) or other basic estimates (e.g. regression coefficient) AND variation (e.g. standard deviation) or associated estimates of uncertainty (e.g. confidence intervals)
☐	☒	For null hypothesis testing, the test statistic (e.g. F, t, r) with confidence intervals, effect sizes, degrees of freedom and P value noted<br>*Give P values as exact values whenever suitable.*
☒	☐	For Bayesian analysis, information on the choice of priors and Markov chain Monte Carlo settings
☐	☒	For hierarchical and complex designs, identification of the appropriate level for tests and full reporting of outcomes
☒	☐	Estimates of effect sizes (e.g. Cohen's *d*, Pearson's *r*), indicating how they were calculated

Our web collection on statistics for biologists contains articles on many of the points above.

Software and code

Policy information about availability of computer code

Data collection	Image J software for TEM and confocal image morphometric quantification, Mzmine for mass spectrometry data deconvolution, and Metaboanalyst for mass spectrometry data statistical analysis were used. Graphpad Prism (version 7) was used for all other statistical analyses. Image Lab software 5.2.1 was used for Western blotting analysis.
Data analysis	Image J software for TEM and confocal image morphometric quantification, Mzmine for mass spectrometry data deconvolution, and Metaboanalyst for mass spectrometry data statistical analysis were used. Graphpad Prism (version 7) was used for all other statistical analyses. Image Lab software 5.2.1 was used for Western blotting analysis.

For manuscripts utilizing custom algorithms or software that are central to the research but not yet described in published literature, software must be made available to editors/reviewers. We strongly encourage code deposition in a community repository (e.g. GitHub). See the Nature Research guidelines for submitting code & software for further information.

Data

Policy information about availability of data

All manuscripts must include a data availability statement. This statement should provide the following information, where applicable:

- Accession codes, unique identifiers, or web links for publicly available datasets
- A list of figures that have associated raw data
- A description of any restrictions on data availability

The following statement is provided: The data sets generated during and/or analyzed during the current study are available within the article and its Supplementary Information files, or from the corresponding author on reasonable request. A list of figures that have associate raw data is indicated in the respective figure legends. A reporting summary for this Article is available as a Supplementary Information file. The source data underlying Figs 1-9 and Supplementary Figs 4-7, 10 and 12 are provided as a Source Data file

Field-specific reporting

Please select the one below that is the best fit for your research. If you are not sure, read the appropriate sections before making your selection.

☒ Life sciences ☐ Behavioural & social sciences ☐ Ecological, evolutionary & environmental sciences

For a reference copy of the document with all sections, see nature.com/documents/nr-reporting-summary-flat.pdf

Life sciences study design

All studies must disclose on these points even when the disclosure is negative.

Sample size	For all in vitro experiments, a sample size of n=3-12 was used based on the effect size and overlap between distributions. A power of 0.8 was set as minimal to decide sample size for each experiment. For in vivo studies that assessed the effects of AICAR treatment or stem cell transplant on survival of Slc29a3(-/-) mice, a sample size of n=10-16 independent mice per group was used to include inherent variabilities in survival times among Slc29a3(-/-) mice. Sample size estimation was initially chosen by using power calculations for guidance (http://biomath.info/power/ttest.htm). Effect sizes were estimated with additional guidance from pilot studies. With alpha=0.05 and power=0.9 and allowing for unexpected mortalities of ~10%/group, ≥8 mice per group were needed for in vivo studies. For in vivo bone marrow transplant mice study, a sample size of n=6 independent mice per group was set because a definitive end point mortality within 7 days post irradiation was achieved in all non-transplanted mice. The sample size for the metabolomics and lipidomics experiments was calculated based on the power analysis module in Metaboanalyst software (Reference 64), which uses algorithms described by van Iterson et al. 2013. A desired power of 0.8 was achieved for both the metabolomics and lipidomics data validating the sample size of n=12 independent mice per group (Methods section, Statistical analyses subsection, last paragraph).
Data exclusions	No data were excluded from the analysis (Method section, Statistical analyses subsection, last paragraph, last line).<br>Note: For in vivo studies that assessed the effects of AICAR treatment on survival of Slc29a3(-/-) mice, one mouse in AICAR treated group exhibited profound ulcerations around an eye. While an enucleation surgery was performed on this mice to continue in the study, profuse bleeding called for euthanasia of this mouse on day 158. The data point for this mouse was censored to 158 days survival in the Kaplan-Meier analysis plot.
Replication	All attempts to reproduce results were successful.
Randomization	For all studies using mice and mouse tissues, animals of either sex and similar age were randomly arranged into groups. This allocation method allowed us to generate homogeneous blocks for a randomized block design.
Blinding	For scoring autophagosomes and/or lysosomes in TEM studies, slide identities were blinded and predefined stereoptic criteria were used to quantify structures of interest (Methods section, Transmission electron microscopy subsection, last two lines). End point measures and outcomes for all animal experiments were objective (survival times) that did not necessitate blinding in animal experiments.

Reporting for specific materials, systems and methods

We require information from authors about some types of materials, experimental systems and methods used in many studies. Here, indicate whether each material, system or method listed is relevant to your study. If you are not sure if a list item applies to your research, read the appropriate section before selecting a response.

Materials & experimental systems

n/a	Involved in the study	
☐	☒	Antibodies
☐	☒	Eukaryotic cell lines
☒	☐	Palaeontology
☐	☒	Animals and other organisms
☒	☐	Human research participants
☒	☐	Clinical data

Methods

n/a	Involved in the study	
☒	☐	ChIP-seq
☐	☒	Flow cytometry
☒	☐	MRI-based neuroimaging

Antibodies

Antibodies used	Goat polyclonal antibodies against carboxyl (C20; sc-48147); or amino (N18; sc-48149) terminus of hENT3 were obtained from Santa Cruz Biotechnology (Santa Cruz, CA). (website currently not available)<br>A rabbit polyclonal antibody generated against the third intracellular loop of hENT3 was also used. These three antibodies were characterized in our prior studies (Am J Physiol Gastrointest Liver Physiol. 2009 Apr;296(4):G910-22 and J Biol Chem. 2010 Sep 3;285(36):28343-52).<br>A rabbit polyclonal anti-hENT3 antibody (PA5-38039) was purchased from Thermo Scientific (Waltham, MA); https://www.thermofisher.com/antibody/product/SLC29A3-Antibody-Polyclonal/PA5-38039.<br>Rabbit monoclonal antibodies against LC3B (3868S); https://www.cellsignal.com/products/primary-antibodies/lc3b-d11-xp-rabbit-mab/3868?N=4294956287&Ntt=3868s&fromPage=plp&_requestid=325599,

Anti-pmTOR (5536S); https://www.cellsignal.com/products/primary-antibodies/phospho-mtor-ser2448-d9c2-xp-rabbit-mab/5536?N=4294956287&Ntt=5536s&fromPage=plp&_requestid=325619,
Anti-mTOR (2983S); https://www.cellsignal.com/products/primary-antibodies/mtor-7c10-rabbit-mab/2983?_=1496267927041&Ntt=2983s&tahead=true,
pAKT (4060S); https://www.cellsignal.com/products/primary-antibodies/phospho-akt-ser473-d9e-xp-rabbit-mab/4060?N=4294956287&Ntt=4060s&fromPage=plp&_requestid=325653,
Anti-AKT (4685S); https://www.cellsignal.com/products/primary-antibodies/akt-pan-11e7-rabbit-mab/4685?N=4294956287&Ntt=4685s&fromPage=plp&_requestid=325668,
Anti-p4EBP1 (2855S); https://www.cellsignal.com/products/primary-antibodies/phospho-4e-bp1-thr37-46-236b4-rabbit-mab/2855?N=4294956287&Ntt=2855s&fromPage=plp&_requestid=325747,
Anti-4EBP1 (9644S); https://www.cellsignal.com/products/primary-antibodies/4e-bp1-53h11-rabbit-mab/9644?N=4294956287&Ntt=9644s&fromPage=plp&_requestid=326439,
Anti-PS6K (9234S); https://www.cellsignal.com/products/primary-antibodies/phospho-p70-s6-kinase-thr389-108d2-rabbit-mab/9234?N=4294956287&Ntt=9234s&fromPage=plp&_requestid=326672,
Anti-S6K (2708S); https://www.cellsignal.com/products/primary-antibodies/p70-s6-kinase-49d7-rabbit-mab/2708?N=4294956287&Ntt=2708s&fromPage=plp&_requestid=326865,
Anti-pULK1-S555 (5869S); https://www.cellsignal.com/products/primary-antibodies/phospho-ulk1-ser555-d1h4-rabbit-mab/5869?N=4294956287&Ntt=5869s&fromPage=plp&_requestid=327028,
Anti-pULK1-S757 (14202S); https://www.cellsignal.com/products/primary-antibodies/phospho-ulk1-ser757-d7o6u-rabbit-mab/14202?N=4294956287&Ntt=14202s&fromPage=plp&_requestid=327283,
Anti-ULK1 (6439S); https://www.cellsignal.com/products/primary-antibodies/ulk1-d9d7-rabbit-mab/6439?N=4294956287&Ntt=6439s&fromPage=plp&_requestid=327446,
Anti-pACC (11818S); https://www.cellsignal.com/products/primary-antibodies/phospho-acetyl-coa-carboxylase-ser79-d7d11-rabbit-mab/11818?N=4294956287&Ntt=11818s&fromPage=plp&_requestid=327655,
Anti-ATG7 (8558S); https://www.cellsignal.com/products/primary-antibodies/atg7-d12b11-rabbit-mab/8558?N=4294956287&Ntt=8558s&fromPage=plp&_requestid=327781,
Anti-Beclin1 (3495S); https://www.cellsignal.com/products/primary-antibodies/beclin-1-d40c5-rabbit-mab/3495?N=4294956287&Ntt=3495s&fromPage=plp&_requestid=327801
Rabbit polyclonal antibodies against pAMPK (2531S); https://www.cellsignal.com/products/primary-antibodies/phospho-ampka-thr172-antibody/2531?N=4294956287&Ntt=2531s&fromPage=plp&_requestid=327812,
Anti-AMPK (2532S); https://www.cellsignal.com/products/primary-antibodies/ampka-antibody/2532?N=4294956287&Ntt=2532s&fromPage=plp&_requestid=327824,
Anti-ACC (3662S); https://www.cellsignal.com/products/primary-antibodies/acetyl-coa-carboxylase-antibody/3662?N=4294956287&Ntt=3662s&fromPage=plp&_requestid=327833
Anti-ATG5 (2630S); https://www.cellsignal.com/products/primary-antibodies/atg5-antibody/2630?N=4294956287&Ntt=2630s&fromPage=plp&_requestid=327846 were obtained from Cell Signaling Technology (Beverly, MA).
A mouse monoclonal Anti-SQSTM1 (p62) antibody (610833) was purchased from BD Biosciences; http://www.bdbiosciences.com/us/reagents/research/antibodies-buffers/cell-biology-reagents/cell-biology-antibodies/purified-mouse-anti-p62-ick-ligand-3p62-lck-ligand/p/610833,
Anti-β-actin antibody (A5316) from Sigma (St. Louis, MO); http://www.sigmaaldrich.com/catalog/product/sigma/a5316?lang=en&region=US
Anti-GFP antibody (sc-9996) from Santa Cruz Biotechnology (Santa Cruz, CA); https://www.scbt.com/scbt/product/gfp-antibody-b-2?requestFrom=search.
Anti-F4/80 antibody (ab6640) was obtained from Abcam; http://www.abcam.com/f480-antibody-cia3-1-ab6640.html. Horse radish peroxidase conjugated anti-rabbit IgG (A120-201P)
Anti-mouse IgG (A90-116P) and anti-goat IgG (A50-101P) were purchased from Bethyl laboratories (Montgomery, TX); https://www.bethyl.com/.
Alexa 488 and 594 conjugated anti-rabbit, anti-mouse and anti-goat secondary antibodies were obtained from Invitrogen, Thermo scientific (Waltham, MA); https://www.thermofisher.com/search/results?query=Alexa%20fluor&focusarea=Search%20All.
Anti-LAMP1 (D2D11) were obtained from Cell Signaling Technology (Beverly, MA) (https://www.cellsignal.com/products/primary-antibodies/lamp1-d2d11-xp-rabbit-mab/9091).
Rabbit polyclonal antibodies against EEA-1, calreticulin and hsp70 mouse monoclonal antibodies were obtained from Affinity Bioreagents (Golden, CO). (Now ThermoFischer: https://www.thermofisher.com/us/en/home/life-science/antibodies.html)
Anti-β-COP antibody (ab2899) from was obtained from Abcam (https://www.abcam.com/beta-cop-antibody-ab2899.html)
Anti-tubulin (rabbit monoclonal) antibody was from Cell signaling
(https://www.cellsignal.com/products/primary-antibodies/a-tubulin-11h10-rabbit-mab/2125)
Anti-lamin B1 rabbit monoclonal antibody was from Cell signaling
(https://www.cellsignal.com/products/primary-antibodies/lamin-b1-d4q4z-rabbit-mab/12586)
Antibody conjugates for FLT3 (APC-anti-CD135), IL7Ra (V450-anti-CD127), Sca1 (PE-Cy7-anti-Ly-6A/E), and CD34 (FITC-anti-CD34) were obtained from BD Biosciences (San Jose, CA), while c-kit (PE-anti-CD117) was obtained from Miltenyi Biotech (Bergisch Galadbach, Germany). Additionally, BUV395-anti-Sca-1 and PE-Cy7-anti-c-kit were purchased from BD Biosciences (San Jose, CA). (http://www.bdbiosciences.com/us/solrSearch?text=antibodies&x=0&y=0). Antibody conjugate for PE-anti-FLT3 was obtained from eBioscience (https://www.thermofisher.com/us/en/home/life-science/antibodies/ebioscience.html).

Validation

Included in the previous box, where necessary.

Eukaryotic cell lines

Policy information about cell lines

Cell line source(s)

Retroviral (ATCC, CRL 9078) and lentiviral packaging (ATCC, CRL 11268) cell lines and HEK 293 (ATCC, CRL 1573) were purchased from American Type Culture Collection (ATCC, Manasas). These cell lines were propagated, expanded, and frozen

	immediately upon receipt. The cells revived from the frozen stock were used within 10-20 passages, not exceeding a period of 2-3 months. AMPK α1/α2 double knockout (DKO) MEFs were obtained from Dr. B. Viollet (Institut Cochin INSERM U1016, CNRS UMR 8104, Université Paris Descartes, Department of Endocrinology, Metabolism and Cancer, Paris, France (Ref: Nat Cell Biol. 2011 Feb;13(2):132-41).
Authentication	The ATCC uses morphological, cytogenetic, and DNA profile analyses for characterization of cell lines.
Mycoplasma contamination	Each cell line was tested negative for mycoplasma contamination (Methods section; Cells, Plasmids, Reagents and Antibodies subsection; first paragraph; lines 3 and 4)
Commonly misidentified lines (See ICLAC register)	No commonly misidentified cell lines were used in the study.

Animals and other organisms

Policy information about studies involving animals; ARRIVE guidelines recommended for reporting animal research

Laboratory animals	Mice of 8-20 weeks from either sex from mixed background of 129S5/SvEvBrd and C57BL6/J were used.
Wild animals	The study did not involve wild animals
Field-collected samples	The study did not involve field-collected samples
Ethics oversight	All animal procedures were performed according to protocols approved by the Ohio State University (OSU) IACUC (Methods section; Breeding, genotyping, and maintenance of Slc29a3-/- mice subsection; first line)

Note that full information on the approval of the study protocol must also be provided in the manuscript.

Flow Cytometry

Plots

Confirm that:

☒ The axis labels state the marker and fluorochrome used (e.g. CD4-FITC).

☒ The axis scales are clearly visible. Include numbers along axes only for bottom left plot of group (a 'group' is an analysis of identical markers).

☒ All plots are contour plots with outliers or pseudocolor plots.

☒ A numerical value for number of cells or percentage (with statistics) is provided.

Methodology

Sample preparation	Freshly harvested bone marrow cells were c-kit-enriched using c-kit-conjugated magnetic beads (Miltenyi, Bergisch Gladbach, Germany), and subsequently costained with antibodies against lineage markers (Lineage Cell Detection Cocktail, Miltenyi), FLT3 (APC-anti-CD135), IL7Ra (V450-anti-CD127), c-kit (PE-Cy7-anti-CD117), Sca1 (BUV395-anti-Ly-6A/E), and CD34 (PE-anti-CD34). The LIVE/DEAD Fixable Near-IR Dead Cell Stain Kit (ThermoFisher Scientific, Waltham, MA) was used to exclude dead cells. Cell staining was performed on ice and analyzed using a BD LSRFortessa equipped with five lasers (355, 405, 488, 561, and 633 nm). Color compensation was performed using BD FACSDIVA Software (BD Biosciences, Bedford, MA) and FACS data analysis was performed using FlowJo (Tree Star, Ashland, OR).<br>Similar procedures are used for MSC isolation. Briefly, the Lin- cells were allowed to adhere for 3 days in stem cell medium. The non-adherent hematopoietic cells were discarded, and the adherent populations were trypsinized and expanded for 2 weeks (in some cases shorter depending on the experiment) before analysis for MSC markers by flow cytometry (same equipment as above).
Instrument	Cell staining was performed on ice and analyzed using a BD LSRFortessa equipped with five lasers (355, 405, 488, 561, and 633 nm)
Software	Color compensation was performed using BD FACSDIVA Software (BD Biosciences, Bedford, MA) and FACS data analysis was performed using FlowJo (Tree Star, Ashland, OR).
Cell population abundance	Purity of the sorted HSC and MSC pools were confirmed by flow cytometry resulting in a purity of the sorted cells of >95%.
Gating strategy	Long-term HSC (LT HSC), short-term HSC (ST HSC), and multipotent progenitor (MPP) cell types were quantified following gating schemes described previously (Pronk C. J., Rossi D. J., Månsson R., Attema J. L., Norddahl G. L., Chan C. K., Sigvardsson M., Weissman I. L. & Bryder D. Elucidation of the phenotypic, functional, and molecular topography of a myeloerythroid progenitor cell hierarchy. Cell Stem Cell 11, 428-442, doi: 10.1016/j.stem.2007.07.005 (2007)). In short, cells exhibiting a Lin- IL7Ra- Sca1+c-kit+phenotype were resolved into LT HSC, ST HSC, or MPP cell types based on differential expression of CD34 and FLT356. Among Lin- IL7Ra- Sca1+c-kit+ cells, LT HSCs were CD34- and FLT3-, ST HSCs were CD34+ and FLT3-, and MPPs were CD34+ and FLT3+ (Adolfsson J., Borge O. J., Bryder D., Theilgaard-Mönch K., Astrand-Grundström I., Sitnicka E., Sasaki Y. & Jacobsen S. E. Upregulation of Flt3 expression within the bone marrow Lin(-)Sca1(+)c-kit(+) stem cell compartment is accompanied by loss of self-renewal capacity. Immunity 25, 659-669 (2001)).<br>BUV395-anti-Sca-1, PerCP-Cy5.5-anti-CD44, PE-CF594-anti-CD49e, APC-anti-CD90 and BV510-anti-CD105 antibodies (BD

Biosciences) were used to obtain Lin-Sca1+CD44+CD49e+CD90+CD105+ MSCs (Muruganandan, S., Govindarajan, R., McMullen, N. M. & Sinal, C. J. Chemokine-Like Receptor 1 Is a Novel Wnt Target Gene that Regulates Mesenchymal Stem Cell Differentiation. Stem Cells 35, 711-724, doi:10.1002/stem.2520 (2017)).
A representative plot and gating is provided with every flow cytometry figure.

☐ Tick this box to confirm that a figure exemplifying the gating strategy is provided in the Supplementary Information.
