# Supplementary material for: Adult stem cell deficits drive Slc29a3 disorders in mice
Source: Nat Commun. 2019 Jul 3;10:2943. doi: 10.1038/s41467-019-10925-3 (PMC6610100; doi:10.1038/s41467-019-10925-3)
Supplement: Supplementary file 3 — Description of Additional Supplementary Files [file 41467_2019_10925_MOESM3_ESM.pdf]

## Description of Additional Supplementary Files

### File name: Supplementary Data 1

**Nontargeted metabolomics of *Slc29a3*$^{-/-}$ mouse livers**
**(Supporting information for Fig. 8a)**

List of 879 differential metabolites detected in the liver tissues from *Slc29a3*$^{+/+}$ & *Slc29a3*$^{-/-}$ mice (12 weeks age) based on a p-value < 0.05 and a fold-change > 2.0. The m/z and ionization mode (positive ([M+H]+) or negative ([M-H]-)) are mentioned for all the detected metabolites. In addition to the fold change, the log2-fold change, p-value, –log 10 (p) and VIP score for each compound are indicated. The 175 chemically annotated metabolites have been categorized according to their superclass—i.e., lipids (95 compounds); amino and organic acids (33 compounds); carbohydrates (16 compounds); nucleosides, nucleotides, and analogues (15 compounds); organoheterocyclic compounds (12 compounds) and benzenoids (4 compounds).

### File name: Supplementary Data 2

**Targeted lipidomics of Slc29a3-/- mouse livers**
**(Supporting information for Fig. 8b)**

Peak area (Relative to I.S.) values of each lipid species detected in the liver from 12 weeks old *Slc29a3*$^{+/+}$ and *Slc29a3*$^{-/-}$mice. For each lipid species, the relative ratio (*Slc29a3*$^{-/-}$/*Slc29a3*$^{+/+}$) with a p-value is indicated. The species in bold represents the occurrence of a significant change (p-value < 0.05) in *Slc29a3*$^{-/-}$ mice.
